# Cross-site quantitative MRI harmonization: The impact on age modeling in health and disease

**DOI:** 10.1162/IMAG.a.1140

**Published:** 2026-02-24

**Authors:** Xinjie Chen, Mario Ocampo-Pineda, Po-Jui Lu, Clara Ekerdt, Matthias Weigel, Michelle G. Jansen, Alessandro Cagol, Kwok-Shing Chan, Sabine Schädelin, Marcel Zwiers, Joukje M. Oosterman, David G. Norris, Johanna M.M. Bayer, Andre F. Marquand, Willeke M. Menks, Jens Kuhle, Ludwig Kappos, Lester Melie-Garcia, Cristina Granziera, José P. Marques

**Affiliations:** Translational Imaging in Neurology (ThINk) Basel, Department of Biomedical Engineering, Faculty of Medicine, University Hospital Basel and University of Basel, Basel, Switzerland; Department of Neurology, University Hospital Basel, Basel, Switzerland; Research Center for Clinical Neuroimmunology and Neuroscience Basel (RC2NB), University Hospital Basel and University of Basel, Basel, Switzerland; Donders Institute for Brain, Cognition and Behaviour, Radboud University, Nijmegen, the Netherlands; Department of Pediatric Neurology and Developmental Medicine, University Children’s Hospital Basel (UKBB), Basel, Switzerland; Division of Radiological Physics, Department of Radiology, University Hospital Basel, Basel, Switzerland; Department of Health Sciences, University of Genova, Genova, Italy; Athinoula A. Martinos Center for Biomedical Imaging, Charlestown, MA, United States; Department of Radiology, Harvard Medical School, Boston, MA, United States

**Keywords:** normative modeling, brain aging, hierarchical Bayesian regression, ComBat, multiple sclerosis

## Abstract

Cross-site quantitative MRI (qMRI) studies are hindered by site-related variability, particularly when integrating cohorts with varying acquisition protocols and biological variables, such as age. Such batch effects can obscure accurate biological signals, thus affecting normative age modeling and clinical interpretations of pathology. This study aimed to (i) compare the harmonization performance of multiple strategies across multi-site cohorts with matched or unmatched qMRI protocols; (ii) quantify the impact of harmonization on normative age modeling across cohorts with comparable or partially overlapping age ranges; and (iii) examine whether clinically relevant deviations are preserved. Quantitative MRI data from three healthy control (HC) cohorts (n = 530) and one cohort of people with multiple sclerosis (pwMS) (n = 98) were included. Batch effects in the raw data were assessed using intra-class correlation coefficients (ICC) and analysis of variance (ANOVA), before and after adjustment for age and sex. Data harmonization was performed separately using Empirical Bayes-based generalized additive models (GAM) and Hierarchical Bayesian Regression (HBR) with B-spline fitting. Regional age-related effects across cortical grey matter (cGM), superficial white matter (sWM), and white matter (WM) bundles were modeled using second-degree polynomial regression, with the turning point defined as the peak age when the fitted curve reaches its maximum or minimum, and further used to assess potential shifts in age-related models between raw and harmonized datasets. For clinical validation, group-level differences in qMRI metrics between pwMS and controls were evaluated using Cohen’s d. Regional Z-scores derived from the HBR framework were then used to assess associations with clinical disability, as measured by the Expanded Disability Status Scale (EDSS). Both harmonization methods reduced site-related variance, achieving post-harmonization ICC (<0.001) and η^2^ values (<0.01) across all regions. For R_1_, harmonization resulted in small, tissue-dependent differences from raw data in estimated regional peak ages (mean difference, 1.1 years; maximum, 4.0 years in WM bundles for HBR) and reduced RMSE across tissues, with the greatest reduction observed in cGM (approximately 12%). For R_2_*, despite protocol discrepancies, the harmonized and normalized raw data yielded comparable aging patterns (peak-age difference <1 year) and reduced RMSE, most notably in cGM (approximately 15–17%). Harmonized Z-scores preserved disease-related deviations, with pwMS exhibiting a progressive increase in the extent of significant regional differences from cGM (most regions) to sWM (nearly all regions) and WM bundles (whole regions). Furthermore, R_2_* Z-scores in specific cGM regions (Brodmann areas 1, 9, 10, 37, 43, and 46) showed positive correlations with EDSS scores in pwMS. This study suggests that both GAM and B-spline models effectively reduce site effects and maintain consistency in normative age modeling across multi-site qMRI datasets. HBR-based Z-scores were correlated with clinical disability and preserved biologically meaningful deviations in pwMS, supporting their application in differentiating pathological aging patterns. These findings highlight the potential value of cross-site qMRI harmonization for both normative age modeling and clinical applications in disease-related contexts.

## 1 Introduction

Quantitative MRI (qMRI) has become an increasingly powerful tool for quantifying alterations in brain tissue properties across the lifespan (Filo et al., 2019; Yeatman et al., 2014). qMRI techniques can be applied to measure brain microstructural changes in both grey matter (GM) and white matter (WM) tissues that are not detectable by conventional MRI. Based on qMRI techniques, studies can explore normal physiological development patterns and specific disease-related pathological changes across the lifespan, thereby providing neuroimaging support for the brain’s neurophysiological basis.

Previous studies have presented age-related changes in brain microstructure during adolescence (Norbom et al., 2021), early adulthood (Carey et al., 2018), and late adulthood (Lorio et al., 2014) following logarithmic or exponential decay functions. The patterns show rapid changes in early life followed by slower, continuous alterations across different brain structures (Slater et al., 2019). These findings reflect the prevailing understanding that qMRI mapping metrics show pronounced biological effects. Thus, any study aiming to quantify pathological effects must account for confounding factors, such as age and sex. However, current normative research has often been limited to single-site datasets, which, although maintaining consistency in data sources, restricts generalizability and applicability across different imaging protocols (Leutritz et al., 2020). Although qMRI provides quantitative metrics that should theoretically be comparable across MRI protocols (Weiskopf et al., 2013), in practice, these measures are sensitive to the specific MRI acquisition and post-processing parameters, particularly when assessing subtle variations in relaxation values (A G Teixeira et al., 2019, 2020; Karakuzu et al., 2022). In turn, many existing qMRI studies lack broad age spectrum coverage and are conducted in small cohorts (Keuken et al., 2017; Weiskopf et al., 2013), typically having lower cross-center consistency compared to large-scale, multicenter morphological studies based on conventional MRI data. Furthermore, the cross-center consistency in the morphological studies can be further improved by established harmonization strategies (Fortin et al., 2018).

To mitigate the problem of lacking harmonization methods for qMRI, some studies have proposed standardized multiparameter mapping protocols to control for site effects (Weiskopf et al., 2013). However, these approaches do not apply to conventional retrospective analysis across multiple centers with completed acquisitions. Deep learning approaches have also been proposed for diffusion signal harmonization without requiring additional preprocessing, but their effectiveness often depends on paired multi-scanner datasets from the same subjects, which are challenging to acquire (Feng et al., 2021; Koppers et al., 2019). Moreover, their generalization ability is limited, as performance tends to degrade when applied to acquisition protocols or scanner configurations that deviate from those seen during training. In parallel, some approaches have integrated shallow machine-learning architectures, combining prior knowledge and probabilistic reasoning. These methods show advantages of flexible modeling capacity while enhancing statistical frameworks to improve interpretability and robustness, thereby mitigating the generalization limitations inherent to purely data-driven models. For example, Combat is a commonly used and widely employed harmonization image processing toolbox, typically based on empirical Bayes statistics (EBS), which can be effectively applied in post-hoc harmonization (Fortin et al., 2017). However, Combat has certain limitations, including sensitivity to assumptions of homogeneous variability across batches and the potential loss of site-specific nuances (Johnson et al., 2007). Another option based on the Hierarchical Bayesian Regression (HBR) framework provides a method that accounts for hierarchical data structures, site-specific variations, and heterogeneous variance across sites and covariates (Kia et al., 2020). HBR is expected to effectively address the challenges of multicenter site variance and protocol discrepancies. However, HBR methods present challenges, including increased computational complexity and a requirement for large datasets (de Boer et al., 2024). So far, these methods have been applied to post-hoc harmonization of structural and diffusion MRI data. However, they have not been used on qMRI mapping metrics, and their impact on qMRI analyses remains an open question (Villalón-Reina et al., 2022).

Among various quantitative mapping metrics, R_1_ (longitudinal relaxation rate, R_1_ = 1/T_1_) is one of the most used and well-established measurements. R_1_ is frequently employed to quantify the myelination process non-specifically, as it is primarily influenced by water content, tissue density, the size of the compartments, and exchange rate between intracellular and extracellular free water pools and macromolecular pools, while exhibiting low sensitivity to iron content (Van Gelderen et al., 2016; Wang et al., 2020). One increasingly popular method for R_1_ (or T_1_) mapping is Magnetization Prepared 2 Rapid Acquisition Gradient Echoes (MP2RAGE) (Marques et al., 2010), which enables the achievement of high isotropic resolution. Due to the stability of structural imaging and the relatively well-established post-processing procedures, data acquired from different scanners and centers tend to show minimal variation when using a matched protocol, thereby enhancing the feasibility of combining datasets from various studies. In this context, for MRI studies investigating brain development and lifespan changes, harmonization over multi-site data allows for a broader age range and a larger dataset size, thereby improving statistical power and ensuring more reliable results.

Another popular relaxometry-based quantitative metric used in brain imaging is R_2_* (apparent transverse relaxation rate, R_2_* = 1/T_2_*). R_2_* is sensitive to both macroscopic and mesoscopic local magnetic field inhomogeneities, primarily due to iron deposits, but also reflecting myelin concentration and fiber bundle orientation (Bagnato et al., 2018; Marques et al., 2017). R_2_* is, therefore, used to quantify myelin and iron content indirectly (Barbosa et al., 2015). In this context, a more fundamental question for multicenter data harmonization is whether it is feasible to unify metrics derived from different protocols, particularly those protocols with varying resolutions, which lead to the derived R_2_* being differently sensitive to macroscopic field inhomogeneities. While standardized multicenter protocols represent an ideal solution for ensuring data consistency, they are often not applicable to retrospective studies (Weiskopf et al., 2013). With recent advances in data sharing and large-scale collaborative initiatives facilitating the pooling of multi-site qMRI datasets, efforts to develop robust post-hoc harmonization strategies are critical for accurate data integration, increasing sample sizes, and ensuring more reliable and generalizable findings.

To address the current knowledge gaps, this study aimed to: (i) Evaluate and compare the impact on normative age models obtained using different harmonization approaches when applied to metrics acquired with similar protocol settings and the same resolution, as well as to metrics obtained with unmatched protocol parameters and different resolutions; (ii) Assess the effectiveness of qMRI harmonization strategies on unifying cohorts with fully and partially covered age ranges, and assess the resulting variance in the fitting age-related models; and (iii) Validate the clinical application of qMRI harmonization methods in identifying pathological deviations from normative distribution.

## 2 Methods

### 2.1 Data preparation

This multicenter study included 536 healthy participants across three sites (Jansen et al., 2024; Menks et al., 2022; University Hospital, Basel, Switzerland, 2024). Healthy controls (HCs) were defined as individuals with no known neurological disorders, major systemic illnesses, significant psychiatric conditions, or a history of major surgeries that affected the brain or central nervous system. Due to incompatibility with the image processing pipeline, data from 6 participants were excluded, resulting in a final sample size of 530.

An additional cohort with 101 people with multiple sclerosis (pwMS) from the same center as one of the HC datasets (University Hospital, Basel, Switzerland, 2024) was also included (Clinical Trial number: NCT05177523). Inclusion criteria for patients were as follows: (1) diagnosis according to the 2017 revisions of the McDonald criteria; (2) availability of at least one brain MRI scan, including 1-mm isotropic MP2RAGE and FLuid-Attenuated Inversion Recovery (FLAIR); and (3) availability of at least one annual Expanded Disability Status Scale (EDSS) assessment. MRI scans with insufficient image quality were excluded, resulting in a final sample size of 98. Ethical approval was obtained from the local ethics committee, and all participants provided written informed consent before enrollment.

The cohorts’ demographic characteristics were reported in Table 1.

**Table 1.** Participant demographics.

	Site 1	Site 2		Site 3	Total
Description	Controls	Controls	Patients	Controls	Controls
Participant, n	293	101	98	136	530
R_1_	293	101	98	136	530
R_2_*	287	101	98	-	388
Female/Male, %	53.9/46.1	55.4/44.6	60.2/39.8	65.4/34.6	57.2/42.8
Mean (SD) age, years	50.9 (17.1)	37.7 (12.9)	40.2 (14.3)	17.4 (5.1)	39.8 (19.9)
Age range, years	18–79	18–68	18–75	8–25	8–79
Group Distribution, n					
<20 years	6	2	3	81	89
20–39 years	78	63	50	55	196
40–59 years	99	30	30	0	129
60–79 years	110	6	15	0	116
Phenotypes RRMS/PPMS/SPMS, %	/	/	72.4/8.2/19.4	/	/
Mean (SD) Disease Duration, years	/	/	7.3 (8.6)	/	/
Median (IQR) EDSS	/	/	2.0 (1.5, 4.0)	/	/

SD: standard deviation, Interquartile range from 25% to 75%, RRMS: Relapsing-Remitting Multiple Sclerosis, SPMS: Secondary Progressive Multiple Sclerosis, PPMS: Primary Progressive Multiple Sclerosis, IQR: the interquartile range, EDSS: Expanded Disability Status Scale.

### 2.2 Image acquisition

The imaging protocols included MP2RAGE (Marques et al., 2010) and multi-shell diffusion imaging at all sites, as well as Multi-Echo Gradient Recalled Echo (ME-GRE) sequences (Y. Wang & Liu, 2015) at Sites 1 and 2. Images were acquired using a 3T whole-body MRI system MAGNETOM Prisma (Siemens Healthcare, Erlangen, Germany) for Sites 1 and 2 and MAGNETOM Skyra (Siemens Healthcare, Erlangen, Germany) for Site 3. Acquisition parameters for each site were detailed in Table 2. Please note that Site 3 acquired data from a younger population and used compressed sensing acceleration for the MP2RAGE acquisition to reduce acquisition time, thereby minimizing the likelihood of movement artifacts in this relatively younger population (aged 8–25 years old; Table 1) (Lustig et al., 2007; Mussard et al., 2020). In this scenario, it is conceivable that despite accounting for the protocol changes for R_1_ calculation, differences exist in the effective resolution (due to the different point spread functions induced by both the acquisition and the reconstruction).

**Table 2.** MRI acquisition parameters.

Sequence	Parameter	Site 1	Site 2	Site 3
MP2RAGE-T_1_	Resolution (mm^3^)	1.0 × 1.0 × 1.0	1.0 × 1.0 × 1.0	1.0 × 1.0 × 1.0
	S/P	176	176	224
	TR/TI_1_, TI_2_ (ms)	6000/700/2400	5000/700/2500	5000/700/2500
	FA (°)	6, 6	4, 5	4, 5
	AT (min)	7:32	8:20	4:00
ME-GRE-T_2_*	Resolution (mm^3^)	0.8 × 0.8 × 0.8	0.75 × 0.75 × 3.0	-
	TR/TE1/ΔTE (ms)/nE	44/6.14/4/9	49/6.69/4.06/10	-
	AT (min)	9.25	4.42	-
DWI	Resolution (mm^3^)	1.8 × 1.8 × 1.8	1.8 × 1.8 × 1.8	1.8 × 1.8 × 1.8
	B values	0/1250/2500	0/700/1000/2000/3000	0/925/1850
	Orientation	11/86/85	12/6/20/45/66	6/40/40
	AT (min)	9:39	15:37	4:53

MP2RAGE: Magnetization Prepared 2 Rapid Acquisition Gradient Echoes, ME-GRE: Multi-Echo Gradient Recalled Echo, DWI: diffusion-weighted imaging, S/P: Slice/partitions, TR: Repetition Time, TI_1_: Inversion Time 1, TI_2_: Inversion Time 2, TE1: Echo Time 1, ΔTE: Delta Echo Time, nE: number of Echo; FA: Flip Angle(s), AT: Acquisition Time.

### 2.3 MRI preprocessing and computation of qMRI maps

#### 2.3.1 Data preprocessing

Preprocessing steps were conducted for the MP2RAGE, ME-GRE, and diffusion-weighted images (DWIs). Specifically, MP2RAGE sequences were preprocessed using in-house scripts to generate R_1_ maps (R_1_ = 1/T_1_) while ME-GRE sequences were preprocessed using the SEPIA toolbox (version 1.2.2.4) (Chan & Marques, 2021) to generate R_2_* (R_2_* = 1/T_2_*) maps. Additionally, DWIs were preprocessed using QSIprep (version 0.18.0) (Cieslak et al., 2021) to derive the WM bundles.

The entire data processing pipeline has been previously extensively described (Jansen et al., 2024; Shams et al., 2019), and the updated details can also be found in the Supplementary Materials (S.1).

#### 2.3.2 Cortical parcellation and WM bundle identification

Segmentation of cortical grey matter (cGM) was performed using FreeSurfer (version 6.0) (Fischl, 2012) on MP2RAGE data, following the standard recon-all pipeline. A refined PALS-B12 Brodmann atlas was used to parcellate the cortex into 41 Brodmann areas (BAs) (Van Essen, 2005).

To obtain bundle information, a pyAFQ (Kruper et al., 2021) pipeline of QSIprep was applied, allowing the identification of eighteen bundles including Callosum Forceps Minor (FA), Callosum Forceps Major (FP), and the following bilateral tracts: Arcuate (ARC), Posterior Arcuate Fasciculus (pARC), Thalamic Radiation (ATR), Cingulum Cingulate (CGC), Corticospinal (CST), Inferior Fronto-Occipital Fasciculus (IFO), Inferior Longitudinal Fasciculus (ILF), and Superior Longitudinal Fasciculus (SLF).

#### 2.3.3 Quantitative mapping across brain structures

Quantitative surface maps were generated by projecting qMRI values onto brain surfaces derived from FreeSurfer. Measurements were extracted from the middle layers of cGM (0.5 mm between the white and pial surfaces) and sWM (-0.5 mm below the WM boundary) to mitigate partial volume effects. This approach ensured tissue-specific quantification not only for cGM but also for sWM, enabling a more comprehensive assessment of microstructural changes across both GM and adjacent WM compartments based on cortical parcellation. Additionally, sWM regions were labeled and further reported based on adjacent BAs, due to shared cortical parcellation with cGM regions. Average qMRI measurements (R_1_, R_2_*) were computed for each BA, averaging across both hemispheres.

For WM bundles-based analysis, only the central 70% of the central fiber tract was analyzed to minimize partial volume effects. qMRI measurements were extracted using the SCILPY toolbox (Renauld et al., 2026).

### 2.4 Harmonization of qMRI values across datasets

#### 2.4.1 Quantifying site variability

To quantify site-related variability before harmonization, we calculated the intra-class correlation coefficients (ICC) for each brain region, a widely used reliability assessment metric in neuroimaging studies to quantify batch variance and measurement reproducibility across multiple MRI datasets (G. Chen et al., 2018; Gebre et al., 2023). The ICC quantifies the proportion of total variance attributable to site effects, with higher values indicating more substantial site-related variability. To distinguish biologically meaningful differences from site-induced variability, we computed two types of ICCs per region:

- unadjusted ICCs, derived from models that include site as a random intercept,
$${\text{qMRI}}_{\text{ij}}=\mu +{\text{b}}_{\text{j}}+\varepsilon_{\text{ij}}$$ (1)
  where qMRI_ij_ is the regional qMRI metrics (R_1_ or R_2_* values) for subject i in site j, μ is the overall mean, b_j_ is the random effect for site j, ε_ij_ is the residual error. Both random effects and residuals were assumed to follow Gaussian distributions with variances σ_b_^2^ (capturing between-site variability) and σ_ε_^2^, respectively;
- adjusted ICCs, from models that also included age and sex as fixed effects
$${\text{qMRI}}_{\text{ij}}={\text{β}}_{0}+{\text{β}}_{\text{1}}\cdot {\text{Age}}_{\text{ij}}+{\text{β}}_{\text{2}}\cdot {\text{Age}}_{\text{ij}}^{\;\;\;\;\text{2}}+{\text{β}}_{\text{3}}\cdot {\text{Sex}}_{\text{ij}}+{\text{b}}_{\text{j}}+{\text{ε}}_{\text{ij}}$$ (2)
  where β_0_ is the intercept, β_1_, β_2_, and β_3_ are fixed effect coefficients for Age, Age^2^, and Sex, respectively;

In both cases, ICCs were defined as the ratio of site-related variance to total variance as

$$\text{ICC}=\sigma_{\text{b}}^{\;\;2}/(\sigma_{\text{b}}^{\;\;\;2}+\sigma_{\varepsilon }^{\;\;\;2})$$ (3)

This allowed for the assessment of site-related variance both before and after accounting for key demographic covariates.

ICC analysis was performed separately for cGM, sWM, and WM bundles to quantify regional site-related variance while accounting for age and sex. To provide an intuitive visualization of the overall site structure in the data, we additionally performed principal component analysis (PCA) on the raw regional qMRI values. PCA was used as an exploratory, unsupervised tool to illustrate clustering patterns across sites, complementing the quantitative ICC results and providing a visual representation of site effects.

#### 2.4.2 Harmonization models

Harmonization of qMRI values across datasets was performed using Combat-GAM, an extension of neuroCombat that incorporates GAM (Pomponio et al., 2020). This method was employed to adjust for batch effects commonly found in multi-site studies, such as differences in scanner type, protocol parameters, and environmental conditions. Importantly, Combat-GAM models and corrects for both batch effects and potential nonlinear covariate effects (e.g., nonlinear relationships with age), offering a more flexible approach than traditional linear models (Pomponio et al., 2020). The empirical Bayes model was applied with age and sex included as covariates to ensure that the harmonization process accounted for demographic factors while preserving meaningful biological variation. The Site 1 dataset was selected as the reference because it covered the widest age range. As a benchmark, the ComBat model with linear covariates (age and sex) was also analysed for completeness, but was not considered a primary harmonization model.

We further employed an HBR framework using a B-spline model, as implemented in the PCN toolkit (de Boer et al., 2024), to estimate the mean and variance of each regional qMRI measure as a function of age. The HBR framework enabled us to compute individualized Z-scores relative to age-expected distributions, providing an alternative harmonization approach for comparison with the Combat-GAM results. Site 1 data served as the reference dataset, while data from other sites (changing datasets) were projected into the reference space of Site 1 by back-transforming Z-scores, effectively correcting for site effects and enabling pooling across sites. In this step, stratified five-fold cross-validation was used to ensure consistency in both site data and sex ratios across folds after pooling data from different sites. In each fold, 80% of the data were used for model training (20% for validation). The root mean square error was employed to identify the best model among all folds. Harmonized data in the changing datasets were then obtained by inversely transforming the corresponding Z-scores to the reference dataset model. Details can be found in the Supplementary Materials (S.2).

#### 2.4.3 Residual site effect assessment

η^2^ values were derived from ANOVA models, both before and after harmonization, to quantify the residual variance explained by site for qMRI metrics in each brain region. η^2^ was calculated as the proportion of variance explained by site in linear models that included site as a fixed effect alongside age and sex, providing a measure of effect size (values approaching zero indicate minimal residual site effects). This post-harmonization assessment was used to evaluate the effectiveness of harmonization in reducing site-related variability across brain structures.

### 2.5 Harmonization impact on modeling age-related effects

#### 2.5.1 Normative age modeling and peak age estimation

Age-related effects were modeled using second-order polynomial regression with age, age^2^, and sex as predictors to capture potential nonlinearities (Eq. 4). The necessity of including the linear term (age) was assessed using likelihood ratio tests to ensure model parsimony and biological plausibility. The turning points, defined as the peak ages of the quadratic curves, were estimated using polynomial regression, with their standard errors (SEs) derived by error propagation (Eqs. 5–6]. To further quantify the uncertainty in the peak age estimation of each quadratic model, 10,000 bootstrap resamples were used to compute the 95% confidence interval (CI).

$${\text{qMRI}}_{\text{ROI}}\;=\;\beta_{0\text{ROI}}\;+\;\beta_{1\text{ROI}}\;\text{*}\;\text{Age}\;+\;\beta_{2\text{ROI}}\;\text{*}\;{\text{Age}}^{2}\;+\;\beta_{3\text{ROI}}\;\text{*}\;\text{Sex}$$ (4)

$${\text{Age}}_{\text{peak}}=-\beta_{1\text{ROI}}\;/\;\left(2\;\text{*}\;\beta_{2\text{ROI}}\right)$$ (5)

$$\text{SEpeak }=\sqrt{{\left(\frac{{\text{SE}}_{\beta_{1\text{ROI}}}}{2\;\;\times \;\;\beta_{2\text{ROI}}}\right)}^{2}+\;\;{\left(\frac{\beta_{1\text{ROI}}\;\;\times \;\;{\text{SE}}_{\beta_{2\text{ROI}}}}{2\;\;\times \;\;\beta_{2\text{ROI}}^{2}}\right)}^{2}}$$ (6)

This model included age, age^2^, and sex as covariates. β_1ROI_ and β_2ROI_ are the coefficients of the linear and quadratic terms, respectively. A likelihood-ratio test (LRT) was used to confirm the significance of the quadratic term. The peak age of the qMRI curve and its Standard Error (SE) were calculated from the quadratic regression equation and using error propagation (Eq. 5). When the quadratic coefficient was non-zero, the peak age was determined at the maximum or minimum of the quadratic curve. If the quadratic coefficient was not significant, the peak age was not defined.

#### 2.5.2 Peak ages correlation analysis

To assess the impact of harmonization on normative age modeling, we conducted a peak age correlation analysis comparing estimates derived from raw and harmonized data and evaluated which method provides the most biologically sound results. Peak ages obtained from raw data and those estimated after applying two different harmonization methods (GAM and B-spline models) were compared using pairwise Spearman correlation analyses, with unstable or unreliable peak age estimates controlled for using the interquartile range (IQR)-based filtering approach that excluded regions with bootstrap standard errors beyond 1.5 times the IQR.

Given that R_2_* data were acquired from two sites with comparable age ranges but differing spatial resolutions, Z-score normalization was applied within each site before modeling age-related effects. In this step, R_2_* values were standardized by subtracting the site-specific mean and dividing by the site-specific standard deviation. This approach was applied to mitigate potential scale and distribution differences arising from resolution-related and site-specific variability, which could otherwise confound modeling based on raw values. In contrast, in R_1_, where data were collected from three sites with the same resolution and matched protocols, but with partially overlapping and non-identical age ranges, raw values were used for modeling to directly examine the feasibility of combining cohorts with different age distributions.

#### 2.5.3 Root mean square error estimation

To further evaluate model fit and quantify the impact of harmonization on age modeling, Root Mean Square Error (RMSE) was computed before and after harmonization for each region. For each region, an ordinary least-squares regression model of the qMRI metric was fitted as a function of age, age^2^, and sex, and RMSE was defined as the square root of the mean squared residuals between observed and predicted values. This metric provides a direct measure of model performance by capturing the dispersion of individual data points around the estimated age trajectories.

#### 2.5.4 Validating the application of qMRI harmonization in pwMS

Given that the HBR-based B-spline model enables direct estimation of individual-level Z-scores while accounting for site and demographic covariates (Marquand, 2026), we used this approach to assess patient-specific deviations from normative distributions. In contrast, ComBat-GAM produces harmonized individual qMRI values but does not include an additional normative modeling step (Pomponio, 2025) and therefore does not directly provide subject-level normative deviation scores (Z-scores). Based on the model trained with controls from Sites 1 and 2, we estimated Z-scores using HBR-based B-spline models for pwMS from Site 2. Site 3 was excluded from modeling due to age mismatch with the MS cohort. These Z-scores were then compared between pwMS and HCs to evaluate pathological effects.

Cohen’s d was computed to assess group-level differences in R_1_ and R_2_* metrics between MS and HC groups across regions. To account for potential confounding, multivariable linear regression (MLR) models were first fitted with age and sex as covariates, and residuals were used to calculate effect sizes. In addition to the harmonized comparisons between pwMS and HCs across all sites, we also performed comparison analyses using raw data within the same site (pwMS and HCs both from Site 2) and across sites (pwMS from Site 2 and HCs from Site 1). Within-site and cross-site comparison analyses provided direct reference points for evaluating the impact of harmonization on disease-related variability.

To further assess the clinical relevance of harmonized qMRI measures, we evaluated the associations between patients’ brain regional Z-scores and clinical disability, as measured by EDSS scores, using MLR models adjusted for sex, phenotype, and disease duration, with false discovery rate (FDR) correction restricted to brain regions that previously showed significant group-level deviations from controls.

$$\begin{array}{l} {\text{EDSS}}_{i}\;=\;\beta_{0}\;+\;\beta_{1}\cdot \;{\text{Z}}_{\text{ij}}+\;\beta_{2}\cdot \;{\text{Diagnosis}}_{i}+\;\beta_{3}\cdot \;\text{Disease}\_{\text{Duration}}_{i}\\ \;\;\;\;\;\;\;\;\;\;\;\;\;\;\;\;\;\;\;\;+\;\beta_{4}\cdot \;{\text{Sex}}_{i}+\;\varepsilon_{i} \end{array}$$ (7)

Z_ij_ represents the Z-score of subject i in region j. Diagnosis_i_ is the clinical phenotype (e.g., Relapsing-Remitting MS, Secondary Progressive MS), Disease_Duration_i_ is the duration of disease in years, and EDSS_i_ is the Expanded Disability Status Scale score for subject i.

All statistical analyses were performed using R (version 4.2.3) (R Core Team, 2013) with a significance threshold set at 0.05. FDR was applied to multiple comparisons.

## 3 Results

### 3.1 Effects of harmonization on R_1_ across cohorts with matched protocols

#### 3.1.1 Batch effect evaluation

At this step, ICC values were computed across healthy cohorts (Sites 1, 2, and 3) with matched R_1_ protocols at the same resolution but with partially overlapping age ranges. ICC was computed before and after adjusting for age and sex. The mean corrected ICC after adjusting for age and sex across all regions was 0.71 ± 0.10 (uncorrected: 0.38 ± 0.18) for cGM, 0.28 ± 0.13 (uncorrected: 0.19 ± 0.13) for sWM, and 0.14 ± 0.08 (uncorrected: 0.08 ± 0.09) for WM bundles. Full results were detailed in Supplementary Materials (S.3.1).

**Fig. 1.**
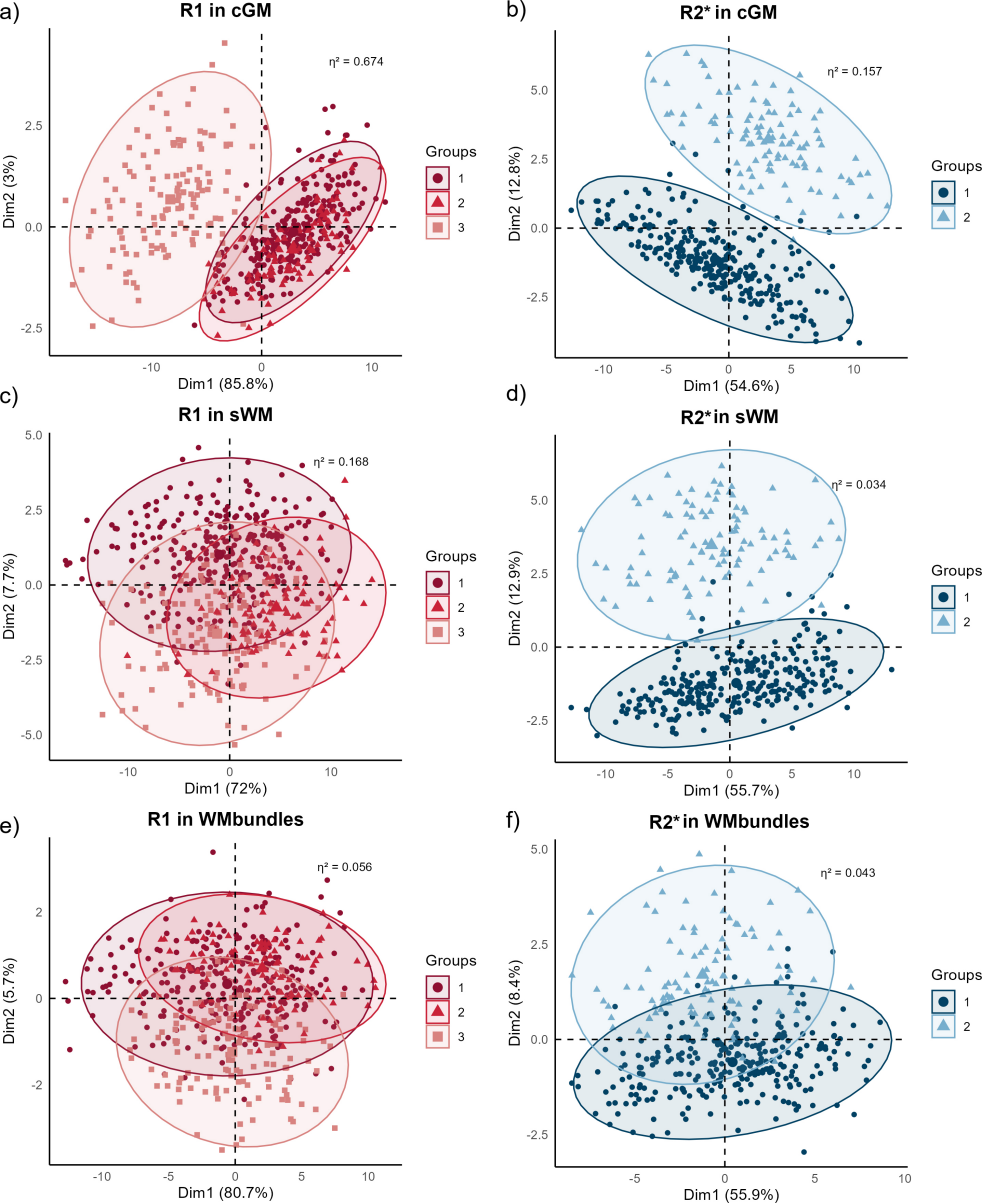
Principal component analysis of site-dependent variance in R_1_ and R_2_*. The plot presents principal component analysis (PCA) illustrating site-related variance, showing an apparent clustering of participants by site, especially along the first principal component. Each point represents one participant. Panels (a), (c), and (e) show results of R_1_, while panels (b), (d), and (f) show results of R_2_*. Data from different sites are presented using different labels in *Group* label. Clustering is more evident for R_1_ in cortical grey matter in (a), whereas sWM and WM bundles in (c) and (e), separately, show less separation by site. Ellipses represent the 95% confidence intervals for each site, and η^2^ indicates the proportion of variance explained by the site effect. Note that, as a linear method, PCA does not account for possible nonlinear effects of age or sex. Thus, the observed site-related variance includes demographic differences across sites. cGM: cortical grey matter, sWM: superficial white matter, WM: white matter. PCA: principal component analysis.

PCA analysis illustrated the contribution of site effects to R_1_ variance, with details presented in Figure 1a, c, and e.

#### 3.1.2 Reduction of site-related variance in R_1_ after harmonization

After harmonization, the average η^2^ for batch effects across regions decreased from 0.11 ± 0.09 in the raw data to <0.01 after both GAM and B-spline harmonization, indicating minimal residual variance attributable to site differences after adjusting for age, age^2^, and Sex. In most areas, site-related variance was nearly eliminated after both GAM and B-spline harmonization, leading to model singularity and a non-estimable corrected ICC. The site-level random effect collapsed to the boundary of the parameter space after covariate adjustment, supporting the effectiveness of GAM and B-spline harmonization in minimizing site effects. Additionally, after standard ComBat harmonization, the average η^2^ decreased slightly compared to the raw data (0.07 ± 0.04), while the mean-corrected ICC slightly increased (0.25 ± 0.11) compared to the raw data (0.24 ± 0.19), as detailed in the Supplementary Materials (S.3.1 and S.3.2), suggesting that the standard ComBat model was less effective than the GAM method in controlling site effects and nonlinear biological variances.

**Fig. 2.**
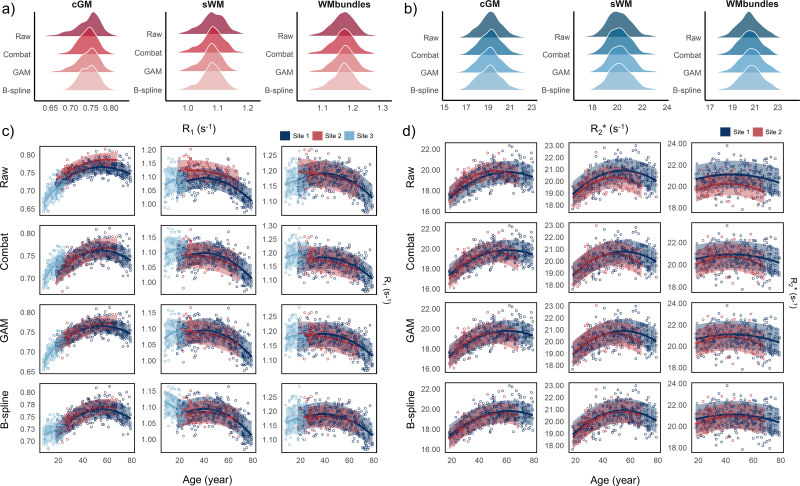
Effects of harmonization on R_1_ and R_2_* across sites. (a, b) Raw and harmonized distributions of R_1_ and R_2_* values measured in Brodmann area 4 and ARCR, representing cortical and white-matter regions, respectively. (c, d) Quadratic age-fitted curves of the original and harmonized data within each site, with colors indicating different centers. In R_1_ (c), the standard Combat model shows a vertical offset for site 3, reflecting residual site effects due to partially non-overlapping age ranges across cohorts, whereas in R_2_* (d), where data were acquired from sites with largely overlapping ages, it performs comparably to the GAM model. The B-spline and GAM models both reduce site-related differences, yielding more consistent age-fitting trends. However, their fitted patterns differ. The B-spline model is less conservative, allowing non-quadratic behavior at younger ages in certain regions, as illustrated by the example region. In contrast, the GAM model retains a smoother, quadratic-like trend. cGM: cortical gray matter; sWM: superficial white matter; WM: white matter; GAM: generalized additive model.

The results of the example regions were presented in Figure 2 for R_1_, with the effects of harmonization evident in the cGM of Figure 2a. After harmonization, the tail was shortened in sWM, resulting in a more normally distributed pattern. It is also clear that the introduction of harmonizations (GAM and B-spline) resulted in a more coherent modeling of age-related effects across the three selected regions (Fig. 2c), whereas standard ComBat harmonization introduced a systematic shift in the data distribution along the fitted age trajectory. It is worth noting that, for Site 3, the two harmonization methods yielded different results in the sWM, indicating regional discrepancies in harmonization performance and normative age modeling between the GAM and B-spline models. To evaluate the impact of harmonization in all other regions beyond BA 4 (cGM and sWM) and the ARCR bundle, the regional η^2^ and ICC were presented in Supplementary Materials (S.3.2). Additionally, the fitting age models’ results were shown in Supplementary Materials (S.3.3) PCA analyses illustrating site-related variance in the presence of biological variability after harmonization (particularly in longitudinal relaxation rate, where a younger cohort’s data from Site 3 is present) are additionally presented in Supplementary Materials (S.4, Fig. S1). These findings suggest that both methods (GAM and B-spline) significantly reduced site effects that were previously confounded with biological variation.

#### 3.1.3 Harmonization impact analysis on R_1_ age modeling

Brain regions with unstable peak age estimates were excluded using IQR filtering to ensure reliable comparisons (regions with bootstrap standard errors exceeding 1.5 times the IQR were removed). After filtering, group-level peak ages were reported as mean, standard error (SE), and percentage of ROIs retained after filtering (retained ROIs divided by total ROIs times 100), derived from raw and harmonized R_1_ data were compared across brain structures (Details of the peak age results were shown in Supplementary Materials (S.3.3)). In cGM, peak age shifted from 55.62 (0.29) (90%) years (raw) to 57.63 (0.44) (87.5%) and 56.51 (0.33) (87.5%) years after B-spline and GAM harmonization, respectively; in sWM, peak ages shifted from 39.94 (0.77) (95%) years (raw) to 38.72 (1.19) (92.5%) and 40.44 (0.82) (90%) years; and in WM bundles, from 37.10 (0.99) (90.5%) years (raw) to 35.69 (0.83) (85.7%) and 34.63 (0.70) (81%) years. These filtered estimates yield robust, stable peak-age assessments after excluding brain regions with high uncertainty. Moreover, the differences between the raw data and harmonized estimates were minimal (mean differences of 0.7 years for GAM and 1.1 years for HBR; maximum difference of 4.0 years in WM bundles for HBR), underscoring the consistency and reliability of the harmonization approaches in modeling age-related effects across cohorts.

**Fig. 3.**
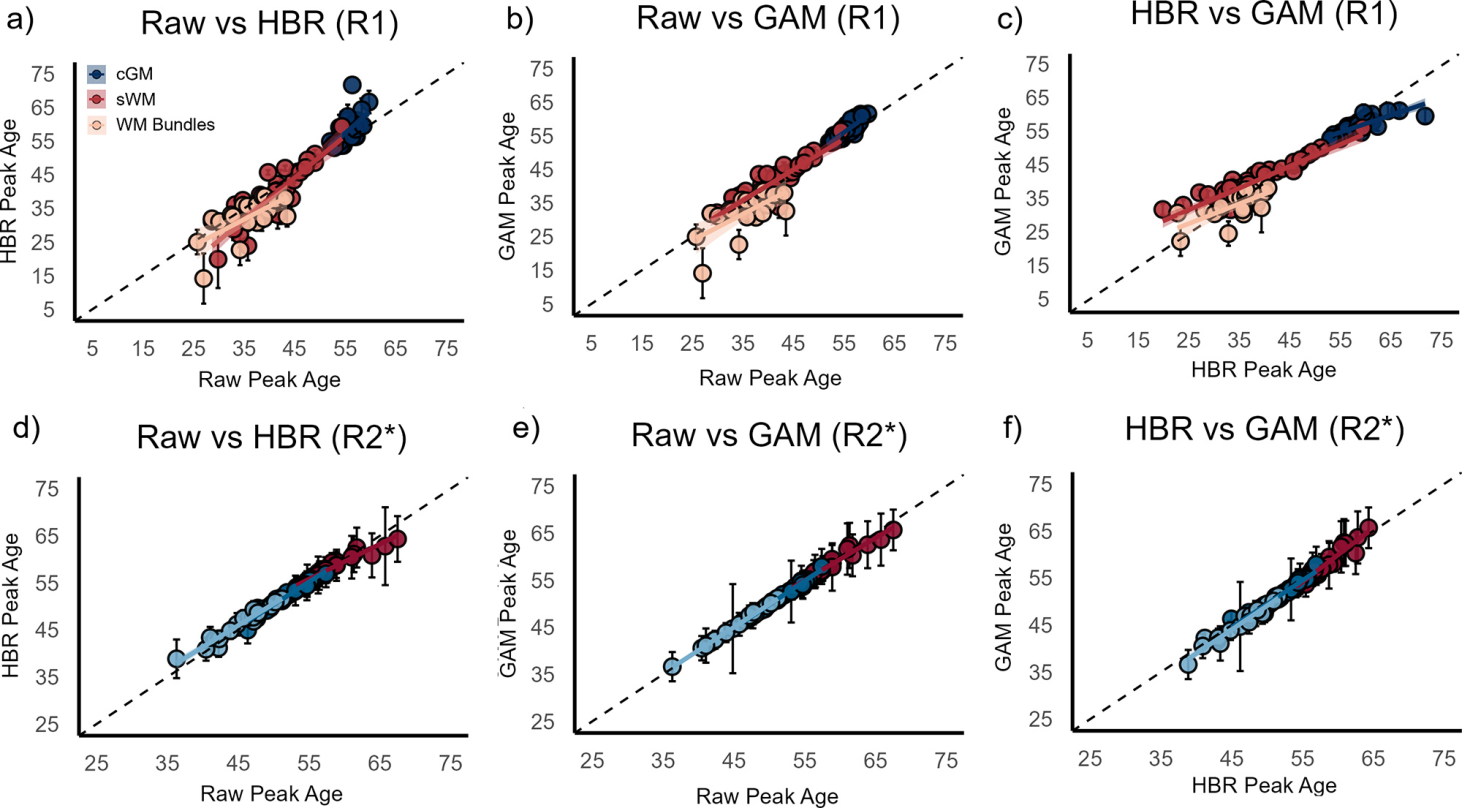
Comparison of peak age estimates from original and harmonized data. Panels (a–c) show the pairwise Spearman correlation analysis results of the peak ages derived from the quadratic regression model of age calculated based on the original R_1_ and the unified R_1_ obtained by the two harmonization methods. Each point represents a brain region, and different colors represent different brain structures. The colored lines represent ordinary least-squares linear regressions with 95% confidence bands for ROI-wise peak ages from the raw and harmonized datasets within each tissue type. Panels (d-f) show the results of R_2_*. R_2_* peak ages are derived from the quadratic regression models of age fitted by the original and harmonized data (both after Z-score normalization). Please note that the peak ages of the R_2_* quadratic regression model of age, calculated based on both the original R_2_* and the harmonized R_2_*.

Pairwise correlation analyses showed that the peak ages derived from quadratic models fitted to either the original or harmonized R_1_ data across cGM, sWM, and WM bundles were significantly correlated (p < 0.01, Fig. 3a–c, with details in Supplementary Materials (S.3.4)).

After harmonization, the average RMSE decreased by 11.4% (IQR: 6.5% to 14.0%) in cGM, 6.0% (IQR: 1.5% to 9.0%) in sWM, and 1.5% (IQR: 0.1% to 2.7%) in WM bundles with the GAM model. Using the B-spline model, the average RMSE decreased by 13.2% (IQR: 9.2% to 16.3%) in cGM, 1.2% (IQR: −4.3% to 5.1%) in sWM, and 1.5% (IQR: 0.1% to 2.7%) in WM bundles. Detailed RMSE values before and after harmonization, as well as the corresponding percentage changes across all regions, were provided in Supplementary Materials (S.3.5).

### 3.2 Effects of harmonization on R_2_* across cohorts with unmatched protocols

#### 3.2.1 Batch effect quantification and correction

Computed ICC before and after adjusting for age and sex, showing batch effects (Sites 1 and 2) in R_2_* across brain structures. The mean-corrected ICC after adjusting for age and sex across regions was 0.38 ± 0.27 (uncorrected: 0.26 ± 0.31) in cGM, 0.23 ± 0.24 (uncorrected: 0.22 ± 0.22) in sWM, and 0.20 ± 0.16 (uncorrected: 0.14 ± 0.18) in WM bundles.

PCA analysis illustrated the contribution of site effects to variance, with details presented in Figure 1b, d, and f.

#### 3.2.2 Reduction of site-related variance in R_2_* after harmonization

Compared to R_1_, the impact in R_2_* was less dramatic. Both GAM and B-spline harmonization effectively minimized batch effects, reducing them from 0.15 ± 0.20 across regions of raw data to nearly 0 (<0.001) after harmonization. In the example region shown in Figure 2b, variations in histogram shapes are visible, especially in WM bundles, where an abnormally sharp Gaussian-like peak is present, indicating intensity clustering in the raw data. In Figure 2d, the harmonized data (HBR and GAM) become more continuous and centralized along the quadratic age trajectory. The separated parabolic trends in raw data approach an overlapping smooth curve. Consequently, ICC could not be estimated in regions with negligible site-related variance, confirming the reduction of site effects. It is also noted that, with similar age distributions in the R_2_* data from both sites, the standard ComBat model produced a reduced mean η^2^ (<0.001) and non-estimable corrected ICC values, showing performance similar to the GAM model (see supplementary analysis results in S.3.2 and S.4, Fig. S1).

#### 3.2.3 Harmonization impact analysis on R_2_* age modeling

R_2_* showed comparable peak age shift trends to those observed for R_1_, group-level peak ages were 57.65 (0.48) (87.5%) years (raw), shifting to 57.97 (0.39) (92.5%) with B-spline and 57.39 (0.46) (90.0%) with GAM in cGM; 51.28 (0.41) (90.0%) years (raw) to 51.57 (0.37) (80.0%) and 51.02 (0.41) (87.5%) in sWM; and 45.08 (0.91) (90.5%) years (raw) to 46.60 (0.75) (90.5%) and 45.29 (0.90) (90.5%) in WM bundles. These results confirmed that harmonization methods had small impacts on age modeling, in line with R_1_ findings. However, it is essential to note that R_2_* analyses were based on two sites sharing a similar age range, which may contribute to the observed consistency after harmonization, unlike R_1_ analyses that combined data across sites with different age ranges.

Pairwise correlation analyses showed that the peak ages derived from quadratic models fitted to either the original or harmonized R_2_* data across cGM, sWM, and WM bundles were significantly correlated (p < 0.05) (see Fig. 3d, e, and f).

After harmonization, the average RMSE decreased by 15.0% (IQR: 1.7–20.8%) in cGM, 7.7% (IQR: 0.1–5.4%) in sWM, and 3.8% (IQR: 2.0–1.1%) in WM bundles with the GAM model. Using the B-spline model, the average RMSE decreased by 17.4% (IQR: 4.5–22.9%) in cGM, 9.8% (IQR: 1.9–7.8%) in sWM, and 3.8% (IQR: 2.0–1.1%) in WM bundles. Regional RMSE values were provided in the Supplementary Materials (S.3.5).

### 3.3 Validate the application of qMRI harmonization in clinical contexts

#### 3.3.1 Assessing pathology-preserved R_1_ and R_2_* differences after harmonization

For R_1_, 10 out of 41 cGM regions showed differences between pwMS and HCs (*P*_FDR_ < 0.05), with higher R_1_ values in controls across all significantly different regions (median Cohen’s d [IQR]: 0.21 [0.03, 0.27]). The largest effect size was observed in BA 46 (Cohen’s d = 0.38). In sWM, all regions showed group differences (*P*_FDR_ < 0.05), with moderate to large effect sizes (0.59 [0.52, 0.63]). The largest effect size was observed in BA 28 (Cohen’s d = 0.91), where R_1_ was consistently higher in the controls. All WM bundles also showed significantly higher R_1_ in controls (*P*_FDR_ < 0.05), with large effect sizes (0.63 [0.60, 0.95]). The largest effect size of the group difference was observed in the Inferior Fronto-Occipital Fasciculus (IFOL) (Cohen’s d = 1.13).

R_2_* values were consistently higher in controls compared to pwMS. Small to moderate group differences were observed in cGM (0.34 [0.26–0.50]), with 31 out of 41 regions showing significant effects (*P*_FDR_ < 0.05). The most considerable difference was observed in the parietal lobe at BA 2 (Cohen’s d = 0.75). In sWM, 38 out of 41 regions showed significant differences, with moderate effect sizes overall (0.54 [0.36–0.59]). The largest effect size of the group difference was observed in the occipital lobe at BA 19 (Cohen’s d = 0.80). All WM bundles exhibited significant R_2_* differences (*P*_FDR_ < 0.05), with moderate to large effect sizes (0.78 [0.59, 0.89]). The largest effect size was observed in IFOL (Cohen’s d = 1.16), consistent with the R_1_ findings in WM bundles.

Details of Cohen’s d test results were presented in Supplementary Materials (S.3.6).

#### 3.3.2 Assessing pathology-preserved R_1_ and R_2_* differences on raw data

Within-site and cross-site analyses using raw data were conducted to examine the correspondence between the harmonized and raw data results. The within-site (Site 2) comparison yielded effect directions for both R_1_ and R_2_* that were consistent with the harmonized results, showing a similar overall pattern of regional significance but generally weaker statistical significance (higher p values and fewer significant ROIs) than the harmonized data. In contrast, the cross-site comparisons exhibited mixed or less consistent patterns. Results from the harmonized, within-site, and cross-site comparison were summarized in Table 3, with detailed regional findings provided in Supplementary Materials (S.3.6).

**Table 3.** Pathology-preserved R_1_ and R_2_* differences comparisons.

		Raw Data, Cross-site (pwMS from site 2, HC from site 1)			Raw Data, Within-site (pwMS, HC both from site 2)			Harmonized Data (across sites)		
Metric	Tissue	ROI (n/N)	Cohen’s d (Med, IQR)	Direction	ROI (n/N)	Cohen’s d (Med, IQR)	Direction	ROI (n/N)	Cohen’s d (Med, IQR)	Direction (*P* trends)
**R_1_**	cGM	26/41	-0.56 [-0.65, 0.10]	HC<MS (19ROIs) HC>MS (7ROIs)	0	-	HC>MS	10/41	0.21 [0.03, 0.27]	HC>MS (↓)
	sWM	26/41	−0.33 [−0.53, 0.32]	HC<MS (7ROIs) HC>MS (19ROIs)	41/41	0.68 [0.62, 0.72]	HC>MS	41/41	0.59 [0.52, 0.63]	HC>MS (↓)
	WM bundles	17/18	0.55 [0.44, 0.86]	HC>MS	18/18	0.76 [0.72, 0.96]	HC>MS	18/18	0.63 [0.60, 0.95]	HC>MS (↓)
R_2_*	cGM	30/41	-0.72 [-1.79, -0.37]	HC<MS (19ROIs) HC>MS (11ROIs)	19/41	0.50 [0.49, 0.62]	HC>MS	31/41	0.34 [0.26, 0.50]	HC>MS (↓)
	sWM	38/41	0.92 [0.36, 1.07]	HC>MS (24ROIs) HC<MS (14ROIs)	34/41	0.64 [0.46, 0.72]	HC>MS	38/41	0.54 [0.36, 0.59]	HC>MS (↓)
	WM bundles	18/18	1.16 [1.08, 1.24]	HC>MS	18/18	0.81 [0.64, 0.93]	HC>MS	18/18	0.78 [0.59, 0.89]	HC>MS (↓)

ROI (n/N): Number (n) and proportion (n/N) of region showing a significant difference. Direction: Group effect direction. Med, IQR: Median and interquartile range. cGM: Cortical gray matter. sWM: Superficial white matter. WM: White matter. HC: Healthy control. MS: Multiple sclerosis. *P* trends: ↓ decreased or ↑ increased *P*_FDR_ after harmonization compared to the Within-site comparison.

#### 3.3.3 Z-score measurement for evaluating pathological aging effect

**Fig. 4.**
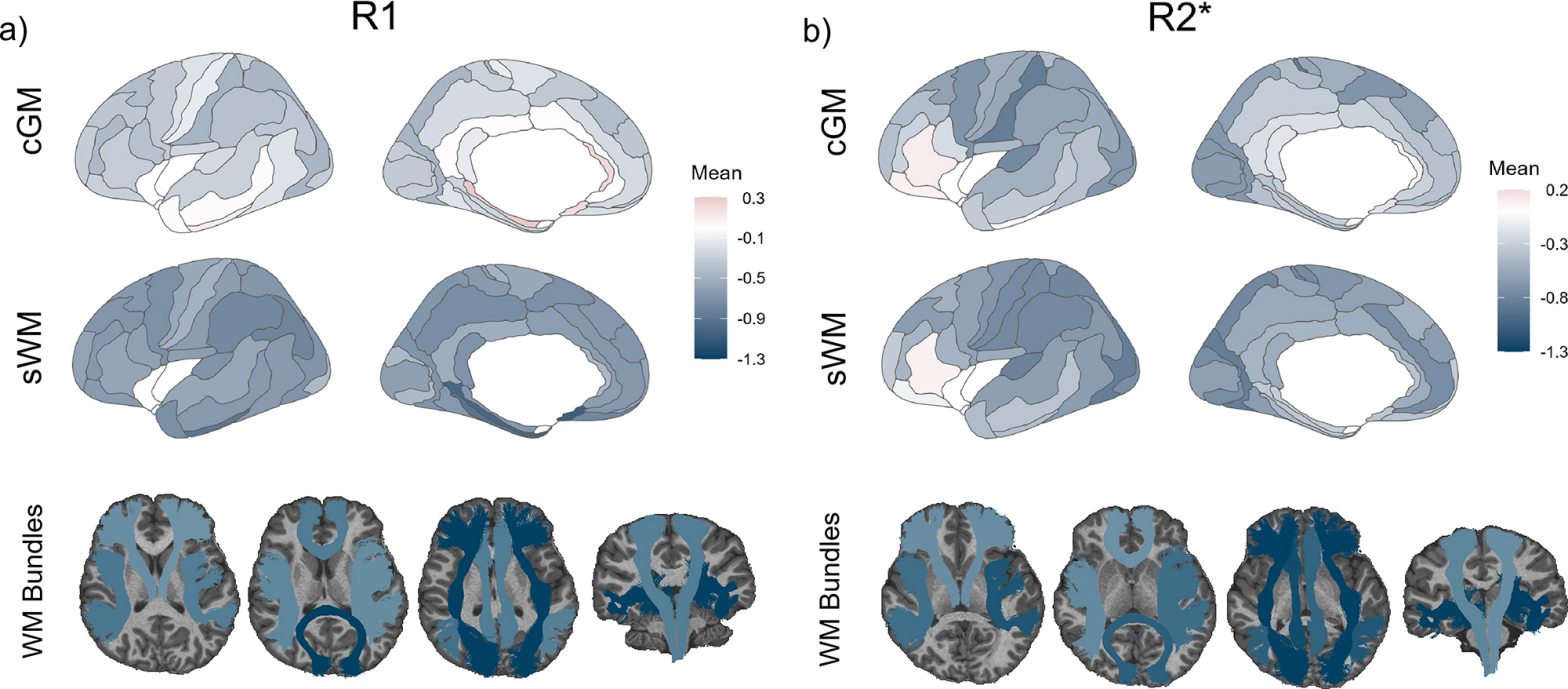
Regional Z-score deviations of qMRI metrics in pwMS. Panels (a) and (b) show the distribution of the mean Z-score values of R_1_ and R_2_* in different brain regions under the cortical grey matter (cGM), superficial white matter (sWM), and white matter (WM) bundles in pwMS, representing the deviation of pwMS from the healthy controls. The color bar reflects the mean Z-score distribution, with warmer (red) colors indicating greater positive deviation and cooler (blue) tones indicating lower values. cGM: cortical grey matter, sWM: superficial white matter, WM: white matter, pwMS: people with multiple sclerosis.

Based on the HBR-based B-spline modeling, the mean R_1_ Z-scores across regions were estimated for the patient population (as shown in Fig. 4a). Reduced average R_1_ Z-scores were observed across most cGM regions in pwMS, except for nine areas. The largest deviation was observed in BA2 (-0.47 ± 1.02). In sWM, Z-scores reduced in pwMS across all regions, with the most substantial deviation in BA25 at subgenual cingulate (-1.06 ± 1.48). Similarly, all WM bundles showed lower Z-scores, with the largest deviation observed in ILFR (-1.29 ± 1.33). This observation is consistent with a decrease in myelination throughout the brain, particularly in WM bundles.

Figure 4b presents a similar analysis of the patient’s average Z-score for R_2_*, where most regions exhibited reduced average values in pwMS compared to controls. The most substantial deviations were observed in BA2 (–0.85 ± 1.06) of the parietal cortex in cGM, BA19 (–0.84 ± 0.94) in the associative visual area of the sWM, and the IFOF (–1.25 ± 1.04) in WM bundles. These results suggest that the primary aging mechanism driving R_2_* in patients is the loss of myelin and an increase in iron.

For completeness, Z-score maps derived from the raw data are additionally provided in Supplementary Materials (S.4, Fig. S2).

#### 3.3.4 Clinical correlation analysis on evaluated pathological aging effect

**Fig. 5.**
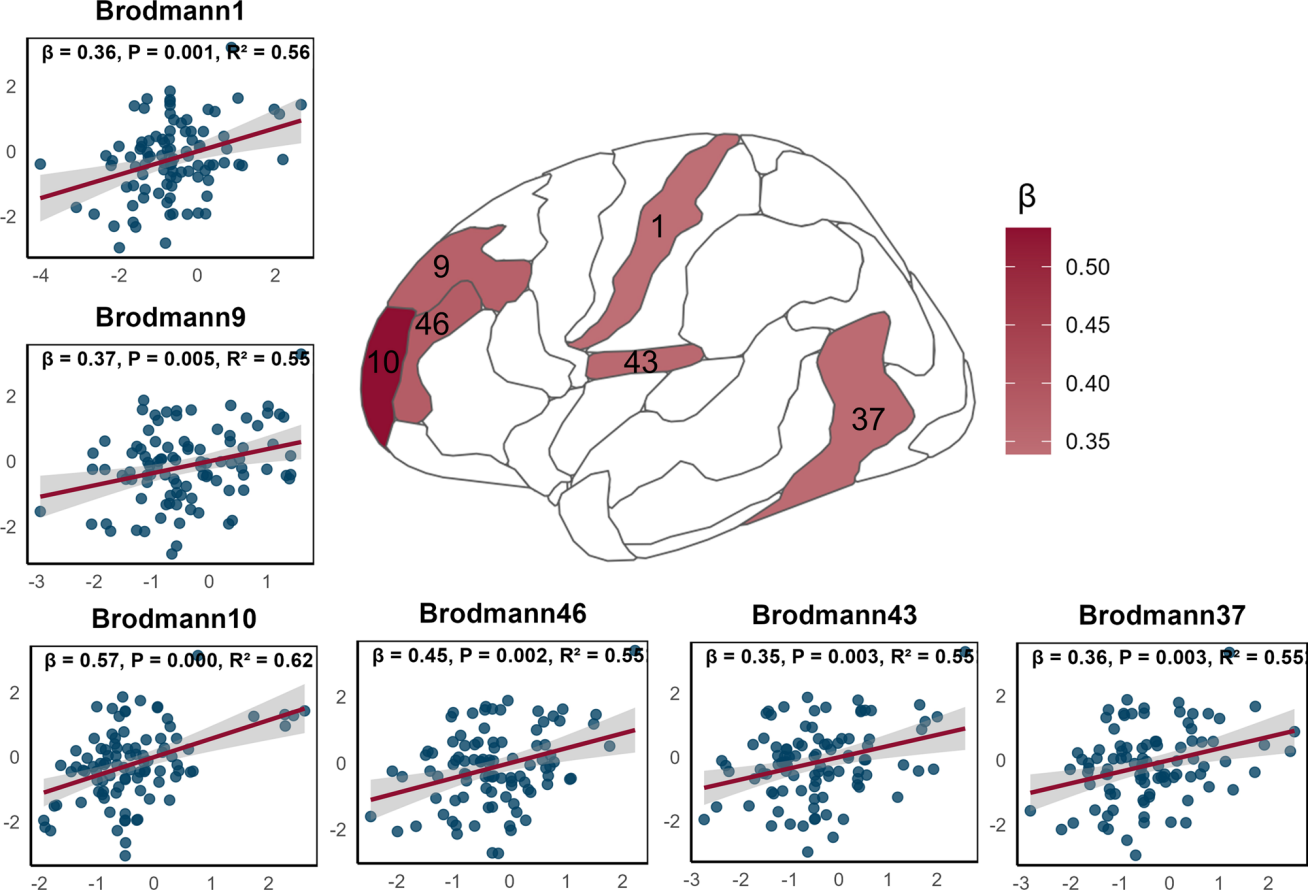
Associations between regional qMRI Z-scores and EDSS in pwMS. The plot shows representation of the Brodmann regions where R_2_* Z-scores show a significant correlation with EDSS levels in the multivariable linear regression equation [3]. The β values in the color bar represent the strength (and direction) of the association between R_2_* and EDSS. Each scatterplot represents a significantly associated region, where the x-axis denotes the R_2_* Z-score and the y-axis represents adjusted EDSS by sex, phenotype, and disease duration. The red regression line thus illustrates the partial residual fit showing the relationship after adjusting for covariates. Annotations within each panel indicate the β coefficient, the *P*_FDR_ (multiple comparison-adjusted p-value), and the adjusted R² for the model fitting. All brain regions included were preselected based on significant group differences between people with multiple sclerosis and healthy controls. BA46 with a potential group-level difference (*P*_FDR_ = 0.07) was also included. cGM: cortical grey matter. EDSS: Expanded Disability Status Scale.

We further examined the associations between regional brain Z-scores and EDSS scores in pwMS. Significant positive associations were observed only for R_2_* in cGM regions (*P*_FDR_ < 0.05), specifically in BA 1, 9, 10, 37, 43, and 46 (see Fig. 5). The most substantial effect was found in BA 10 (β = 0.53, *P*_FDR_ = 0.002). In contrast, no significant associations were found for R_1_ in cGM, nor for either R_1_ or R_2_* in sWM or WM bundles.

## 4 Discussion

In this study, we evaluated machine learning–based statistical strategies for harmonizing qMRI mapping metrics across sites. Specifically, we harmonized R_1_ maps acquired from three sites using similar MP2RAGE protocols, and R_2_* maps obtained from two sites with substantially different ME-GRE acquisition settings. Our results demonstrated that both the HBR-based B-spline and ComBat-GAM methods effectively reduced site-related variability under both matched and unmatched protocol conditions. We further presented that these models improved the stability of normative age modeling while maintaining the overall age-related pattern compared to the raw data. Moreover, the HBR-based normative framework effectively identified pathological deviations from normative distributions in the harmonized data, as evidenced by comparisons with the inter- and cross-site MS–HC differences observed in the raw datasets. Finally, Z-scores derived from harmonized pwMS data were associated with clinical disability, supporting the potential clinical application of this framework.

### 4.1 Sources of site variability

Most regions demonstrating relatively high ICCs before correction showed a decline in ICC after adjustment, suggesting that a considerable portion of the batch variance was confounded with strong age-related effects that were accounted for post-correction. The PCA analysis further indicated that the larger batch effects observed in cGM regions for both R_1_ and R_2_* (see Fig. 1a and b, respectively) are attributed to different factors. For R_1_, the presence of the younger cohort (Site 3) undergoing significant neurodevelopment, combined with the use of compressed sensing reconstruction (Mussard et al., 2020), may have influenced the measurements, particularly within the thin cortical regions. In the case of R_2_*, this bias is likely originating from partial volume artifacts close to the boundary between grey and white matter regions on the site 2 dataset. According to the results, many regions exhibited small R_2_* ICC values, indicating that, despite differences in imaging protocols and spatial resolution, the R_2_* distributions of specific regions may still be relatively consistent across different sites. This finding may reflect region-specific differences in response to macroscopic field inhomogeneities and local magnetic susceptibility, as variations in the distribution of iron and other paramagnetic substances across brain regions may make certain areas more susceptible to protocol-related effects.

### 4.2 Harmonization method comparison

We applied ComBat-GAM for group-level harmonization, accounting for the nonlinear age-related biological trajectories commonly observed in qMRI metrics (Pomponio et al., 2020), and additionally compared it with the standard ComBat model. Our results highlighted the limitations of the standard Combat model in the presence of strong age effects and demonstrated the necessity of ComBat-GAM to capture nonlinear age-related biological effects. As a complementary strategy, we further implemented the HBR framework, a method previously validated for pooling and analyzing large-scale structural and diffusion MRI data across multiple cohorts (Rutherford et al., 2022; Villalón-Reina et al., 2022). This framework enables individualized Z-score estimation, supporting subject-level assessment of both batch variance and pathological deviations (de Boer et al., 2024), and has been shown to outperform traditional harmonization techniques in preserving clinical information (Kia et al., 2022; Villalón-Reina et al., 2022). Notably, ComBat-GAM and HBR are Bayesian and machine learning–based partial pooling methods that generalize to new datasets. Once trained on multi-site data, they estimate parameters for unseen sites using prior distributions, ensuring consistent harmonization across datasets with comparable acquisition settings (Kia et al., 2022). This transferability has been demonstrated previously in morphological and diffusion-based studies (Fortin et al., 2017; Pomponio et al., 2020; Richter et al., 2022; Villalón-Reina et al., 2022). Consistent with these findings, our results further confirm the effectiveness of batch correction in multicenter relaxometry-based qMRI data. However, compared with EBS methods, HBR methods increase computational complexity and require larger datasets. Our limited sample size could lead to HBR methods failing to estimate homogeneous variability across batches, and thus being insensitive to site-specific nuances (de Boer et al., 2024).

According to the results, the GAM and HBR methods performed comparably, reducing between-site variance (as reflected by significant decreases in ICC and η^2^), thereby yielding more homogeneous cross-site distributions, closely aligned peak age estimates, and declines in RMSE. The limitation of the HBR method was observed in the peak age comparisons. As shown in Supplementary Materials (S.3.3), several regions (for example, R_1_ in BA 35 of cGM, BA 42 of sWM) exhibit significant discrepancies between GAM and B-spline in peak age estimation. These discrepancies were often accompanied by standard errors exceeding 1.5 times the IQR above the third quartile, suggesting instability in the estimates. These unstable peak ages were therefore excluded from further correlation analyses. However, the average GAM estimates tended to be closer to the raw data (<3 years), while the average B-spline estimates showed larger deviations (~10 years). This observation highlights the need for further validation of HBR-based harmonization results using extended datasets, particularly by comparing the harmonized results with the original distribution. In our analysis, we also observed that regions where the raw data exhibited minimal batch effects (low corrected ICC values in Supplementary Materials (S.3.1)) were subject to unexpected distortions after harmonization. For example, in the right CST (where the ICC was 0.06 and 0.08 before and after correction, respectively), both models resulted in shifts of over 10 years in the estimated peak ages. Similar shifts were also observed in other R_1_-based WM bundles such as FP, suggesting that in low-variance regions, the applied harmonization methods may overcorrect and disrupt meaningful biological patterns.

### 4.3 Assessing cross-site R_1_ harmonization

In the R_1_ setting, we evaluated the feasibility of combining multicenter datasets acquired with similar MP2RAGE protocols but covering different age segments. Notably, although all datasets were obtained using comparable MP2RAGE protocols, Site 3 employed a different scanner model from the same manufacturer, which may introduce subtle variability due to differences in hardware architecture and gradient performance, a source of systematic variability commonly encountered in qMRI data harmonization (Weiskopf et al., 2013). The three sites were selected to ensure comparable acquisition conditions while encompassing distinct age ranges, allowing us to specifically examine the biological effects of merging cohorts with different age distributions. This setting reflects a common challenge in multi-site lifespan studies, particularly considering the strong age dependence widely reported for qMRI metrics (Villalón-Reina et al., 2022). While structural and resting-state functional MRI data are widely available in large-scale open datasets, the systematic collection of qMRI data is still in its early stages. In this context, we examined whether R_1_ data collected using consistent MP2RAGE protocols and standardized processing pipelines could be integrated across sites and could accurately reflect normative physiological changes. According to the results, we observed that quadratic age-related models from the raw data of three sites aligned smoothly across most brain regions, indicating consistent developmental trends despite differences in cohort age coverage (Fig. 2a). The peak age estimates derived from raw and harmonized datasets showed minimal deviations, with mean differences of 0.7 years for GAM and 1.1 years for HBR. Moreover, peak ages estimated from raw data were correlated with those from both harmonization methods, and this consistency was maintained across distinct brain structures (Fig. 3a–c), suggesting that the uniformity of MP2RAGE acquisition parameters and the inherent stability of structural R_1_ maps permitted the direct merging of multi-site data without introducing substantial modeling bias. Notably, when using the standard ComBat model, the peak age shifts were more pronounced, and the RMSE was reduced but remained comparable to that of the raw data (Supplementary Materials (S.3.3 and S.3.5)), highlighting the effectiveness of both the GAM and HBR methods in controlling age-related variance. In parallel, both harmonization results supported the feasibility and robustness of combining datasets with partially overlapping age distributions for age modeling.

### 4.4 Assessing cross-site R_2_* harmonization

In the R_2_* setting, we evaluated the feasibility of combining qMRI metrics acquired with different imaging protocols and resolutions. In this analysis, the two datasets were reconstructed from unmatched ME-GRE protocols but were closely matched for age and sex distributions, minimizing biological variability and allowing us to focus on protocol- and resolution-related variances. This design complements the R_1_ analysis by addressing a different but equally relevant harmonization context, together capturing both biological pooling and technical harmonization within a controlled framework. As larger qMRI datasets become available from different sites, it will be crucial to test the generalizability of our findings. Despite differences in protocol parameters setting and image resolution, the peak ages derived from raw and harmonized data were significantly correlated, with only minimal shifts observed. These results indicate that when a consistent processing pipeline and age-matched populations are present, harmonization can enable the integration of qMRI data from different acquisition settings while preserving the integrity of age-related modeling.

### 4.5 Preservation of disease-related differences in harmonized data

To assess whether pathological deviations in qMRI measurements could be preserved after harmonization, we further quantified the difference between pwMS and HC. Our analysis showed widespread reductions in both R_1_ and R_2_* Z-scores across regions in pwMS, in line with previous MS literature (Lommers et al., 2019; Paling et al., 2012). These effects were particularly evident in sWM and WM bundles, where average group-level deviations approached or exceeded one standard deviation of the mean. The reduction in R_1_ likely reflects microstructural tissue damage in MS, including demyelination, axonal loss, and increased extracellular water content (Pontillo et al., 2023). These changes align with the known pathology of MS, which primarily affects WM integrity (Bando, 2020; Lassmann, 2014). R_2_* values were also predominantly reduced in pwMS, though to a slightly lesser extent than R_1_, which may reflect progressive demyelination rather than iron clearance (Bagnato et al., 2018; Rudko et al., 2014). Although reductions in R_1_ and R_2_* dominated the overall pattern, some regions in cGM exhibited increased values. These could reflect focal iron deposition (R_2_*) or reactive tissue changes such as gliosis (R_1_). Notably, the within-site raw comparisons findings were broadly consistent with the harmonized findings (Supplementary Materials (S.3.6)), with Supplementary Materials (S.4, Fig. S2b) additionally showing regional Z-score patterns that closely matched those observed in the harmonized Z-score maps in Figure 4, both in terms of effect direction and the overall distribution of regional deviations. After harmonization, more regions reached significance in cGM, and the generally reduced significant p-values across regions are likely due to the marked increase in the HC sample (from 101 to 388) and the resulting increase in statistical power. However, pooling healthy controls across sites introduced slightly greater variance and partially centered site-specific means toward the global reference distribution, resulting in smaller but statistically robust Cohen’s d values. In contrast, the cross-site raw comparison showed several regions with higher R_1_ and R_2_* in HCs than in pwMS (Supplementary Materials (S.3.6 and S.4, Fig. S2a), which conflicted with established pathology and prior literature (Granziera et al., 2021; Vrenken et al., 2006). Taken together, these head-to-head analyses indicate that harmonization preserves biologically meaningful disease-related deviations and stabilizes effect directions across sites. In contrast, unharmonized cross-site comparisons can yield misleading inferences about pathology.

Subsequent analyses further showed that only R_2_* deviations in cGM were correlated with EDSS scores, aligning with the fact that R_2_* reflects more specific susceptibility-related effects than R_1_ (Marques et al., 2017), and with findings linking cortical pathology in MS to disease progression (Andica et al., 2019; Parry et al., 2003). Notably, to minimize confounding from lesion-related signal alterations, our analysis focused exclusively on normal-appearing tissue, allowing us to isolate microstructural abnormalities more accurately from focal lesions, which have been extensively characterized in our previous MS studies (Calabrese et al., 2010; X. Chen et al., 2023; Granziera et al., 2021).

### 4.6 Limitations

Some limitations need to be acknowledged in this study. First, the overall sample size remains moderate; thus, the interpretation of patient-related Z-score differences should consider the influence of sample size and the characteristics of the reference distribution (HCs). Incorporating larger and more representative control data will increase the power to detect group differences. Caution is, thus, warranted when interpreting or comparing such results across studies with differing levels of data support (e.g., sample size, age range, and batch effects). In addition, the MS cohort was derived from a single center, which may limit the generalizability of the findings. Including multicenter data in future studies would enable a more comprehensive validation of harmonization robustness and its ability to preserve disease-related effects. Moreover, the BA-based cortical parcellation used in this study may not fully correspond to functionally or microstructurally defined regions. Prior work has shown that BA boundaries do not always align with myeloarchitectonic or functional brain divisions (Glasser et al., 2014), suggesting that function-based atlases may be more appropriate for future studies aiming to link qMRI metrics with behavioral or cognitive outcomes. While hemispheric differences were explicitly considered in the analysis of WM bundles (motivated by prior findings of asymmetric age-related trajectories (Slater et al., 2019)), cortical metrics were averaged across hemispheres. This choice follows conventions in prior morphometric studies (Giedd et al., 1996; Raz et al., 1995), but may obscure subtle lateralization effects relevant to cortical microstructure. Our models did not account for cognitive or educational covariates, which may influence qMRI-based brain-behavior relationships and could be included in future analyses to improve interpretability (Ghadery et al., 2015). Finally, this study is purely cross-sectional, which limits the ability to capture within-subject developmental or degenerative changes over time.

## 5 Conclusion

To conclude, this study demonstrates the feasibility of harmonizing qMRI metrics across multi-site datasets using machine learning-based statistical strategies. By evaluating both protocol-matched datasets with unaligned age distribution and datasets acquired using unmatched imaging protocols, we showed that harmonization can effectively reduce site-related variability while preserving biologically and pathologically relevant features. Importantly, both harmonization frameworks are model-based and can be extended to unseen sites, as the estimated parameters representing batch effects and nonlinear covariate terms can be applied to new data acquired with comparable sequences and preprocessing pipelines, facilitating the integration of additional centers in future studies with acquisition settings similar to those of the present cohorts. Our findings show the potential of harmonized qMRI mapping to support large-scale, cross-site investigations into brain development, aging, and pathology. This study, therefore, provides a foundation for future normative modeling efforts and contributes to the methodological advancement of quantitative mapping research.

## Supplementary Material

Supplementary Material

## Data Availability

The harmonization tools used in this study are open-source: ComBat-GAM (https://github.com/rpomponio/neuroHarmonize) and the HBR methods via PCNtoolkit (https://pcntoolkit.readthedocs.io/en/latest), with completed codes shared on (https://github.com/amarquand/PCNtoolkit/blob/master/pcntoolkit/regression_model/hbr.py). The in-house scripts for image processing are available on GitHub (https://github.com/JosePMarques/MP2RAGE-related-scripts and https://github.com/kschan0214/sepia). Details of the whole MRI data processing, including the open-source software and code sources used, are provided in Supplementary Materials (S.1). The original MR data and processing pipeline of the ABRIM (Site 1) study have been shared on the Radboud University data repository and are available upon request (https://doi.org/10.34973/7q0a-vj19 and https://doi.org/10.34973/m5t8-s843). MRI and associated data from Sites 2 and 3 cannot be made publicly available due to ethical and legal restrictions related to participant privacy, particularly involving pediatric and patient populations. Access to these data may be considered on a collaborative basis, subject to a formal data sharing agreement, approval from the requesting local ethics committee, and submission of a formal project proposal. Additional statistical analysis scripts are available in a public repository (https://doi.org/10.34973/19qh-1w93).

## References

[IMAG.a.1140-b1] A G Teixeira, R. P., Malik, S. J., & Hajnal, J. V. (2019). Fast quantitative MRI using controlled saturation magnetization transfer. Magnetic Resonance in Medicine, 81(2), 907–920. 10.1002/mrm.27442 30257044 PMC6492254

[IMAG.a.1140-b2] A G Teixeira, R. P., Neji, R., Wood, T. C., Baburamani, A. A., Malik, S. J., & Hajnal, J. V. (2020). Controlled saturation magnetization transfer for reproducible multivendor variable flip angle T1 and T2 mapping. Magnetic Resonance in Medicine, 84(1), 221–236. 10.1002/mrm.28109 31846122 PMC7154666

[IMAG.a.1140-b3] Andica, C., Hagiwara, A., Kamagata, K., Yokoyama, K., Shimoji, K., Saito, A., Takenaka, Y., Nakazawa, M., Hori, M., & Cohen-Adad, J. (2019). Gray matter alterations in early and late relapsing-remitting multiple sclerosis evaluated with synthetic quantitative magnetic resonance imaging. Scientific Reports, 9(1), 1–10. 10.1038/s41598-019-44615-3 31148572 PMC6544650

[IMAG.a.1140-b4] Bagnato, F., Hametner, S., Boyd, E., Endmayr, V., Shi, Y., Ikonomidou, V., Chen, G., Pawate, S., Lassmann, H., & Smith, S. (2018). Untangling the R2* contrast in multiple sclerosis: A combined MRI-histology study at 7.0 Tesla. PLoS One, 13(3), e0193839. 10.1371/journal.pone.0193839 29561895 PMC5862438

[IMAG.a.1140-b5] Bando, Y. (2020). Mechanism of demyelination and remyelination in multiple sclerosis. Clinical and Experimental Neuroimmunology, 11(S1), 14–21. 10.1111/cen3.12576

[IMAG.a.1140-b6] Barbosa, J. H. O., Santos, A. C., Tumas, V., Liu, M., Zheng, W., Haacke, E. M., & Salmon, C. E. G. (2015). Quantifying brain iron deposition in patients with Parkinson’s disease using quantitative susceptibility mapping, R2 and R2. Magnetic Resonance Imaging, 33(5), 559–565. 10.1016/j.mri.2015.02.021 25721997

[IMAG.a.1140-b7] Calabrese, M., Filippi, M., & Gallo, P. (2010). Cortical lesions in multiple sclerosis. Nature Reviews Neurology, 6(8), 438–444. 10.1038/nrneurol.2010.93 20625376

[IMAG.a.1140-b8] Carey, D., Caprini, F., Allen, M., Lutti, A., Weiskopf, N., Rees, G., Callaghan, M. F., & Dick, F. (2018). Quantitative MRI provides markers of intra-, inter-regional, and age-related differences in young adult cortical microstructure. NeuroImage, 182, 429–440. 10.1016/j.neuroimage.2017.11.066 29203455 PMC6189523

[IMAG.a.1140-b9] Chan, K.-S., & Marques, J. P. (2021). SEPIA—Susceptibility mapping pipeline tool for phase images. Neuroimage, 227, 117611. 10.1016/j.neuroimage.2020.117611 33309901

[IMAG.a.1140-b10] Chen, G., Taylor, P. A., Haller, S. P., Kircanski, K., Stoddard, J., Pine, D. S., Leibenluft, E., Brotman, M. A., & Cox, R. W. (2018). Intraclass correlation: Improved modeling approaches and applications for neuroimaging. Human Brain Mapping, 39(3), 1187–1206. 10.1002/hbm.23909 29218829 PMC5807222

[IMAG.a.1140-b11] Chen, X., Schädelin, S., Lu, P.-J., Ocampo-Pineda, M., Weigel, M., Barakovic, M., Ruberte, E., Cagol, A., Marechal, B., & Kober, T. (2023). Personalized maps of T1 relaxometry abnormalities provide correlates of disability in multiple sclerosis patients. NeuroImage: Clinical, 37, 103349. 10.1016/j.nicl.2023.103349 36801600 PMC9958406

[IMAG.a.1140-b12] Cieslak, M., Cook, P. A., He, X., Yeh, F.-C., Dhollander, T., Adebimpe, A., Aguirre, G. K., Bassett, D. S., Betzel, R. F., & Bourque, J. (2021). QSIPrep: An integrative platform for preprocessing and reconstructing diffusion MRI data. Nature Methods, 18(7), 775–778. 10.1038/s41592-021-01185-5 34155395 PMC8596781

[IMAG.a.1140-b13] de Boer, A. A., Bayer, J. M., Kia, S. M., Rutherford, S., Zabihi, M., Fraza, C., Barkema, P., Westlye, L. T., Andreassen, O. A., & Hinne, M. (2024). Non-Gaussian normative modelling with hierarchical Bayesian regression. Imaging Neuroscience, 2, 1–36. 10.1101/2022.10.05.510988 PMC12247560 40800395

[IMAG.a.1140-b14] Feng, R., Zhao, J., Wang, H., Yang, B., Feng, J., Shi, Y., Zhang, M., Liu, C., Zhang, Y., Zhuang, J., & Wei, H. (2021). MoDL-QSM: Model-based deep learning for quantitative susceptibility mapping. NeuroImage, 240, 118376. 10.1016/j.neuroimage.2021.118376 34246768

[IMAG.a.1140-b15] Filo, S., Shtangel, O., Salamon, N., Kol, A., Weisinger, B., Shifman, S., & Mezer, A. A. (2019). Disentangling molecular alterations from water-content changes in the aging human brain using quantitative MRI. Nature Communications, 10(1), 3403. 10.1038/s41467-019-11319-1 PMC6667463 31363094

[IMAG.a.1140-b16] Fischl, B. (2012). FreeSurfer. Neuroimage, 62(2), 774–781. 10.1016/j.neuroimage.2012.01.021 22248573 PMC3685476

[IMAG.a.1140-b17] Fortin, J.-P., Cullen, N., Sheline, Y. I., Taylor, W. D., Aselcioglu, I., Cook, P. A., Adams, P., Cooper, C., Fava, M., McGrath, P. J., McInnis, M., Phillips, M. L., Trivedi, M. H., Weissman, M. M., & Shinohara, R. T. (2018). Harmonization of cortical thickness measurements across scanners and sites. NeuroImage, 167, 104–120. 10.1016/j.neuroimage.2017.11.024 29155184 PMC5845848

[IMAG.a.1140-b18] Fortin, J.-P., Parker, D., Tunç, B., Watanabe, T., Elliott, M. A., Ruparel, K., Roalf, D. R., Satterthwaite, T. D., Gur, R. C., Gur, R. E., Schultz, R. T., Verma, R., & Shinohara, R. T. (2017). Harmonization of multi-site diffusion tensor imaging data. NeuroImage, 161, 149–170. 10.1016/j.neuroimage.2017.08.047 28826946 PMC5736019

[IMAG.a.1140-b19] Gebre, R. K., Senjem, M. L., Raghavan, S., Schwarz, C. G., Gunter, J. L., Hofrenning, E. I., Reid, R. I., Kantarci, K., Graff-Radford, J., Knopman, D. S., Petersen, R. C., Jack, C. R., & Vemuri, P. (2023). Cross–scanner harmonization methods for structural MRI may need further work: A comparison study. NeuroImage, 269, 119912. 10.1016/j.neuroimage.2023.119912 36731814 PMC10170652

[IMAG.a.1140-b20] Ghadery, C., Pirpamer, L., Hofer, E., Langkammer, C., Petrovic, K., Loitfelder, M., Schwingenschuh, P., Seiler, S., Duering, M., & Jouvent, E. (2015). R2* mapping for brain iron: Associations with cognition in normal aging. Neurobiology of Aging, 36(2), 925–932. 10.1016/j.neurobiolaging.2014.09.013 25443291

[IMAG.a.1140-b21] Giedd, J. N., Snell, J. W., Lange, N., Rajapakse, J. C., Casey, B. J., Kozuch, P. L., Vaituzis, A. C., Vauss, Y. C., Hamburger, S. D., & Kaysen, D. (1996). Quantitative magnetic resonance imaging of human brain development: Ages 4–18. Cerebral Cortex, 6(4), 551–559. 10.1093/cercor/6.4.551 8670681

[IMAG.a.1140-b23] Glasser, M. F., Goyal, M. S., Preuss, T. M., Raichle, M. E., & Van Essen, D. C. (2014). Trends and properties of human cerebral cortex: Correlations with cortical myelin content. NeuroImage, 93, 165–175. 10.1016/j.neuroimage.2013.03.060 23567887 PMC3795824

[IMAG.a.1140-b24] Granziera, C., Wuerfel, J., Barkhof, F., Calabrese, M., De Stefano, N., Enzinger, C., Evangelou, N., Filippi, M., Geurts, J. J., & Reich, D. S. (2021). Quantitative magnetic resonance imaging towards clinical application in multiple sclerosis. Brain, 144(5), 1296–1311. 10.1093/brain/awab029 33970206 PMC8219362

[IMAG.a.1140-b25] Jansen, M. G., Zwiers, M. P., Marques, J. P., Chan, K.-S., Amelink, J. S., Altgassen, M., Oosterman, J. M., & Norris, D. G. (2024). The Advanced BRain Imaging on ageing and Memory (ABRIM) data collection: Study design, data processing, and rationale. PLoS One, 19(6), e0306006. 10.1371/journal.pone.0306006 38905233 PMC11192316

[IMAG.a.1140-b26] Johnson, W. E., Li, C., & Rabinovic, A. (2007). Adjusting batch effects in microarray expression data using empirical Bayes methods. Biostatistics, 8(1), 118–127. 10.1093/biostatistics/kxj037 16632515

[IMAG.a.1140-b27] Karakuzu, A., Biswas, L., Cohen-Adad, J., & Stikov, N. (2022). Vendor-neutral sequences and fully transparent workflows improve inter-vendor reproducibility of quantitative MRI. Magnetic Resonance in Medicine, 88(3), 1212–1228. 10.1002/mrm.29292 35657066

[IMAG.a.1140-b28] Keuken, M. C., Bazin, P.-L., Backhouse, K., Beekhuizen, S., Himmer, L., Kandola, A., Lafeber, J. J., Prochazkova, L., Trutti, A., Schäfer, A., Turner, R., & Forstmann, B. U. (2017). Effects of aging on T_1_, T_2_*, and QSM MRI values in the subcortex. Brain Structure and Function, 222(6), 2487–2505. 10.1007/s00429-016-1352-4 28168364 PMC5541117

[IMAG.a.1140-b29] Kia, S. M., Huijsdens, H., Dinga, R., Wolfers, T., Mennes, M., Andreassen, O. A., Westlye, L. T., Beckmann, C. F., & Marquand, A. F. (2020). Hierarchical Bayesian regression for multi-site normative modeling of neuroimaging data. In A. L. Martel, P. Abolmaesumi, D. Stoyanov, D. Mateus, M. A. Zuluaga, S. K. Zhou, D. Racoceanu, & L. Joskowicz (Eds.), Medical Image Computing and Computer Assisted Intervention—MICCAI 2020 (Vol. 12267, pp. 699–709). Springer International Publishing. 10.1007/978-3-030-59728-3_68

[IMAG.a.1140-b30] Kia, S. M., Huijsdens, H., Rutherford, S., Boer, A. de, Dinga, R., Wolfers, T., Berthet, P., Mennes, M., Andreassen, O. A., Westlye, L. T., Beckmann, C. F., & Marquand, A. F. (2022). Closing the life-cycle of normative modeling using federated hierarchical Bayesian regression. PLoS One, 17(12), e0278776. 10.1371/journal.pone.0278776 36480551 PMC9731431

[IMAG.a.1140-b31] Koppers, S., Bloy, L., Berman, J. I., Tax, C. M. W., Edgar, J. C., & Merhof, D. (2019). Spherical harmonic residual network for diffusion signal harmonization. In E. Bonet-Carne, F. Grussu, L. Ning, F. Sepehrband, & C. M. W. Tax (Eds.), Computational diffusion MRI (pp. 173–182). Springer International Publishing. 10.1007/978-3-030-05831-9_14

[IMAG.a.1140-b32] Kruper, J., Yeatman, J. D., Richie-Halford, A., Bloom, D., Grotheer, M., Caffarra, S., Kiar, G., Karipidis, I. I., Roy, E., Chandio, B. Q., Garyfallidis, E., & Rokem, A. (2021). Evaluating the reliability of human brain white matter tractometry. Aperture Neuro, 2021(1), 25. 10.52294/e6198273-b8e3-4b63-babb-6e6b0da10669 PMC8785971 35079748

[IMAG.a.1140-b33] Lassmann, H. (2014). Mechanisms of white matter damage in multiple sclerosis. Glia, 62(11), 1816–1830. 10.1002/glia.22597 24470325

[IMAG.a.1140-b34] Leutritz, T., Seif, M., Helms, G., Samson, R. S., Curt, A., Freund, P., & Weiskopf, N. (2020). Multiparameter mapping of relaxation (R1, R2 *), proton density and magnetization transfer saturation at 3 T: A multicenter dual‐vendor reproducibility and repeatability study. Human Brain Mapping, 41(15), 4232–4247. 10.1002/hbm.25122 32639104 PMC7502832

[IMAG.a.1140-b35] Lommers, E., Simon, J., Reuter, G., Delrue, G., Dive, D., Degueldre, C., Balteau, E., Phillips, C., & Maquet, P. (2019). Multiparameter MRI quantification of microstructural tissue alterations in multiple sclerosis. NeuroImage: Clinical, 23, 101879. 10.1016/j.nicl.2019.101879 31176293 PMC6555891

[IMAG.a.1140-b36] Lorio, S., Lutti, A., Kherif, F., Ruef, A., Dukart, J., Chowdhury, R., Frackowiak, R. S., Ashburner, J., Helms, G., Weiskopf, N., & Draganski, B. (2014). Disentangling in vivo the effects of iron content and atrophy on the ageing human brain. NeuroImage, 103, 280–289. 10.1016/j.neuroimage.2014.09.044 25264230 PMC4263529

[IMAG.a.1140-b37] Lustig, M., Donoho, D., & Pauly, J. M. (2007). Sparse MRI: The application of compressed sensing for rapid MR imaging. Magnetic Resonance in Medicine, 58(6), 1182–1195. 10.1002/mrm.21391 17969013

[IMAG.a.1140-b38] Marquand, A. (2026). Amarquand/PCNtoolkit [Jupyter Notebook]. https://github.com/amarquand/PCNtoolkit (Original work published 2016)

[IMAG.a.1140-b39] Marques, J. P., Khabipova, D., & Gruetter, R. (2017). Studying cyto and myeloarchitecture of the human cortex at ultra-high field with quantitative imaging: R1, R2* and magnetic susceptibility. NeuroImage, 147, 152–163. 10.1016/j.neuroimage.2016.12.009 27939794

[IMAG.a.1140-b40] Marques, J. P., Kober, T., Krueger, G., van der Zwaag, W., Van de Moortele, P.-F., & Gruetter, R. (2010). MP2RAGE, a self bias-field corrected sequence for improved segmentation and T1-mapping at high field. Neuroimage, 49(2), 1271–1281. 10.1016/j.neuroimage.2009.10.002 19819338

[IMAG.a.1140-b41] Menks, W. M., Ekerdt, C., Janzen, G., Kidd, E., Lemhöfer, K., Fernández, G., & McQueen, J. M. (2022). Study protocol: A comprehensive multi-method neuroimaging approach to disentangle developmental effects and individual differences in second language learning. BMC Psychology, 10(1), 169. 10.1186/s40359-022-00873-x 35804430 PMC9270835

[IMAG.a.1140-b42] Mussard, E., Hilbert, T., Forman, C., Meuli, R., Thiran, J., & Kober, T. (2020). Accelerated MP2RAGE imaging using Cartesian phyllotaxis readout and compressed sensing reconstruction. Magnetic Resonance in Medicine, 84(4), 1881–1894. 10.1002/mrm.28244 32176826

[IMAG.a.1140-b43] Norbom, L. B., Ferschmann, L., Parker, N., Agartz, I., Andreassen, O. A., Paus, T., Westlye, L. T., & Tamnes, C. K. (2021). New insights into the dynamic development of the cerebral cortex in childhood and adolescence: Integrating macro-and microstructural MRI findings. Progress in Neurobiology, 204, 102109. 10.1016/j.pneurobio.2021.102109 34147583

[IMAG.a.1140-b44] Paling, D., Tozer, D., Wheeler-Kingshott, C., Kapoor, R., Miller, D. H., & Golay, X. (2012). Reduced R2′ in multiple sclerosis normal appearing white matter and lesions may reflect decreased myelin and iron content. Journal of Neurology, Neurosurgery & Psychiatry, 83(8), 785–792. 10.1136/jnnp-2012-302541 22626944

[IMAG.a.1140-b45] Parry, A., Clare, S., Jenkinson, M., Smith, S., Palace, J., & Matthews, P. M. (2003). MRI brain T1 relaxation time changes in MS patients increase over time in both the white matter and the cortex. Journal of Neuroimaging, 13(3), 234–239. 10.1111/j.1552-6569.2003.tb00184.x 12889170

[IMAG.a.1140-b46] Pomponio, R. (2025). Rpomponio/neuroHarmonize [Python]. https://github.com/rpomponio/neuroHarmonize (Original work published 2019).

[IMAG.a.1140-b47] Pomponio, R., Erus, G., Habes, M., Doshi, J., Srinivasan, D., Mamourian, E., Bashyam, V., Nasrallah, I. M., Satterthwaite, T. D., Fan, Y., Launer, L. J., Masters, C. L., Maruff, P., Zhuo, C., Völzke, H., Johnson, S. C., Fripp, J., Koutsouleris, N., Wolf, D. H.,… Davatzikos, C. (2020). Harmonization of large MRI datasets for the analysis of brain imaging patterns throughout the lifespan. NeuroImage, 208, 116450. 10.1016/j.neuroimage.2019.116450 31821869 PMC6980790

[IMAG.a.1140-b48] Pontillo, G., Petracca, M., Monti, S., Quarantelli, M., Lanzillo, R., Costabile, T., Carotenuto, A., Tortora, F., Elefante, A., Morra, V. B., Brunetti, A., Palma, G., & Cocozza, S. (2023). Clinical correlates of R1 relaxometry and magnetic susceptibility changes in multiple sclerosis: A multi-parameter quantitative MRI study of brain iron and myelin. European Radiology, 33(3), 2185–2194. 10.1007/s00330-022-09154-y 36241917 PMC9935712

[IMAG.a.1140-b49] Raz, N., Torres, I. J., & Acker, J. D. (1995). Age, gender, and hemispheric differences in human striatum: A quantitative review and new data from in vivo MRI morphometry. Neurobiology of Learning and Memory, 63(2), 133–142. 10.1006/nlme.1995.1013 7663886

[IMAG.a.1140-b50] R Core Team. (2013). R: A language and environment for statistical computing. Foundation for Statistical Computing, Vienna, Austria. 10.32614/r.manuals

[IMAG.a.1140-b100] Renauld, E., Boré, A., Poirier, C., Valcourt-Caron, A., Karan, P., Théberge, A., Théaud, G., Edde, M., Poulin, P., Girard, G., Houde, J.-C., Gagnon, A., St-Onge, E., Little, G., Legarreta, J. H., Thoumyre, S., Grenier, G., Yamani, Z. E., Pineda, M. O., … Descoteaux, M. (2026). Tractography analysis with the scilpy toolbox. Aperture Neuro, 6. 10.52294/001c.154022

[IMAG.a.1140-b51] Richter, S., Winzeck, S., Correia, M. M., Kornaropoulos, E. N., Manktelow, A., Outtrim, J., Chatfield, D., Posti, J. P., Tenovuo, O., Williams, G. B., Menon, D. K., & Newcombe, V. F. J. (2022). Validation of cross-sectional and longitudinal ComBat harmonization methods for magnetic resonance imaging data on a travelling subject cohort. Neuroimage: Reports, 2(4), 100136. 10.1016/j.ynirp.2022.100136 36507071 PMC9726680

[IMAG.a.1140-b52] Rudko, D. A., Solovey, I., Gati, J. S., Kremenchutzky, M., & Menon, R. S. (2014). Multiple sclerosis: Improved identification of disease-relevant changes in gray and white matter by using susceptibility-based MR imaging. Radiology, 272(3), 851–864. 10.1148/radiol.14132475 24828000

[IMAG.a.1140-b53] Rutherford, S., Kia, S. M., Wolfers, T., Fraza, C., Zabihi, M., Dinga, R., Berthet, P., Worker, A., Verdi, S., Ruhe, H. G., Beckmann, C. F., & Marquand, A. F. (2022). The normative modeling framework for computational psychiatry. Nature Protocols, 17(7), 1711–1734. 10.1038/s41596-022-00696-5 35650452 PMC7613648

[IMAG.a.1140-b54] Shams, Z., Norris, D. G., & Marques, J. P. (2019). A comparison of in vivo MRI based cortical myelin mapping using T1w/T2w and R1 mapping at 3T. PLoS One, 14(7), e0218089. 10.1371/journal.pone.0218089 31269041 PMC6609014

[IMAG.a.1140-b55] Slater, D. A., Melie‐Garcia, L., Preisig, M., Kherif, F., Lutti, A., & Draganski, B. (2019). Evolution of white matter tract microstructure across the life span. Human Brain Mapping, 40(7), 2252–2268. 10.1002/hbm.24522 30673158 PMC6865588

[IMAG.a.1140-b56] University Hospital, Basel, Switzerland. (2024). INsIDER: Imaging the Interplay Between Axonal Damage and Repair in Multiple Sclerosis (Clinical Trial Registration NCT05177523). clinicaltrials.gov. https://clinicaltrials.gov/study/NCT05177523

[IMAG.a.1140-b57] Van Essen, D. C. (2005). A Population-Average, Landmark- and Surface-based (PALS) atlas of human cerebral cortex. NeuroImage, 28(3), 635–662. 10.1016/j.neuroimage.2005.06.058 16172003

[IMAG.a.1140-b58] Van Gelderen, P., Jiang, X., & Duyn, J. H. (2016). Effects of magnetization transfer on T 1 contrast in human brain white matter. NeuroImage, 128, 85–95. 10.1016/j.neuroimage.2015.12.032 26724780 PMC4762731

[IMAG.a.1140-b59] Villalón-Reina, J. E., Moreau, C. A., Nir, T. M., Jahanshad, N., Simons Variation in Individuals Project Consortium, Maillard, A., Romascano, D., Draganski, B., Lippé, S., Bearden, C. E., Kia, S. M., Marquand, A. F., Jacquemont, S., & Thompson, P. M. (2022). Multi-site normative modeling of diffusion tensor imaging metrics using hierarchical bayesian regression. In L. Wang, Q. Dou, P. T. Fletcher, S. Speidel, & S. Li (Eds.), Medical Image Computing and Computer Assisted Intervention—MICCAI 2022 (Vol. 13431, pp. 207–217). Springer Nature Switzerland. 10.1007/978-3-031-16431-6_20 PMC11524145 39479363

[IMAG.a.1140-b60] Vrenken, H., Geurts, J. J. G., Knol, D. L., van Dijk, L. N., Dattola, V., Jasperse, B., van Schijndel, R. A., Polman, C. H., Castelijns, J. A., Barkhof, F., & Pouwels, P. J. W. (2006). Whole-brain T1 mapping in multiple sclerosis: Global changes of normal-appearing gray and white matter. Radiology, 240(3), 811–820. 10.1148/radiol.2403050569 16868279

[IMAG.a.1140-b61] Wang, Y., & Liu, T. (2015). Quantitative susceptibility mapping (QSM): Decoding MRI data for a tissue magnetic biomarker. Magnetic Resonance in Medicine, 73(1), 82–101. 10.1002/mrm.25358 25044035 PMC4297605

[IMAG.a.1140-b62] Wang, Y., Van Gelderen, P., De Zwart, J. A., & Duyn, J. H. (2020). B0-field dependence of MRI T1 relaxation in human brain. NeuroImage, 213, 116700. 10.1016/j.neuroimage.2020.116700 32145438 PMC7165058

[IMAG.a.1140-b63] Weiskopf, N., Suckling, J., Williams, G., Correia, M. M., Inkster, B., Tait, R., Ooi, C., Bullmore, E. T., & Lutti, A. (2013). Quantitative multi-parameter mapping of R1, PD*, MT, and R2* at 3T: A multi-center validation. Frontiers in Neuroscience, 7, 46379. 10.3389/fnins.2013.00095 PMC3677134 23772204

[IMAG.a.1140-b64] Yeatman, J. D., Wandell, B. A., & Mezer, A. A. (2014). Lifespan maturation and degeneration of human brain white matter. Nature Communications, 5(1), 4932. 10.1038/ncomms5932 PMC4238904 25230200

