# Supplementary material for: Cross-site quantitative MRI harmonization: The impact on age modeling in health and disease
Source: Imaging Neurosci (Camb). 2026 Feb 24;4:IMAG.a.1140. doi: 10.1162/IMAG.a.1140 (PMC12934009; doi:10.1162/IMAG.a.1140)
Supplement: Supplementary Material [file IMAG.a.1140_supp.pdf]

Supplementary Materials

S. 1. Descriptions of MRI Data Processing

S. 1. 1 Image Reconstruction

Preprocessing steps were conducted separately for the MP2RAGE and ME-GRE sequences to generate R_1, and R_2*. For the MP2RAGE data to generate the T_1 maps, preprocessing involved transmit field inhomogeneity correction (B1+ correction) to address variations in the radiofrequency field (Marques & Gruetter, 2013). From the T_1 maps, R_1 maps were calculated (R_1=1/T_1), and these values will be discussed throughout this work. Background noise removal of the MP2RAGE data was obtained using in-house scripts (O'Brien et al., 2014).

For the ME-GRE data, preprocessing included phase unwrapping and background field removal, followed by R_2* map reconstruction using the SEPIA toolbox (version 1.2.2.4)(Chan & Marques, 2021). The pipeline used has been extensively described in a publication using Site 1 data (Jansen et al., 2024) and relied on the following methods: ROMEO for field calculation; multiparameter HARP for background field removal (Li et al., 2011); LPCNN for dipole inversion (Lai et al., 2020); ARLO technique for R_2* maps computation (Pei et al., 2015).

R_2* maps were co-registered to the R_1 space. A rigid body transformation matrix between the first echo of the ME-GRE sequence and the second inversion time image from the MP2RAGE sequence was obtained using FSL (Jenkinson et al., 2012) and was subsequently applied to bring the R_2* maps to the R_1 map space.

Diffusion-weighted images (DWIs) were preprocessed using the default settings of QSIprep (version 0.18.0) (Cieslak et al., 2021), which included denoising and motion correction.

S. 1. 2. Cortical Parcellation

For cortical segmentation, the Second Inversion Time and Uniform images from the MP2RAGE sequence were multiplied to mask out the noise background (Fujimoto et al., 2014). Then, these output images were applied in FreeSurfer (Fischl, 2012) recon-all pipeline for brain segmentation. T1-weighted images from patients were lesion-filled by FSL earlier. Cortical parcellation was performed using the PALS-B12 Atlas (Van Essen, 2005) provided by FreeSurfer, with further in-house refinements to correct inaccurate labels. Manual quality control steps, including skull-stripping correction, pial surface error correction, and intensity normalization, were implemented before and after segmentation to ensure the accuracy of the parcellation and the extraction of quantitative measurements.

S. 1. 3. White Matter Tractography

To obtain bundle information, a pyAFQ (Kruper et al., 2021) pipeline of QSIprep was applied. This pipeline uses multi-shell and multi-tissue spherical deconvolution methods to estimate fiber orientations to extract a specific set of eighteen bundles, including Callosum Forceps Minor (FA), Callosum Forceps Major (FP), and the following bilateral tracts: Arcuate (ARC), Posterior Arcuate Fasciculus (pARC), Thalamic Radiation (ATR), Cingulum Cingulate (CGC), Corticospinal (CST), Inferior Fronto-Occipital Fasciculus (IFO), Inferior Longitudinal Fasciculus (ILF), and Superior Longitudinal Fasciculus (SLF).

S. 1. 4. Quantitative Mapping Across Brain Structures

Quantitative surface maps were created by projecting qMRI maps onto the brain surface derived from FreeSurfer. qMRI values in the cortex were extracted in the middle cortical layer between the white and pial surfaces using FreeSurfer with settings 0.5 and -0.5, respectively (Fischl, 2012). The average qMRI measurements (R_1, R_2* values) derived from the individual medians were calculated in each region separately for each hemisphere, considering only vertices that passed automatic quality assurance per region as described in a previous study (Shams et al., 2019).

Fiber tracts were co-registered to R_1 space (in the same space with R_2*) using the transformation matrix computed from the flirt by FSL (Jenkinson et al., 2012), co-registration of DWI data (brain-masked and distortion-corrected images) with the brain-masked second inversion time images of the MP2RAGE. The SCILPY toolbox (*GitHub - Scilus/Scilpy: The Sherbrooke Connectivity Imaging Lab (SCIL) Python dMRI Processing Toolbox*, n.d.) was used to extract median qMRI values in each fiber tract, and the group-level averages were subsequently calculated.

Notably, this step did not assign values to lesion tissues using the lesion mask for the patients.

S. 1. 5. Reference

Chan, K.-S., & Marques, J. P. (2021). SEPIA—Susceptibility mapping pipeline tool for phase images. *Neuroimage*, *227*, 117611.

Fischl, B. (2012). FreeSurfer. *Neuroimage*, *62*(2), 774–781.

Fujimoto, K., Polimeni, J. R., Van Der Kouwe, A. J., Reuter, M., Kober, T., Benner, T., Fischl, B., & Wald, L. L. (2014). Quantitative comparison of cortical surface reconstructions from MP2RAGE and multi-echo MPRAGE data at 3 and 7 T. *Neuroimage*, *90*, 60–73.

GitHub—Scilus/scilpy: The Sherbrooke Connectivity Imaging Lab (SCIL) Python dMRI processing toolbox. (n.d.).

Jansen, M. G., Zwiers, M. P., Marques, J. P., Chan, K.-S., Amelink, J. S., Altgassen, M., Oosterman, J. M., & Norris, D. G. (2024). The Advanced BRain Imaging on ageing and Memory (ABRIM) data collection: Study design, data processing, and rationale. *PloS One*, *19*(6), e0306006. https://doi.org/10.1371/journal.pone.0306006

Jenkinson, M., Beckmann, C. F., Behrens, T. E. J., Woolrich, M. W., & Smith, S. M. (2012). FSL. *NeuroImage*, *62*(2), 782–790. https://doi.org/10.1016/j.neuroimage.2011.09.015

Kruper, J., Yeatman, J. D., Richie-Halford, A., Bloom, D., Grotheer, M., Caffarra, S., Kiar, G., Karipidis, I. I., Roy, E., Chandio, B. Q., Garyfallidis, E., & Rokem, A. (2021).

Evaluating the Reliability of Human Brain White Matter Tractometry. *Aperture Neuro*, *2021*(1), 25. https://doi.org/10.52294/e6198273-b8e3-4b63-babb-6e6b0da10669

Lai, K.-W., Aggarwal, M., Van Zijl, P., Li, X., & Sulam, J. (2020). Learned Proximal Networks for Quantitative Susceptibility Mapping. In A. L. Martel, P. Abolmaesumi, D. Stoyanov, D. Mateus, M. A. Zuluaga, S. K. Zhou, D. Racoceanu, & L. Joskowicz (Eds.), *Medical Image Computing and Computer Assisted Intervention – MICCAI 2020* (Vol. 12262, pp. 125–135). Springer International Publishing.

Li, W., Wu, B., & Liu, C. (2011). Quantitative susceptibility mapping of human brain reflects spatial variation in tissue composition. *NeuroImage*, *55*(4), 1645–1656. https://doi.org/10.1016/j.neuroimage.2010.11.088

Marques, J. P., & Gruetter, R. (2013). New developments and applications of the MP2RAGE sequence-focusing the contrast and high spatial resolution R1 mapping. *PloS One*, *8*(7), e69294.

O'Brien, K. R., Kober, T., Hagmann, P., Maeder, P., Marques, J., Lazeyras, F., Krueger, G., & Roche, A. (2014). Robust T1-weighted structural brain imaging and morphometry at 7T using MP2RAGE. *PloS One*, *9*(6), e99676.

Pei, M., Nguyen, T. D., Thimmappa, N. D., Salustri, C., Dong, F., Cooper, M. A., Li, J., Prince, M. R., & Wang, Y. (2015). Algorithm for fast monoexponential fitting based on Auto-Regression on Linear Operations (ARLO) of data. *Magnetic Resonance in Medicine*, *73*(2), 843–850. https://doi.org/10.1002/mrm.25137

Shams, Z., Norris, D. G., & Marques, J. P. (2019). A comparison of in vivo MRI based cortical myelin mapping using T1w/T2w and R1 mapping at 3T. *PloS One*, *14*(7), e0218089.

Van Essen, D. C. (2005). A Population-Average, Landmark- and Surface-based (PALS) atlas of human cerebral cortex. *NeuroImage*, *28*(3), 635–662.

S. 2. Descriptions of B-spline Harmonization

S. 2. 1 Method

In this step, we employed the Hierarchical Bayesian Regression-based B-spline model implemented in the PCN toolkit (Marquand et al., 2019) to estimate the mean and variance of each regional qMRI measure at a specific age and compute the corresponding z-score. The Site 1 dataset, which covered the widest age range, served as the reference dataset. Data from Sites 2 and 3 (changing dataset) were projected into the reference space of Site 1 by back-transforming z-scores, effectively correcting for site effects and allowing the pooling of data across sites. In this step, stratified five-fold cross-validation was used to ensure consistency in both site data and sex ratio across folds after data pooling from different sites. In each fold, 80% of the data was used for model training (20% for validation). The root mean square error was employed to identify the best model among all folds. Harmonized data in the changing datasets were then obtained by inversely transforming the corresponding z-scores to the reference dataset model. The effectiveness of different harmonization methods was evaluated based on the mean, standard deviation, and coefficient of variation (CV) at the overall level.

The model imposes a hierarchical structure to explain variation in the data (de Boer et al., 2024). At the top level, the nth observation y_n is assumed to vary around a mean μ_n with variance σ_n:

$$y_n \sim N\,(y_n \mid \mu_n, \sigma_n)$$

with n ∈ (1, ..., n samples). On a second level, μ_n and σ_n are modelled as the output of linear regression based on the input matrix:

$$\mu_n = w_\mu^T\ \phi + \tau_\mu$$

$$\sigma_n = w_\sigma^T\ \phi + \tau_\sigma$$

Here, φ describes the full, dummy-coded regression matrix including additional b-spline columns based on the input matrix; w describes a vector containing the corresponding (slope) regression parameters, and τ describes the intercept offset of that regression of the input matrix onto μ and σ, respectively.

Batch effects (sex, site) are modelled by allowing the intercept (τ) and slope (w) parameters of μ and σ to vary with a group standard deviation σ, multiplied by an offset v_b around a mean μ_b for all batches b. This so-called non-center sampling approach helps to avoid specific errors during sampling. Further, priors are placed over each set of parameters θ containing batch effects, allowing them to learn from each other (random effect):

$\theta_b = \mu_\theta + \sigma_\theta v_b$

$v_b \sim N(0,1)$

$\mu_\theta \sim N(\mu_\theta \mid 0,1)$

$\sigma_\theta \sim N+(\sigma_\theta \mid 1)$

where $\theta_b \in \{w_\mu, w_\sigma, \tau_\mu, \tau_\sigma\}$ for batch $b \in \{1 ... n \text{ batches}\}$.

Lastly, a set of standard Gaussian priors was placed over all remaining parameters. Model estimation and inference were performed using the No-U-Turn sampler encoded in PyMC (version 5.4.1). The model was estimated using four chains, each drawing 1500 samples, from which the first 500 were discarded as a warm-up.

S. 2. 2 Reference

de Boer, A. A., Bayer, J. M., Kia, S. M., Rutherford, S., Zabihi, M., Fraza, C., Barkema, P., Westlye, L. T., Andreassen, O. A., & Hinne, M. (2024). Non-Gaussian normative modelling with hierarchical Bayesian regression. *Imaging Neuroscience*, *2*, 1–36.

Marquand, A. F., Kia, S. M., Zabihi, M., Wolfers, T., Buitelaar, J. K., & Beckmann, C. F. (2019). Conceptualizing mental disorders as deviations from normative functioning. *Molecular Psychiatry*, *24*(10), 1415–1424. https://doi.org/10.1038/s41380-019-0441-1

S. 3. eTables

S. 3.1 Inter-site ICC Analysis Using Raw Data

	R1			R2*		
Region	ICC	ICC(Cor.)	η²	ICC	ICC(Cor.)	η²
cGM						
BA1	0.69	0.38	0.17	0.12	0.25	0.10
BA2	0.75	0.42	0.14	0.04	0.17	0.07
BA3	0.77	0.60	0.31	0.01	0.03	0.01
BA4	0.69	0.28	0.12	0.00	0.07	0.03
BA5	0.78	0.56	0.21	0.01	0.11	0.04
BA6	0.76	0.40	0.13	0.00	0.16	0.06
BA7	0.78	0.50	0.17	0.03	0.16	0.06
BA8	0.72	0.22	0.06	0.03	0.25	0.10
BA9	0.68	0.12	0.04	0.06	0.23	0.10
BA10	0.67	0.20	0.10	0.26	0.40	0.19
BA11	0.70	0.17	0.05	0.80	0.84	0.63
BA17	0.79	0.64	0.33	0.01	0.06	0.02
BA18	0.80	0.62	0.28	0.02	0.15	0.06
BA19	0.80	0.59	0.23	0.06	0.24	0.10
BA20	0.80	0.60	0.24	0.79	0.85	0.66
BA21	0.75	0.38	0.12	0.91	0.92	0.80
BA22	0.73	0.25	0.07	0.50	0.70	0.44
BA23	0.81	0.63	0.28	0.07	0.20	0.08
BA24	0.72	0.41	0.15	0.30	0.49	0.25
BA25	0.29	0.80	0.45	0.85	0.86	0.67
BA26	0.56	0.24	0.08	0.13	0.23	0.09
BA27	0.72	0.39	0.12	0.27	0.38	0.17
BA28	0.60	0.17	0.05	0.78	0.81	0.58
BA29	0.76	0.52	0.19	0.19	0.29	0.13
BA30	0.81	0.63	0.27	0.04	0.13	0.05
BA31	0.82	0.65	0.29	0.08	0.23	0.09
BA32	0.69	0.21	0.06	0.08	0.26	0.11
BA33	0.46	0.06	0.03	0.52	0.60	0.34
BA35	0.76	0.55	0.24	0.04	0.15	0.06
BA36	0.68	0.30	0.11	0.89	0.91	0.77
BA37	0.72	0.29	0.08	0.35	0.57	0.31
BA38	0.65	0.18	0.05	0.92	0.93	0.81
BA39	0.79	0.51	0.18	0.11	0.31	0.14
BA40	0.77	0.43	0.14	0.12	0.35	0.16
BA41	0.73	0.44	0.19	0.04	0.17	0.07
BA42	0.73	0.42	0.18	0.10	0.29	0.13
BA43	0.69	0.21	0.06	0.06	0.24	0.10
BA44	0.73	0.30	0.09	0.06	0.30	0.13
BA45	0.72	0.21	0.06	0.30	0.44	0.21
BA46	0.70	0.17	0.05	0.15	0.32	0.14
BA47	0.73	0.25	0.07	0.75	0.77	0.53
sWM						
BA1	0.57	0.47	0.25	0.01	0.08	0.03
BA2	0.43	0.38	0.22	0.18	0.13	0.05
BA3	0.58	0.55	0.31	0.17	0.08	0.03

BA4	0.34	0.26	0.14	0.16	0.09	0.04
BA5	0.38	0.30	0.16	0.03	0.01	0.01
BA6	0.29	0.19	0.11	0.12	0.08	0.03
BA7	0.41	0.33	0.19	0.10	0.06	0.02
BA8	0.22	0.14	0.08	0.01	0.00	0.00
BA9	0.23	0.17	0.09	0.08	0.11	0.04
BA10	0.12	0.09	0.04	0.16	0.22	0.09
BA11	0.09	0.02	0.01	0.54	0.56	0.30
BA17	0.50	0.44	0.24	0.10	0.04	0.01
BA18	0.52	0.44	0.24	0.13	0.05	0.02
BA19	0.40	0.32	0.17	0.19	0.14	0.06
BA20	0.29	0.02	0.01	0.43	0.54	0.28
BA21	0.34	0.06	0.03	0.78	0.81	0.58
BA22	0.30	0.11	0.06	0.13	0.10	0.04
BA23	0.20	0.08	0.04	0.08	0.08	0.03
BA24	0.14	0.08	0.04	0.21	0.20	0.08
BA25	0.03	0.30	0.09	0.63	0.63	0.36
BA26	0.19	0.00	0.00	0.00	0.00	0.00
BA27	0.15	0.06	0.03	0.00	0.00	0.00
BA28	0.22	0.07	0.04	0.27	0.38	0.17
BA29	0.22	0.01	0.01	0.04	0.02	0.01
BA30	0.21	0.08	0.04	0.10	0.11	0.04
BA31	0.29	0.19	0.10	0.16	0.14	0.05
BA32	0.20	0.13	0.07	0.16	0.23	0.09
BA33	0.02	0.10	0.03	0.01	0.01	0.01
BA35	0.21	0.13	0.07	0.21	0.15	0.06
BA36	0.25	0.05	0.03	0.83	0.85	0.66
BA37	0.21	0.06	0.03	0.00	0.00	0.00
BA38	0.34	0.00	0.00	0.85	0.87	0.69
BA39	0.36	0.28	0.15	0.27	0.26	0.11
BA40	0.38	0.26	0.14	0.19	0.17	0.07
BA41	0.22	0.12	0.07	0.08	0.09	0.03
BA42	0.40	0.32	0.19	0.10	0.05	0.02
BA43	0.28	0.16	0.08	0.35	0.35	0.16
BA44	0.32	0.22	0.12	0.18	0.17	0.07
BA45	0.25	0.17	0.10	0.00	0.00	0.00
BA46	0.31	0.25	0.14	0.24	0.27	0.11
BA47	0.22	0.11	0.06	0.51	0.49	0.24
WM Bundles						
ARCL	0.08	0.03	0.01	0.09	0.15	0.06
ARCR	0.09	0.07	0.02	0.14	0.19	0.08
ATRL	0.14	0.06	0.03	0.02	0.14	0.05
ATRR	0.08	0.01	0.01	0.01	0.09	0.04
CGCL	0.13	0.04	0.02	0.12	0.17	0.07
CGCR	0.16	0.08	0.03	0.11	0.14	0.06
CSTL	0.10	0.00	0.00	0.20	0.26	0.11
CSTR	0.07	0.08	0.02	0.25	0.27	0.11
FA	0.15	0.05	0.02	0.00	0.07	0.03
FP	0.07	0.01	0.01	0.13	0.25	0.10
IFOL	0.11	0.00	0.01	0.05	0.14	0.06
IFOR	0.10	0.05	0.02	0.07	0.19	0.08

ILFL	0.09	0.00	0.01	0.00	0.00	0.00
ILFR	0.21	0.18	0.05	0.01	0.06	0.02
pARCL	0.05	0.00	0.00	0.05	0.05	0.02
pARCR	0.31	0.24	0.07	0.07	0.09	0.03
SLFL	0.06	0.00	0.00	0.11	0.15	0.06
SLFR	0.14	0.13	0.04	0.17	0.21	0.09

ICC was estimated from a linear mixed-effects model with a random intercept for site. ICC (cor.) represents the same intraclass correlation after adjusting for covariates (Age, Age², and Sex). η^2 was derived from the Type III ANOVA of the corresponding linear model, reflecting the proportion of variance explained by site after covariate adjustment. *ICC: intraclass correlation coefficient; BA: Brodmann Area; cGM: cortical grey matter; sWM: superficial white matter; WM: white matter; FA: Callosum Forceps Minor; FP: Callosum Forceps Major; ARC: Arcuate; pARC: Posterior Arcuate Fasciculus; ATR: Thalamic Radiation; CGC: Cingulum Cingulate; CST: Corticospinal; IFO: Inferior Fronto-Occipital Fasciculus; ILF: Inferior Longitudinal Fasciculus; SLF: Superior Longitudinal Fasciculus.L: leftl; R: right.*

S. 3.2 Post-Harmonization Batch Effect Analysis

	R1 Combat			R1 GAM			R1 B-spline			R2* Combat			R2* GAM			R2* B-spline		
Region	ICC	ICC (cor.)	η^2	ICC	ICC (cor.)	η^2	ICC	ICC (cor.)	η^2	ICC	ICC (cor.)	η^2	ICC	ICC (cor.)	η^2	ICC	ICC (cor.)	η^2
cGM																		
BA1	0.15	0.34	9.93E-02	0.49	NE	7.67E-04	0.53	NE	1.51E-05	0.02	NE	6.74E-05	0.01	NE	3.63E-06	0.02	NE	9.80E-05
BA10	0.19	0.41	1.28E-01	0.53	NE	1.78E-03	0.46	NE	2.41E-03	0.02	NE	7.01E-05	0.01	NE	1.96E-05	0.05	NE	1.68E-03
BA11	0.22	0.43	1.38E-01	0.55	NE	5.28E-04	0.54	NE	9.48E-04	0.01	NE	6.29E-07	0.01	NE	4.09E-05	0.03	NE	1.06E-05
BA17	0.08	0.26	7.17E-02	0.36	NE	2.06E-03	0.26	NE	3.89E-04	0.01	NE	1.33E-05	0.01	NE	7.82E-06	0.02	NE	3.73E-04
BA18	0.12	0.35	1.04E-01	0.49	NE	7.42E-04	0.41	NE	6.69E-04	0.04	NE	3.59E-05	0.03	NE	2.32E-05	0.06	NE	1.45E-03
BA19	0.16	0.43	1.36E-01	0.56	NE	3.83E-04	0.49	NE	3.96E-04	0.05	NE	6.56E-05	0.04	NE	1.68E-06	0.06	NE	4.45E-04
BA2	0.17	0.41	1.28E-01	0.57	NE	4.14E-05	0.60	NE	9.34E-05	0.03	NE	5.90E-05	0.02	NE	4.87E-05	0.06	NE	2.03E-03
BA20	0.26	0.27	7.30E-02	0.50	NE	1.35E-03	0.47	NE	2.63E-03	0.05	NE	5.82E-06	0.04	NE	1.21E-04	0.06	NE	4.87E-05
BA21	0.25	0.34	1.00E-01	0.55	NE	4.03E-04	0.50	NE	1.49E-03	0.02	NE	3.32E-05	0.02	NE	5.18E-04	0.07	NE	4.89E-04
BA22	0.30	0.42	1.34E-01	0.61	NE	7.88E-06	0.59	NE	1.22E-05	0.07	NE	8.49E-05	0.06	NE	3.71E-06	0.07	NE	1.60E-06
BA23	0.21	0.34	9.90E-02	0.53	NE	6.82E-04	0.54	NE	1.23E-03	0.03	NE	3.41E-05	0.02	NE	2.10E-05	0.01	NE	1.83E-03
BA24	0.21	0.24	6.37E-02	0.48	NE	1.12E-03	0.49	NE	2.39E-03	0.04	NE	1.58E-05	0.04	NE	7.73E-06	0.06	NE	1.67E-03
BA25	0.18	0.24	6.28E-02	0.45	0.02	8.59E-03	0.42	NE	2.09E-03	NE	NE	3.35E-06	NE	NE	4.35E-05	NE	NE	6.15E-04
BA26	0.07	0.12	3.09E-02	0.28	NE	1.73E-04	0.20	NE	1.67E-03	0.02	NE	8.08E-08	0.02	NE	6.55E-06	0.02	NE	3.38E-09
BA27	0.24	0.20	5.36E-02	0.43	NE	3.46E-03	0.34	NE	5.72E-03	0.01	NE	9.76E-06	0.01	NE	8.81E-06	0.01	NE	4.66E-07
BA28	0.09	0.29	8.07E-02	0.37	NE	3.02E-03	0.35	0.01	7.42E-03	0.01	NE	3.99E-07	0.01	NE	3.05E-05	0.03	NE	9.35E-04
BA29	0.17	0.25	6.73E-02	0.45	NE	1.15E-03	0.30	NE	1.85E-03	0.01	NE	8.14E-06	0.01	NE	8.12E-06	0.02	NE	2.04E-04
BA3	0.15	0.31	8.65E-02	0.47	NE	6.34E-04	0.47	NE	1.49E-03	0.00	NE	2.31E-05	NE	NE	7.75E-07	0.01	NE	1.02E-04
BA30	0.21	0.29	8.23E-02	0.50	NE	5.18E-04	0.42	NE	3.36E-03	0.02	NE	3.82E-05	0.02	NE	2.38E-05	0.02	NE	6.02E-05
BA31	0.21	0.35	1.03E-01	0.53	NE	1.71E-03	0.52	NE	2.14E-03	0.03	NE	3.72E-05	0.02	NE	1.24E-04	0.03	NE	6.77E-08
BA32	0.25	0.31	8.79E-02	0.53	NE	8.15E-04	0.50	NE	7.23E-04	0.04	NE	5.22E-05	0.03	NE	4.52E-06	0.03	NE	4.56E-05
BA33	0.18	0.15	4.07E-02	0.37	NE	1.27E-03	0.41	NE	1.48E-03	0.01	NE	7.15E-06	0.01	NE	2.49E-06	0.04	NE	2.51E-03
BA35	0.20	0.20	5.35E-02	0.42	NE	2.72E-03	0.30	NE	1.63E-03	0.03	NE	5.32E-06	0.02	NE	7.86E-06	0.01	NE	5.21E-04
BA36	0.11	0.36	1.08E-01	0.48	NE	1.74E-03	0.34	0.02	9.48E-03	0.01	NE	6.84E-05	0.01	NE	1.74E-04	0.03	NE	4.02E-04
BA37	0.23	0.36	1.08E-01	0.58	NE	3.38E-05	0.49	NE	1.62E-03	0.05	NE	2.58E-05	0.05	NE	8.77E-06	0.07	NE	7.25E-04
BA38	0.25	0.20	5.33E-02	0.49	NE	4.92E-04	0.41	NE	3.90E-03	0.01	NE	4.24E-05	0.01	NE	1.68E-03	0.04	NE	1.03E-04
BA39	0.24	0.42	1.32E-01	0.57	NE	5.49E-04	0.52	NE	4.03E-03	0.05	NE	5.25E-05	0.04	NE	3.39E-05	0.05	NE	4.05E-05
BA4	0.21	0.43	1.36E-01	0.58	NE	4.97E-04	0.47	NE	1.03E-03	0.08	NE	7.16E-05	0.07	NE	3.99E-06	0.11	NE	1.79E-03
BA40	0.24	0.45	1.45E-01	0.59	NE	9.53E-04	0.55	NE	1.09E-03	0.06	NE	4.16E-05	0.05	NE	1.29E-05	0.03	NE	5.78E-04

BA41	0.22	0.27	7.46E-02	0.52	NE	2.71E-04	0.46	NE	2.82E-04	0.04	NE	3.43E-05	0.03	NE	2.58E-05	0.03	NE	2.18E-12
BA42	0.22	0.32	9.02E-02	0.50	NE	1.79E-03	0.42	NE	3.79E-03	0.05	NE	7.91E-05	0.04	NE	1.34E-05	0.05	NE	3.12E-07
BA43	0.24	0.37	1.09E-01	0.58	NE	1.43E-04	0.54	NE	1.24E-06	0.05	NE	2.60E-05	0.04	NE	4.35E-06	0.08	NE	1.50E-03
BA44	0.28	0.40	1.25E-01	0.61	NE	3.68E-06	0.55	NE	2.69E-03	0.07	NE	7.74E-05	0.06	NE	2.07E-06	0.06	NE	5.33E-04
BA45	0.27	0.44	1.41E-01	0.62	NE	3.94E-04	0.57	NE	1.82E-03	0.02	NE	1.78E-05	0.02	NE	6.48E-05	0.06	NE	1.28E-03
BA46	0.25	0.42	1.31E-01	0.56	NE	2.58E-03	0.59	0.00	5.04E-03	0.04	NE	7.94E-05	0.02	NE	1.74E-04	0.04	NE	3.82E-05
BA47	0.28	0.43	1.36E-01	0.60	NE	3.28E-04	0.52	NE	2.06E-03	0.00	NE	3.63E-04	0.01	NE	6.24E-04	0.03	NE	2.10E-05
BA5	0.12	0.33	9.64E-02	0.49	NE	3.43E-04	0.50	NE	2.74E-03	0.03	NE	5.04E-05	0.02	NE	8.60E-06	0.04	NE	4.65E-04
BA6	0.26	0.40	1.24E-01	0.57	NE	2.33E-03	0.57	NE	6.32E-04	0.09	NE	8.64E-05	0.08	NE	3.68E-05	0.11	NE	1.13E-03
BA7	0.17	0.45	1.47E-01	0.58	NE	6.52E-06	0.56	NE	4.70E-04	0.04	NE	6.57E-05	0.03	NE	5.76E-06	0.04	NE	1.18E-06
BA8	0.28	0.41	1.29E-01	0.60	NE	7.46E-05	0.60	NE	4.17E-04	0.07	NE	1.05E-04	0.06	NE	1.92E-05	0.10	NE	1.97E-03
BA9	0.25	0.42	1.31E-01	0.59	NE	4.10E-04	0.58	NE	1.41E-03	0.04	NE	1.09E-04	0.03	NE	3.46E-05	0.04	NE	6.94E-05
sWM																		
BA1	0.02	0.15	3.91E-02	0.04	NE	3.09E-03	0.00	0.03	1.11E-02	0.02	NE	8.11E-05	0.02	NE	2.38E-07	0.03	NE	3.77E-07
BA10	0.12	0.25	6.79E-02	0.03	NE	1.67E-03	0.03	NE	4.15E-03	NE	NE	6.35E-05	NE	NE	2.09E-05	NE	NE	2.64E-04
BA11	0.06	0.35	1.03E-01	0.12	NE	4.66E-04	0.02	NE	2.82E-03	NE	NE	2.79E-05	NE	NE	1.58E-05	NE	NE	1.00E-03
BA17	0.04	0.06	1.76E-02	0.01	NE	8.70E-04	0.01	NE	1.23E-03	0.02	NE	1.82E-05	0.02	NE	1.50E-05	0.02	NE	9.83E-05
BA18	0.06	0.15	3.94E-02	0.03	NE	1.01E-03	0.00	0.00	5.56E-03	0.03	NE	7.25E-05	0.03	NE	1.29E-05	0.05	NE	9.88E-04
BA19	0.14	0.23	6.21E-02	0.03	NE	1.38E-03	0.03	NE	2.68E-03	0.01	NE	7.25E-05	0.01	NE	7.85E-06	0.02	NE	1.55E-04
BA2	0.14	0.12	3.15E-02	0.05	NE	3.87E-03	0.03	NE	3.37E-03	0.01	NE	5.09E-05	0.01	NE	1.15E-05	0.00	NE	4.24E-04
BA20	0.00	0.33	9.66E-02	0.19	NE	6.64E-04	0.18	NE	1.98E-03	0.01	NE	6.20E-05	0.01	NE	5.97E-07	0.01	NE	4.94E-05
BA21	0.02	0.35	1.04E-01	0.17	NE	7.25E-04	0.09	NE	4.34E-03	NE	NE	2.53E-05	NE	NE	2.27E-04	NE	NE	1.01E-03
BA22	0.02	0.31	8.91E-02	0.13	NE	1.01E-03	0.01	NE	3.67E-03	0.00	NE	1.06E-04	0.00	NE	6.47E-06	0.00	NE	9.12E-05
BA23	0.13	0.25	6.87E-02	0.05	NE	4.37E-05	0.02	NE	5.22E-03	NE	NE	8.21E-06	NE	NE	4.89E-07	NE	NE	6.59E-05
BA24	0.11	0.17	4.48E-02	0.03	NE	2.85E-04	0.02	NE	3.23E-03	NE	NE	3.47E-05	NE	NE	1.63E-05	0.01	NE	1.44E-03
BA25	0.03	0.30	8.31E-02	0.12	NE	2.19E-03	0.00	NE	1.47E-03	NE	NE	1.96E-04	NE	NE	4.47E-06	0.00	NE	2.87E-04
BA26	0.00	0.22	5.72E-02	0.16	NE	7.71E-04	0.08	NE	2.79E-04	NE	NE	8.13E-06	NE	NE	8.77E-06	0.00	NE	2.45E-04
BA27	0.06	0.07	2.05E-02	0.24	NE	2.54E-04	0.18	NE	1.53E-03	0.02	NE	3.52E-05	0.02	NE	3.35E-06	0.03	NE	4.07E-04
BA28	0.00	0.23	6.28E-02	0.25	NE	4.09E-04	0.21	NE	1.36E-03	0.01	NE	7.50E-05	0.01	NE	1.01E-04	0.00	NE	2.50E-04
BA29	0.00	0.22	5.92E-02	0.17	NE	6.88E-05	0.09	0.01	7.14E-03	NE	NE	3.07E-05	0.00	NE	2.20E-04	0.00	NE	7.48E-05
BA3	0.03	0.02	8.95E-03	0.01	NE	1.67E-03	0.00	0.02	9.97E-03	0.03	NE	8.01E-05	0.02	NE	1.60E-06	0.03	NE	5.38E-05
BA30	0.11	0.23	6.20E-02	0.03	NE	3.25E-04	0.01	NE	3.49E-03	NE	NE	1.72E-05	NE	NE	1.89E-05	NE	NE	7.76E-08
BA31	0.15	0.21	5.62E-02	0.03	NE	1.75E-03	0.02	0.01	7.35E-03	NE	NE	4.81E-05	NE	NE	6.56E-07	0.00	NE	1.14E-04
BA32	0.15	0.21	5.72E-02	0.03	NE	2.16E-03	0.02	0.01	6.93E-03	NE	NE	8.32E-05	NE	NE	2.70E-05	NE	NE	5.76E-04
BA33	0.02	0.21	5.72E-02	0.09	NE	8.30E-04	0.22	NE	1.33E-03	NE	NE	6.04E-05	NE	NE	8.51E-06	NE	NE	1.60E-03

BA35	0.00	0.20	5.44E-02	0.18	NE	1.26E-04	0.24	NE	4.43E-04	0.01	NE	1.26E-05	0.00	NE	1.07E-05	0.02	NE	7.55E-04
BA36	0.02	0.24	6.56E-02	0.28	NE	3.48E-04	0.25	NE	4.07E-04	0.00	NE	6.30E-06	0.00	NE	3.95E-04	0.04	NE	2.06E-03
BA37	0.07	0.30	8.60E-02	0.07	NE	1.17E-03	0.03	NE	3.93E-03	0.00	NE	2.09E-04	0.00	NE	6.50E-06	NE	NE	4.67E-05
BA38	0.00	0.31	8.96E-02	0.28	NE	1.38E-03	0.18	NE	2.16E-03	NE	NE	3.82E-05	NE	NE	1.55E-03	0.03	NE	2.31E-03
BA39	0.13	0.21	5.63E-02	0.03	NE	2.12E-03	0.03	NE	3.43E-03	0.00	NE	7.57E-05	NE	NE	4.27E-06	0.01	NE	1.33E-04
BA4	0.17	0.18	4.72E-02	0.05	NE	4.60E-03	0.11	0.03	1.06E-02	0.02	NE	9.67E-05	0.02	NE	1.81E-05	0.02	NE	5.21E-05
BA40	0.06	0.24	6.63E-02	0.05	NE	2.10E-03	0.00	NE	5.94E-03	0.00	NE	1.12E-04	NE	NE	5.39E-06	0.01	NE	9.70E-05
BA41	0.17	0.17	4.39E-02	0.04	NE	3.40E-04	0.04	NE	1.82E-03	NE	NE	6.01E-05	NE	NE	1.06E-07	NE	NE	7.04E-05
BA42	0.19	0.16	4.12E-02	0.06	NE	2.16E-03	0.11	NE	2.54E-03	0.02	NE	5.92E-05	0.01	NE	3.25E-05	0.03	NE	9.62E-04
BA43	0.07	0.25	6.83E-02	0.05	NE	1.28E-03	0.02	NE	1.12E-03	NE	NE	8.69E-05	NE	NE	3.23E-06	NE	NE	3.95E-04
BA44	0.19	0.15	4.05E-02	0.06	NE	1.76E-03	0.05	NE	3.81E-03	NE	NE	7.67E-05	NE	NE	5.55E-06	NE	NE	1.22E-03
BA45	0.07	0.25	6.95E-02	0.04	NE	2.22E-03	0.01	NE	3.53E-03	NE	NE	9.97E-05	NE	NE	6.81E-05	NE	NE	1.29E-05
BA46	0.13	0.19	5.08E-02	0.03	NE	2.66E-03	0.05	NE	4.97E-03	NE	NE	6.86E-05	NE	NE	1.28E-09	NE	NE	1.48E-04
BA47	0.04	0.31	8.87E-02	0.14	NE	6.71E-04	0.06	NE	4.25E-03	NE	NE	5.39E-05	NE	NE	2.00E-04	NE	NE	2.23E-04
BA5	0.10	0.15	4.04E-02	0.02	NE	2.37E-03	0.01	0.04	1.27E-02	0.00	NE	6.70E-05	NE	NE	3.02E-05	0.00	NE	3.37E-06
BA6	0.17	0.19	4.95E-02	0.05	NE	2.71E-03	0.06	0.04	1.42E-02	0.01	NE	1.39E-04	0.00	NE	9.31E-05	0.00	NE	1.19E-03
BA7	0.09	0.19	4.95E-02	0.02	NE	3.67E-03	0.03	0.00	6.68E-03	0.01	NE	8.78E-05	0.00	NE	4.48E-08	0.01	NE	2.42E-04
BA8	0.10	0.26	6.98E-02	0.03	NE	2.60E-03	0.03	0.01	7.65E-03	NE	NE	1.05E-04	NE	NE	3.96E-06	NE	NE	9.65E-04
BA9	0.14	0.24	6.63E-02	0.03	NE	2.94E-03	0.08	0.02	9.22E-03	NE	NE	7.90E-05	NE	NE	4.73E-06	NE	NE	7.53E-04
WM bundles																		
ARCL	0.13	0.12	3.21E-02	0.03	NE	1.47E-03	0.02	NE	2.68E-03	NE	NE	3.12E-05	NE	NE	1.33E-05	NE	NE	1.45E-03
ARCR	0.15	0.10	2.64E-02	0.05	NE	1.14E-03	0.02	NE	1.58E-03	NE	NE	9.02E-06	NE	NE	1.35E-05	NE	NE	1.65E-04
ATRL	0.17	0.24	6.51E-02	0.05	NE	1.98E-03	0.04	NE	2.35E-03	0.02	NE	3.29E-05	0.02	NE	4.37E-05	0.03	NE	4.45E-05
ATRR	0.15	0.20	5.47E-02	0.03	NE	1.36E-03	0.03	NE	4.17E-03	0.02	NE	7.50E-05	0.02	NE	2.34E-05	0.02	NE	2.30E-06
CGCL	0.12	0.16	4.22E-02	0.04	NE	1.28E-03	0.02	NE	4.26E-03	NE	NE	2.53E-05	NE	NE	7.76E-07	NE	NE	6.56E-04
CGCR	0.13	0.17	4.45E-02	0.03	NE	6.07E-04	0.03	NE	2.78E-03	NE	NE	8.90E-06	NE	NE	9.07E-07	NE	NE	4.83E-04
CSTL	0.20	0.09	2.39E-02	0.10	NE	3.79E-03	0.04	NE	5.11E-03	NE	NE	7.24E-05	NE	NE	2.36E-06	NE	NE	3.78E-07
CSTR	0.21	0.07	2.09E-02	0.11	NE	2.58E-03	0.09	NE	3.26E-03	NE	NE	1.75E-05	NE	NE	3.00E-05	NE	NE	4.22E-04
FA	0.19	0.15	4.06E-02	0.07	NE	1.75E-03	0.08	NE	1.60E-03	0.02	NE	1.03E-05	0.03	NE	5.68E-05	0.02	NE	1.78E-05
FP	0.27	0.06	1.71E-02	0.17	NE	3.51E-03	0.26	NE	6.28E-03	0.02	NE	4.03E-06	0.01	NE	1.90E-05	0.03	NE	2.52E-04
IFOL	0.25	0.20	5.41E-02	0.08	NE	1.69E-03	0.13	NE	3.97E-03	0.01	NE	4.56E-05	0.01	NE	3.28E-05	0.02	NE	8.27E-05
IFOR	0.23	0.18	4.68E-02	0.08	NE	1.63E-03	0.07	NE	9.38E-05	0.02	NE	2.09E-05	0.02	NE	2.43E-05	0.02	NE	7.01E-08
ILFL	0.20	0.19	5.10E-02	0.05	NE	1.30E-03	0.06	NE	1.83E-05	0.00	NE	3.52E-05	0.00	NE	9.71E-06	0.01	NE	2.58E-04
ILFR	0.19	0.19	5.20E-02	0.05	NE	7.41E-04	0.03	NE	7.93E-04	0.01	NE	2.29E-05	0.01	NE	1.07E-05	0.00	NE	7.05E-05
pARCL	0.08	0.16	4.31E-02	0.02	NE	1.22E-03	0.03	NE	2.15E-03	NE	NE	4.53E-05	NE	NE	1.03E-05	NE	NE	1.61E-03

pARCR	0.11	0.17	4.56E-02	0.03	NE	6.09E-04	0.04	NE	2.31E-03	NE	NE	3.03E-05	NE	NE	2.39E-05	NE	NE	4.01E-04
SLFL	0.14	0.12	3.12E-02	0.04	NE	2.14E-03	0.02	NE	2.42E-03	NE	NE	5.75E-05	NE	NE	2.91E-05	NE	NE	5.02E-04
SLFR	0.17	0.12	3.32E-02	0.05	NE	1.27E-03	0.02	NE	4.14E-03	NE	NE	3.10E-05	NE	NE	2.51E-05	NE	NE	1.51E-04

NE = Not estimable. After covariate adjustment, the random effect for site (batch) collapsed to the boundary of the parameter space, resulting in a singular mixed-effects model and preventing ICC estimation. This indicates minimal residual batch effects. ICC was estimated from a linear mixed-effects model with a random intercept for site. ICC (cor.) represents the same intraclass correlation after adjusting for covariates (Age, Age², and Sex). η^2 was derived from the Type III ANOVA of the corresponding linear model, reflecting the proportion of variance explained by site after covariate adjustment. *ICC: intraclass correlation coefficient; GAM: generalized additive models; cGM: cortical grey matter; sWM: superficial white matter; WM: white matter; BA: Brodmann Area; FA: Callosum Forceps Minor; FP: Callosum Forceps Major; ARC: Arcuate; pARC: Posterior Arcuate Fasciculus; ATR: Thalamic Radiation; CGC: Cingulum Cingulate; CST: Corticospinal; IFO: Inferior Fronto-Occipital Fasciculus; ILF: Inferior Longitudinal Fasciculus; SLF: Superior Longitudinal Fasciculus.L: leftl; R: right.*

S. 3.3 Comparison of Polynomial Age-Fitting Model Parameters Before and After Data Harmonization

	Raw									
Region	α	β (age)	β (age2)	β (Sex)	*p* (age)	*p* (age2)	*p* (Sex)	R2 (Adjusted)	Peak Age	SE (Peak Age)
R1										
cGM										
BA6	0.60	4.33E-03	-3.81E-05	1.91E-03	4.81E-110	4.02E-77	1.11E-01	0.77	56.85	0.71
BA4	0.63	5.12E-03	-4.73E-05	1.51E-04	1.75E-100	1.02E-74	9.21E-01	0.71	54.18	0.69
BA9	0.61	3.33E-03	-2.91E-05	1.46E-03	5.45E-102	3.78E-70	1.36E-01	0.76	57.24	0.77
BA3	0.64	4.45E-03	-4.19E-05	1.41E-03	6.97E-82	9.37E-62	3.59E-01	0.62	53.04	0.69
BA1	0.64	4.46E-03	-4.19E-05	-5.24E-04	1.72E-82	7.20E-62	7.32E-01	0.63	53.26	0.76
BA5	0.60	4.24E-03	-3.89E-05	3.54E-03	4.60E-101	2.59E-74	4.88E-03	0.72	54.61	0.66
BA7	0.60	4.31E-03	-3.96E-05	2.08E-03	4.50E-123	8.83E-93	5.67E-02	0.77	54.47	0.58
BA2	0.61	4.12E-03	-3.81E-05	2.33E-03	1.63E-110	4.42E-83	4.11E-02	0.74	54.12	0.58
BA31	0.59	3.81E-03	-3.47E-05	1.28E-03	2.88E-107	1.55E-78	2.33E-01	0.74	55.02	0.64
BA40	0.60	3.78E-03	-3.37E-05	2.28E-03	9.39E-126	2.04E-91	1.53E-02	0.80	56.06	0.62
BA44	0.61	3.58E-03	-3.13E-05	8.93E-04	3.99E-112	3.53E-78	3.60E-01	0.78	57.15	0.80
BA45	0.61	3.78E-03	-3.32E-05	5.94E-04	2.46E-117	1.29E-82	5.50E-01	0.79	57.01	0.80
BA23	0.59	3.73E-03	-3.36E-05	-1.19E-04	7.89E-107	2.81E-77	9.10E-01	0.75	55.46	0.68
BA39	0.60	3.76E-03	-3.35E-05	1.47E-03	1.56E-121	7.53E-88	1.25E-01	0.79	56.11	0.71
BA43	0.61	3.51E-03	-3.13E-05	8.24E-06	7.03E-99	9.02E-70	9.94E-01	0.73	56.11	0.83
BA19	0.61	4.07E-03	-3.78E-05	7.65E-04	4.88E-123	3.45E-94	4.57E-01	0.77	53.85	0.54
BA47	0.60	3.80E-03	-3.31E-05	2.22E-03	2.83E-114	4.46E-79	2.99E-02	0.79	57.58	0.78
BA41	0.61	3.38E-03	-3.04E-05	-1.34E-04	5.00E-83	2.06E-58	9.07E-01	0.67	55.57	0.86
BA30	0.59	3.88E-03	-3.50E-05	1.67E-03	1.11E-100	3.11E-72	1.47E-01	0.73	55.53	0.70
BA22	0.61	3.26E-03	-2.82E-05	8.15E-04	8.21E-118	3.05E-81	3.40E-01	0.80	57.87	0.82
BA42	0.62	4.09E-03	-3.74E-05	1.79E-03	3.73E-90	2.67E-65	1.73E-01	0.68	54.76	0.71
BA21	0.59	3.00E-03	-2.60E-05	1.10E-03	1.28E-104	2.48E-71	2.04E-01	0.77	57.71	0.82
BA38	0.60	2.02E-03	-1.69E-05	4.76E-04	2.84E-67	2.11E-41	5.52E-01	0.67	59.75	1.20
BA37	0.61	3.12E-03	-2.79E-05	8.07E-04	2.26E-105	3.99E-75	3.66E-01	0.75	55.98	0.75
BA20	0.59	2.83E-03	-2.47E-05	1.68E-03	1.81E-97	9.87E-67	4.99E-02	0.74	57.17	0.80
BA32	0.60	2.75E-03	-2.37E-05	1.04E-03	7.09E-86	1.31E-56	2.55E-01	0.72	58.09	0.92
BA24	0.60	2.55E-03	-2.25E-05	6.76E-04	8.49E-77	5.50E-52	4.62E-01	0.66	56.71	0.88
BA10	0.61	3.24E-03	-2.84E-05	1.72E-03	9.81E-91	6.69E-62	9.80E-02	0.72	57.03	0.86
BA25	0.65	-1.82E-04	1.44E-06	4.01E-04	2.98E-01	4.73E-01	7.73E-01	0.00	55.47	7.95
BA11	0.61	3.15E-03	-2.79E-05	2.71E-03	2.12E-102	3.84E-72	3.36E-03	0.75	56.33	0.81
BA46	0.61	3.51E-03	-3.08E-05	1.49E-03	9.28E-108	6.92E-75	1.32E-01	0.77	57.08	0.77
BA17	0.64	4.10E-03	-3.94E-05	-1.14E-03	8.14E-80	8.91E-62	4.27E-01	0.59	52.06	0.65
BA18	0.62	4.32E-03	-4.08E-05	3.13E-04	3.22E-103	1.50E-79	8.03E-01	0.70	52.91	0.57
BA27	0.61	2.93E-03	-2.52E-05	2.84E-03	9.53E-74	1.32E-47	9.46E-03	0.68	58.31	1.06
BA36	0.61	3.03E-03	-2.89E-05	3.73E-03	1.22E-87	1.14E-67	1.92E-04	0.64	52.38	0.63
BA35	0.60	3.14E-03	-2.79E-05	1.25E-03	9.92E-76	1.30E-51	2.75E-01	0.65	56.41	0.95
BA28	0.60	3.53E-03	-3.34E-05	5.78E-03	1.58E-69	3.98E-52	2.67E-05	0.57	52.85	0.74
BA29	0.58	3.83E-03	-3.45E-05	4.68E-04	2.14E-87	6.89E-62	7.10E-01	0.68	55.49	0.78
BA26	0.60	3.65E-03	-3.39E-05	3.14E-05	6.28E-45	6.45E-32	9.87E-01	0.44	53.89	1.12
BA33	0.61	1.48E-03	-1.27E-05	2.82E-04	1.41E-36	2.09E-22	7.44E-01	0.46	58.69	1.95
sWM										
BA6	1.04	3.32E-03	-4.74E-05	2.88E-03	2.59E-18	1.25E-26	3.23E-01	0.30	34.97	1.12
BA4	1.04	3.48E-03	-4.87E-05	1.86E-03	2.31E-19	3.09E-27	5.31E-01	0.29	35.67	1.12
BA9	1.03	3.20E-03	-4.69E-05	5.29E-03	1.14E-16	2.20E-25	7.49E-02	0.31	34.05	1.19
BA3	1.00	3.22E-03	-3.82E-05	3.07E-03	1.13E-15	1.13E-16	3.22E-01	0.12	42.11	1.04
BA1	0.95	5.23E-03	-5.89E-05	3.02E-03	6.22E-34	4.52E-33	3.44E-01	0.24	44.38	0.65
BA5	0.98	4.41E-03	-5.68E-05	5.86E-03	5.09E-23	4.18E-28	8.37E-02	0.24	38.80	0.87
BA7	1.01	4.08E-03	-5.30E-05	3.13E-03	9.59E-23	3.42E-28	3.20E-01	0.24	38.45	0.84
BA2	1.05	3.32E-03	-4.64E-05	-2.77E-04	1.12E-15	4.09E-22	9.31E-01	0.24	35.61	1.18
BA31	1.02	4.14E-03	-5.49E-05	2.84E-03	2.31E-25	1.76E-32	3.45E-01	0.29	37.66	0.82
BA40	0.97	5.00E-03	-6.15E-05	9.39E-03	2.17E-32	1.64E-36	2.83E-03	0.28	40.64	0.65
BA44	1.09	2.02E-03	-3.52E-05	7.97E-05	2.97E-08	1.02E-16	9.78E-01	0.34	28.47	2.04
BA45	1.00	3.99E-03	-5.16E-05	2.65E-03	4.68E-24	1.41E-29	3.74E-01	0.25	38.64	0.78
BA23	1.00	4.66E-03	-5.89E-05	-3.90E-04	1.15E-31	2.06E-37	8.95E-01	0.29	39.48	0.72
BA39	1.03	4.06E-03	-5.38E-05	7.67E-03	1.74E-24	2.28E-31	1.09E-02	0.29	37.68	0.81
BA43	0.99	4.84E-03	-6.00E-05	2.92E-03	1.15E-29	6.11E-34	3.61E-01	0.26	40.30	0.70
BA19	1.02	4.32E-03	-5.56E-05	3.11E-03	2.51E-30	8.19E-37	2.70E-01	0.30	38.86	0.67
BA47	0.98	5.11E-03	-6.15E-05	3.03E-03	5.19E-35	4.55E-38	3.22E-01	0.27	41.51	0.64
BA41	1.07	2.91E-03	-4.27E-05	-3.67E-03	8.74E-15	1.36E-22	2.06E-01	0.29	33.95	1.27
BA30	1.00	4.77E-03	-5.93E-05	1.07E-03	1.77E-30	5.10E-35	7.29E-01	0.27	40.19	0.74
BA22	0.99	5.56E-03	-6.41E-05	6.61E-04	5.22E-42	1.79E-42	8.24E-01	0.30	43.40	0.60
BA42	1.09	2.25E-03	-3.78E-05	-2.29E-03	1.58E-09	4.07E-18	4.32E-01	0.33	29.68	1.77
BA21	0.93	6.82E-03	-7.61E-05	7.11E-03	5.77E-56	8.08E-54	1.99E-02	0.38	44.82	0.56
BA38	0.84	6.65E-03	-6.79E-05	5.80E-03	1.03E-50	2.62E-42	6.67E-02	0.39	48.98	0.79
BA37	0.99	5.24E-03	-6.34E-05	6.75E-03	1.64E-37	4.58E-41	2.50E-02	0.30	41.33	0.60
BA20	0.90	6.32E-03	-6.94E-05	3.82E-03	2.89E-49	4.20E-46	2.13E-01	0.34	45.57	0.62
BA32	1.07	2.83E-03	-4.25E-05	1.89E-03	2.76E-13	3.73E-21	5.28E-01	0.29	33.12	1.39
BA24	1.05	2.73E-03	-3.92E-05	9.07E-03	6.53E-13	4.60E-19	2.15E-03	0.24	34.66	1.35
BA10	1.03	3.18E-03	-4.48E-05	4.65E-03	6.24E-17	4.58E-24	1.13E-01	0.27	35.46	1.13
BA25	0.92	4.04E-03	-5.30E-05	3.37E-03	6.88E-15	9.34E-19	4.00E-01	0.17	38.05	1.21
BA11	0.98	4.76E-03	-5.78E-05	6.58E-03	9.34E-36	2.58E-39	1.96E-02	0.29	41.24	0.66
BA46	1.06	2.74E-03	-4.16E-05	9.77E-04	2.07E-12	4.67E-20	7.47E-01	0.28	32.82	1.35
BA17	0.95	3.47E-03	-4.16E-05	3.19E-03	5.20E-19	1.21E-20	2.85E-01	0.15	41.61	0.85
BA18	0.98	4.21E-03	-5.10E-05	3.46E-03	6.76E-28	1.17E-30	2.31E-01	0.23	41.28	0.68
BA27	0.89	3.39E-03	-3.12E-05	5.38E-03	1.38E-11	4.37E-08	1.68E-01	0.14	54.80	3.09
BA36	0.87	4.46E-03	-4.55E-05	-4.04E-03	7.18E-33	3.94E-27	1.45E-01	0.27	49.06	1.01
BA35	0.97	3.58E-03	-4.16E-05	4.82E-03	5.13E-22	1.55E-22	8.80E-02	0.17	43.09	0.89
BA28	0.93	4.48E-03	-4.71E-05	-3.36E-03	1.46E-29	1.23E-25	2.58E-01	0.23	47.60	0.98
BA29	0.88	6.16E-03	-6.69E-05	2.79E-04	7.00E-34	4.95E-31	9.41E-01	0.25	46.09	0.75
BA26	0.89	6.23E-03	-6.62E-05	-2.81E-03	1.69E-31	8.93E-28	4.80E-01	0.24	47.13	0.89
BA33	0.97	3.65E-03	-4.59E-05	4.38E-03	2.20E-15	4.48E-18	2.18E-01	0.15	39.71	1.06
WM Bundles										
ARCR	1.12	3.20E-03	-4.26E-05	4.19E-03	2.10E-16	2.96E-21	1.62E-01	0.20	37.53	1.06
ATRL	1.13	3.74E-03	-5.38E-05	3.52E-03	6.24E-19	6.81E-28	2.74E-01	0.33	34.71	1.02
ATRR	1.13	3.54E-03	-4.97E-05	4.62E-03	1.67E-17	8.95E-25	1.47E-01	0.28	35.59	1.04
CGCL	1.09	3.75E-03	-5.00E-05	7.43E-03	4.40E-19	1.09E-24	2.08E-02	0.24	37.54	0.97
CGCR	1.07	4.52E-03	-5.68E-05	9.93E-03	7.46E-27	1.95E-31	1.74E-03	0.26	39.78	0.76
CSTL	1.13	1.30E-03	-2.52E-05	3.27E-03	1.87E-04	3.59E-10	2.32E-01	0.28	25.19	3.55
CSTR	1.11	2.48E-03	-3.63E-05	3.71E-03	3.49E-12	1.70E-18	1.80E-01	0.24	34.07	1.44
FA	1.19	2.24E-03	-3.91E-05	2.48E-03	1.46E-07	2.91E-15	4.56E-01	0.32	28.41	2.15
FP	1.15	1.77E-03	-3.29E-05	-3.58E-03	6.54E-06	6.26E-13	2.46E-01	0.32	26.70	2.59
IFOL	1.14	2.65E-03	-4.39E-05	5.13E-04	1.58E-11	1.09E-21	8.66E-01	0.38	29.97	1.55
IFOR	1.11	3.85E-03	-5.38E-05	-5.44E-04	3.73E-21	1.03E-29	8.61E-01	0.32	35.70	0.89

	Combat									
Region	α	β (age)	β (age2)	β (Sex)	*p* (age)	*p* (age2)	*p* (Sex)	R2 (Adjusted)	Peak Age	SE (Peak Age)
R1										
cGM										
BA6	0.65	2.12E-03	-1.77E-05	-3.86E-04	4.15E-40	5.62E-24	5.08E-01	0.50	59.90	1.76
BA4	0.68	2.77E-03	-2.47E-05	3.35E-04	4.95E-40	1.42E-26	6.62E-01	0.44	56.17	1.55
BA9	0.64	1.83E-03	-1.57E-05	-6.24E-04	1.41E-39	2.20E-24	2.20E-01	0.48	58.66	1.68
BA3	0.69	1.84E-03	-1.65E-05	-2.41E-04	6.01E-26	3.16E-17	7.15E-01	0.31	56.10	2.12
BA1	0.69	2.19E-03	-1.98E-05	6.52E-04	2.50E-29	9.67E-20	3.72E-01	0.34	55.59	1.94
BA5	0.66	1.65E-03	-1.53E-05	-1.29E-03	9.80E-27	6.99E-19	2.69E-02	0.30	53.94	1.62
BA7	0.66	1.90E-03	-1.75E-05	-7.31E-04	3.62E-39	1.22E-27	1.69E-01	0.40	54.23	1.31
BA2	0.66	1.90E-03	-1.74E-05	-8.90E-04	1.18E-35	8.73E-25	1.13E-01	0.38	54.57	1.42
BA31	0.64	1.51E-03	-1.31E-05	-3.83E-04	8.52E-33	8.07E-21	4.15E-01	0.41	57.69	1.98
BA40	0.64	1.78E-03	-1.55E-05	-7.92E-04	6.35E-43	1.81E-27	9.17E-02	0.49	57.49	1.46
BA44	0.65	1.83E-03	-1.50E-05	-2.78E-04	2.16E-41	5.53E-24	5.74E-01	0.53	60.97	2.10
BA45	0.65	1.99E-03	-1.66E-05	-1.37E-04	8.59E-44	2.89E-26	7.92E-01	0.53	59.82	1.83
BA23	0.64	1.49E-03	-1.27E-05	3.43E-04	2.76E-32	9.05E-20	4.64E-01	0.42	58.77	2.12
BA39	0.65	1.67E-03	-1.44E-05	-4.46E-04	6.48E-40	3.98E-25	3.35E-01	0.47	58.08	1.76
BA43	0.65	1.82E-03	-1.53E-05	1.89E-04	3.03E-35	3.51E-21	7.27E-01	0.46	59.54	2.08
BA19	0.66	1.70E-03	-1.58E-05	-3.66E-05	5.20E-38	4.03E-27	9.40E-01	0.38	53.72	1.31
BA47	0.64	1.99E-03	-1.66E-05	-7.20E-04	2.42E-43	9.65E-26	1.69E-01	0.53	60.12	1.78
BA41	0.65	1.57E-03	-1.30E-05	2.71E-04	1.01E-27	5.12E-16	6.16E-01	0.40	60.82	2.75
BA30	0.64	1.49E-03	-1.26E-05	-4.75E-04	6.59E-28	1.42E-16	3.54E-01	0.39	59.76	2.38
BA22	0.64	1.70E-03	-1.38E-05	-1.35E-04	2.55E-44	4.20E-25	7.60E-01	0.56	61.83	1.97
BA42	0.67	1.93E-03	-1.64E-05	-5.86E-04	1.48E-30	1.71E-18	3.50E-01	0.41	59.06	2.06
BA21	0.63	1.40E-03	-1.15E-05	-2.49E-04	4.16E-34	1.69E-19	5.57E-01	0.47	61.09	2.25
BA38	0.62	1.04E-03	-7.85E-06	-1.16E-04	3.56E-23	1.62E-11	7.70E-01	0.42	66.66	3.91
BA37	0.64	1.52E-03	-1.31E-05	-1.04E-04	1.45E-35	3.05E-22	8.18E-01	0.44	58.17	1.90
BA20	0.62	1.12E-03	-8.72E-06	-3.89E-04	6.18E-28	1.29E-14	3.12E-01	0.46	64.45	3.09
BA32	0.63	1.42E-03	-1.14E-05	-2.02E-04	1.74E-30	6.80E-17	6.60E-01	0.45	62.38	2.60
BA24	0.63	1.14E-03	-9.16E-06	-1.89E-04	1.15E-23	5.71E-13	6.63E-01	0.39	62.73	2.96
BA10	0.64	1.76E-03	-1.55E-05	-7.46E-04	4.24E-35	8.34E-23	1.57E-01	0.42	56.70	1.52
BA25	0.62	1.45E-03	-1.28E-05	-1.21E-03	3.26E-24	1.12E-15	2.69E-02	0.32	56.82	2.05
BA11	0.64	1.69E-03	-1.47E-05	-1.13E-03	1.28E-38	1.08E-24	1.76E-02	0.46	57.54	1.68
BA46	0.65	1.87E-03	-1.59E-05	-6.06E-04	3.86E-40	1.40E-24	2.39E-01	0.49	58.95	1.75
BA17	0.69	1.47E-03	-1.40E-05	1.03E-03	2.52E-20	7.36E-15	8.99E-02	0.21	52.93	1.86
BA18	0.68	1.69E-03	-1.59E-05	2.62E-04	2.43E-29	2.59E-21	6.42E-01	0.30	53.14	1.53
BA27	0.65	1.31E-03	-9.97E-06	-1.19E-03	4.21E-22	4.48E-11	2.16E-02	0.41	66.68	4.35
BA36	0.64	1.48E-03	-1.42E-05	-1.57E-03	1.88E-29	4.91E-22	1.50E-03	0.30	52.11	1.29
BA35	0.64	1.25E-03	-9.83E-06	-2.72E-04	8.49E-21	4.34E-11	5.95E-01	0.36	64.28	4.08
BA28	0.64	1.74E-03	-1.65E-05	-2.46E-03	5.40E-22	5.39E-16	3.89E-04	0.25	52.97	1.50
BA29	0.63	1.53E-03	-1.31E-05	1.41E-04	4.09E-23	3.71E-14	8.10E-01	0.32	58.65	2.39
BA26	0.64	1.57E-03	-1.42E-05	3.18E-04	2.14E-11	8.22E-08	7.29E-01	0.14	55.59	3.30
BA33	0.62	9.28E-04	-7.15E-06	-1.35E-04	1.44E-16	1.29E-08	7.55E-01	0.32	65.72	5.24
sWM										
BA6	1.08	1.32E-03	-2.68E-05	-9.51E-04	2.08E-04	6.32E-11	4.98E-01	0.32	24.07	3.58
BA4	1.08	1.29E-03	-2.61E-05	-3.65E-04	2.43E-04	1.39E-10	7.93E-01	0.30	24.22	3.60
BA9	1.06	1.87E-03	-3.26E-05	-2.27E-03	4.68E-07	3.27E-14	1.21E-01	0.30	28.37	2.38
BA3	1.05	5.48E-04	-1.02E-05	-6.27E-04	8.90E-02	5.76E-03	6.25E-01	0.06	25.24	7.78
BA1	1.02	1.79E-03	-2.42E-05	-5.67E-04	4.87E-07	3.19E-09	6.85E-01	0.09	36.92	1.95
BA5	1.03	1.57E-03	-2.75E-05	-2.01E-03	7.94E-05	2.05E-09	2.03E-01	0.21	28.02	3.41
BA7	1.05	1.68E-03	-2.77E-05	-1.01E-03	4.87E-06	6.93E-11	4.85E-01	0.20	30.01	2.82
BA2	1.09	1.02E-03	-2.17E-05	8.14E-04	4.86E-03	1.63E-07	5.70E-01	0.25	22.20	5.27
BA31	1.07	1.60E-03	-2.95E-05	-6.63E-04	1.58E-05	6.11E-12	6.50E-01	0.29	26.82	2.90
BA40	1.03	2.25E-03	-3.33E-05	-3.73E-03	4.18E-09	4.05E-14	1.30E-02	0.20	33.52	1.71
BA44	1.11	9.94E-04	-2.34E-05	2.37E-04	3.77E-03	3.81E-09	8.62E-01	0.33	20.68	4.85
BA45	1.04	2.23E-03	-3.30E-05	-8.22E-04	2.41E-09	1.59E-14	5.75E-01	0.20	33.64	1.62
BA23	1.05	1.98E-03	-3.33E-05	1.10E-03	2.34E-07	5.21E-14	4.66E-01	0.27	29.45	2.32
BA39	1.08	1.63E-03	-2.88E-05	-2.94E-03	6.34E-06	4.84E-12	3.90E-02	0.27	28.00	2.64
BA43	1.04	2.38E-03	-3.52E-05	-6.74E-04	3.87E-09	3.93E-14	6.69E-01	0.19	33.65	1.71
BA19	1.08	1.61E-03	-2.81E-05	-8.21E-04	1.66E-06	4.88E-13	5.36E-01	0.28	28.27	2.69
BA47	1.02	2.99E-03	-4.04E-05	-1.03E-03	7.28E-14	1.82E-18	5.05E-01	0.18	37.00	1.12
BA41	1.10	1.22E-03	-2.56E-05	2.41E-03	7.23E-04	9.07E-10	9.36E-02	0.30	23.25	3.88
BA30	1.05	2.01E-03	-3.29E-05	3.57E-04	4.84E-07	9.21E-13	8.21E-01	0.24	30.10	2.63
BA22	1.05	2.93E-03	-3.85E-05	4.25E-04	5.07E-14	8.67E-18	7.78E-01	0.16	38.05	1.11
BA42	1.11	9.91E-04	-2.30E-05	1.43E-03	3.55E-03	4.18E-09	2.91E-01	0.33	20.69	4.49
BA21	1.00	3.32E-03	-4.32E-05	-2.34E-03	4.46E-16	4.26E-20	1.39E-01	0.18	38.38	1.03
BA38	0.90	3.78E-03	-4.18E-05	-1.95E-03	1.22E-18	1.01E-17	2.36E-01	0.14	45.20	1.08
BA37	1.04	2.66E-03	-3.85E-05	-2.50E-03	1.98E-11	3.65E-17	1.05E-01	0.22	34.37	1.48
BA20	0.97	3.36E-03	-4.17E-05	-8.73E-04	3.26E-16	1.04E-18	5.82E-01	0.14	40.23	1.00
BA32	1.09	1.67E-03	-3.04E-05	-7.25E-04	8.14E-06	2.33E-12	6.24E-01	0.29	27.39	2.57
BA24	1.08	1.49E-03	-2.65E-05	-4.18E-03	5.85E-05	4.95E-10	4.37E-03	0.23	27.51	3.12
BA10	1.05	2.01E-03	-3.32E-05	-2.04E-03	8.57E-08	2.29E-14	1.67E-01	0.26	30.12	2.09
BA25	0.93	3.74E-03	-4.97E-05	-1.65E-03	4.73E-13	6.54E-17	4.13E-01	0.16	37.55	1.17
BA11	1.02	2.84E-03	-3.98E-05	-2.73E-03	7.13E-14	1.03E-19	6.34E-02	0.22	35.53	1.17
BA46	1.08	1.56E-03	-2.77E-05	-1.46E-04	1.85E-05	3.61E-11	9.19E-01	0.25	27.68	2.56
BA17	1.00	9.15E-04	-1.50E-05	-8.85E-04	5.40E-03	6.60E-05	4.98E-01	0.08	29.86	3.16
BA18	1.04	1.40E-03	-2.20E-05	-1.03E-03	1.50E-05	2.93E-09	4.17E-01	0.15	31.25	2.76
BA27	0.90	2.70E-03	-2.34E-05	-2.27E-03	3.22E-08	2.44E-05	2.36E-01	0.12	58.52	4.82
BA36	0.90	2.95E-03	-3.06E-05	2.56E-03	4.58E-16	1.07E-13	6.82E-02	0.13	48.41	1.42
BA35	0.99	2.31E-03	-2.81E-05	-2.09E-03	1.05E-10	5.92E-12	1.34E-01	0.09	40.96	1.29
BA28	0.96	3.00E-03	-3.24E-05	1.99E-03	7.98E-15	1.36E-13	1.84E-01	0.11	46.26	1.34
BA29	0.94	3.25E-03	-3.96E-05	9.85E-04	5.17E-11	2.77E-12	6.09E-01	0.09	40.94	1.28
BA26	0.95	3.58E-03	-4.19E-05	2.34E-03	7.76E-12	2.15E-12	2.51E-01	0.09	42.72	1.21
BA33	0.99	2.74E-03	-3.65E-05	-2.08E-03	2.09E-09	3.23E-12	2.47E-01	0.12	37.41	1.54
WM Bundles										
ARCR	1.18	8.46E-04	-2.12E-05	-1.56E-03	2.60E-02	1.49E-06	2.97E-01	0.27	18.55	6.42
ATRL	1.17	1.92E-03	-3.60E-05	-1.30E-03	4.22E-06	1.32E-13	4.24E-01	0.33	26.28	2.54
ATRR	1.17	1.73E-03	-3.27E-05	-1.78E-03	3.12E-05	1.29E-11	2.73E-01	0.30	26.15	2.91
CGCL	1.14	1.57E-03	-2.91E-05	-3.20E-03	1.46E-04	1.25E-09	4.93E-02	0.24	26.44	3.36
CGCR	1.14	1.56E-03	-2.93E-05	-4.27E-03	1.25E-04	4.73E-10	7.46E-03	0.26	26.05	3.24
CSTL	1.15	5.40E-04	-1.86E-05	-1.38E-03	1.22E-01	4.30E-06	3.18E-01	0.33	13.55	5.86
CSTR	1.16	3.80E-04	-1.73E-05	-1.30E-03	2.76E-01	1.71E-05	3.45E-01	0.35	9.95	6.55
FA	1.21	1.27E-03	-2.96E-05	-1.04E-03	3.02E-03	2.81E-09	5.37E-01	0.33	20.95	4.21
FP	1.19	1.02E-05	-1.75E-05	2.60E-03	9.79E-01	1.11E-04	9.36E-02	0.43	-1.02	8.37
IFOL	1.17	1.26E-03	-3.14E-05	1.68E-04	1.43E-03	9.89E-12	9.14E-01	0.43	19.44	3.72
IFOR	1.17	1.17E-03	-2.93E-05	9.54E-04	3.32E-03	2.45E-10	5.42E-01	0.39	19.25	4.11

	GAM									
Region	α	β (age)	β (age2)	β (Sex)	*p* (age)	*p* (age2)	*p* (Sex)	R2 (Adjusted)	Peak Age	SE (Peak Age)
R1										
cGM										
BA6	0.63	3.06E-03	-2.62E-05	1.45E-03	1.18E-87	5.76E-58	1.52E-01	0.72	58.30	1.04
BA4	0.65	4.31E-03	-3.87E-05	-6.69E-05	9.55E-97	2.66E-69	9.60E-01	0.71	55.65	0.86
BA9	0.62	2.85E-03	-2.51E-05	1.60E-03	1.14E-94	2.52E-65	7.23E-02	0.73	56.99	0.84
BA3	0.67	2.86E-03	-2.59E-05	9.95E-04	9.48E-61	2.27E-42	4.14E-01	0.56	55.27	1.14
BA1	0.66	3.40E-03	-3.09E-05	-7.94E-04	6.03E-69	1.02E-48	5.50E-01	0.59	55.10	1.06
BA5	0.64	2.61E-03	-2.41E-05	2.99E-03	7.49E-69	6.59E-50	3.49E-03	0.58	54.20	0.93
BA7	0.63	3.10E-03	-2.86E-05	1.74E-03	1.05E-100	4.49E-75	6.06E-02	0.71	54.29	0.74
BA2	0.64	3.07E-03	-2.82E-05	2.16E-03	1.58E-93	6.63E-69	2.55E-02	0.69	54.47	0.74
BA31	0.63	2.34E-03	-2.08E-05	8.25E-04	7.98E-78	1.19E-53	3.26E-01	0.65	56.32	1.01
BA40	0.63	2.75E-03	-2.44E-05	2.01E-03	1.46E-101	6.07E-72	1.35E-02	0.74	56.27	0.79
BA44	0.63	2.83E-03	-2.43E-05	8.73E-04	4.45E-97	7.01E-65	3.14E-01	0.75	58.33	1.04
BA45	0.63	3.16E-03	-2.75E-05	6.65E-04	1.21E-102	1.78E-70	4.74E-01	0.76	57.53	0.94
BA23	0.62	2.33E-03	-2.04E-05	-5.75E-04	5.34E-75	1.33E-50	5.03E-01	0.65	56.99	1.11
BA39	0.63	2.64E-03	-2.34E-05	1.07E-03	1.73E-91	1.08E-63	2.03E-01	0.71	56.53	0.94
BA43	0.63	2.91E-03	-2.54E-05	3.66E-05	2.30E-83	2.14E-56	9.71E-01	0.69	57.23	1.02
BA19	0.64	2.77E-03	-2.57E-05	2.37E-04	4.52E-94	2.38E-70	7.85E-01	0.68	53.94	0.71
BA47	0.62	3.05E-03	-2.63E-05	2.13E-03	8.17E-95	7.40E-64	2.52E-02	0.74	57.94	0.93
BA41	0.63	2.38E-03	-2.04E-05	-2.26E-04	1.60E-63	1.40E-40	8.18E-01	0.62	58.48	1.38
BA30	0.62	2.35E-03	-2.05E-05	1.08E-03	3.18E-62	5.16E-41	2.70E-01	0.60	57.29	1.21
BA22	0.62	2.65E-03	-2.25E-05	6.26E-04	3.27E-95	1.22E-62	4.47E-01	0.75	58.87	1.03
BA42	0.65	2.92E-03	-2.56E-05	1.48E-03	2.81E-66	5.02E-44	2.08E-01	0.62	57.16	1.10
BA21	0.61	2.14E-03	-1.84E-05	8.43E-04	9.64E-76	3.36E-49	2.83E-01	0.68	58.41	1.15
BA38	0.61	1.52E-03	-1.24E-05	4.63E-04	4.23E-51	2.04E-29	5.22E-01	0.60	61.71	1.71
BA37	0.62	2.43E-03	-2.14E-05	4.68E-04	8.45E-86	5.75E-59	5.66E-01	0.69	56.76	0.98
BA20	0.61	1.64E-03	-1.35E-05	1.18E-03	2.33E-55	5.68E-33	1.11E-01	0.61	60.61	1.62
BA32	0.62	2.13E-03	-1.80E-05	1.08E-03	1.68E-66	6.50E-42	2.03E-01	0.65	59.08	1.24
BA24	0.62	1.69E-03	-1.42E-05	6.66E-04	1.75E-53	1.11E-32	3.94E-01	0.59	59.63	1.51
BA10	0.63	2.74E-03	-2.47E-05	1.85E-03	4.77E-78	6.58E-55	5.99E-02	0.65	55.59	0.90
BA25	0.59	2.00E-03	-1.76E-05	2.30E-03	4.97E-59	3.29E-39	8.12E-03	0.58	56.89	1.12
BA11	0.62	2.60E-03	-2.31E-05	2.75E-03	1.18E-80	7.02E-56	2.54E-03	0.67	56.25	0.96
BA46	0.63	2.78E-03	-2.43E-05	1.53E-03	2.11E-88	6.80E-60	9.48E-02	0.71	57.42	0.95
BA17	0.67	2.30E-03	-2.16E-05	-1.75E-03	5.64E-47	2.17E-34	1.30E-01	0.43	53.23	1.13
BA18	0.66	2.74E-03	-2.56E-05	-2.72E-04	2.78E-73	2.03E-54	7.91E-01	0.58	53.51	0.85
BA27	0.64	1.89E-03	-1.53E-05	2.59E-03	1.14E-41	9.63E-24	1.13E-02	0.54	61.68	2.04
BA36	0.63	2.30E-03	-2.17E-05	3.48E-03	1.12E-74	2.86E-56	4.60E-05	0.59	53.06	0.79
BA35	0.63	1.81E-03	-1.51E-05	7.00E-04	3.72E-41	1.78E-24	4.77E-01	0.51	60.16	2.00
BA28	0.62	2.65E-03	-2.48E-05	5.33E-03	8.24E-48	7.15E-35	5.41E-05	0.46	53.36	0.99
BA29	0.62	2.32E-03	-2.04E-05	-1.24E-04	9.60E-52	5.00E-34	9.10E-01	0.53	56.97	1.36
BA26	0.63	2.40E-03	-2.18E-05	-4.48E-04	4.25E-26	9.54E-18	7.94E-01	0.30	55.23	1.83
BA33	0.61	1.32E-03	-1.08E-05	5.38E-04	9.22E-32	3.22E-18	5.22E-01	0.45	61.23	2.48
sWM										
BA6	1.05	2.63E-03	-3.87E-05	2.41E-03	1.48E-14	1.80E-22	3.64E-01	0.29	33.92	1.33
BA4	1.05	2.42E-03	-3.63E-05	1.31E-03	2.98E-13	4.76E-21	6.12E-01	0.28	33.27	1.56
BA9	1.01	3.55E-03	-4.78E-05	5.13E-03	6.41E-23	2.22E-30	6.17E-02	0.29	37.10	0.90
BA3	1.04	9.07E-04	-1.34E-05	1.96E-03	3.13E-03	1.36E-04	4.22E-01	0.05	33.40	3.29
BA1	0.99	2.92E-03	-3.43E-05	1.80E-03	5.43E-18	5.26E-19	4.87E-01	0.14	42.50	1.00
BA5	0.99	2.96E-03	-4.01E-05	4.81E-03	6.25E-15	3.90E-20	1.02E-01	0.21	36.71	1.25
BA7	1.01	3.02E-03	-3.98E-05	2.44E-03	5.09E-17	7.27E-22	3.79E-01	0.20	37.83	1.09
BA2	1.06	1.93E-03	-3.00E-05	-1.15E-03	4.48E-08	1.33E-13	6.77E-01	0.22	31.78	2.15
BA31	1.03	3.19E-03	-4.40E-05	1.92E-03	4.43E-18	5.53E-25	4.98E-01	0.26	36.22	1.10
BA40	0.98	4.07E-03	-4.99E-05	8.29E-03	1.91E-27	4.06E-31	3.45E-03	0.24	40.70	0.75
BA44	1.07	2.16E-03	-3.39E-05	-7.64E-05	1.27E-10	3.15E-18	9.77E-01	0.29	31.75	1.66
BA45	0.99	4.05E-03	-4.98E-05	2.46E-03	1.13E-27	1.96E-31	3.80E-01	0.24	40.66	0.74
BA23	1.00	4.19E-03	-5.35E-05	-1.28E-03	4.44E-27	1.03E-32	6.62E-01	0.26	39.18	0.79
BA39	1.03	3.15E-03	-4.26E-05	6.67E-03	1.66E-18	8.05E-25	1.58E-02	0.25	36.88	1.04
BA43	0.99	4.38E-03	-5.35E-05	2.38E-03	3.06E-27	1.23E-30	4.35E-01	0.23	40.91	0.76
BA19	1.03	3.19E-03	-4.25E-05	2.18E-03	1.50E-21	5.27E-28	3.95E-01	0.26	37.57	0.93
BA47	0.96	5.41E-03	-6.23E-05	3.03E-03	8.48E-43	1.40E-43	2.91E-01	0.30	43.40	0.58
BA41	1.06	2.74E-03	-3.94E-05	-4.15E-03	2.60E-14	2.87E-21	1.37E-01	0.26	34.66	1.30
BA30	1.01	4.04E-03	-5.14E-05	1.38E-04	1.42E-23	1.81E-28	9.64E-01	0.23	39.22	0.89
BA22	0.99	5.21E-03	-5.93E-05	5.76E-05	2.05E-39	2.32E-39	9.84E-01	0.28	43.92	0.64
BA42	1.07	2.21E-03	-3.43E-05	-2.40E-03	9.59E-11	3.45E-18	3.69E-01	0.28	32.01	1.65
BA21	0.94	5.94E-03	-6.71E-05	6.06E-03	1.44E-45	5.60E-45	4.59E-02	0.32	44.24	0.61
BA38	0.85	6.28E-03	-6.48E-05	5.24E-03	4.72E-47	3.81E-40	9.56E-02	0.36	48.45	0.79
BA37	0.99	4.89E-03	-5.88E-05	5.94E-03	2.46E-34	1.43E-37	4.57E-02	0.27	41.56	0.66
BA20	0.91	5.89E-03	-6.47E-05	3.14E-03	2.81E-44	7.98E-42	3.05E-01	0.31	45.47	0.65
BA32	1.04	3.24E-03	-4.45E-05	1.92E-03	1.87E-18	2.25E-25	4.98E-01	0.26	36.33	1.06
BA24	1.03	3.09E-03	-4.12E-05	9.11E-03	4.71E-17	3.21E-22	1.42E-03	0.22	37.50	1.07
BA10	1.01	3.84E-03	-4.97E-05	4.73E-03	3.92E-25	3.40E-31	9.25E-02	0.27	38.57	0.82
BA25	0.85	6.82E-03	-7.82E-05	4.62E-03	2.64E-39	8.75E-40	2.26E-01	0.28	43.55	0.64
BA11	0.96	5.30E-03	-6.23E-05	6.49E-03	1.31E-42	7.77E-45	2.16E-02	0.31	42.52	0.60
BA46	1.03	3.02E-03	-4.13E-05	9.69E-04	2.86E-17	1.57E-23	7.25E-01	0.24	36.55	1.03
BA17	0.98	1.71E-03	-2.23E-05	2.03E-03	9.60E-08	1.37E-09	4.21E-01	0.08	38.34	1.74
BA18	1.00	2.61E-03	-3.29E-05	2.49E-03	2.52E-17	1.41E-20	2.94E-01	0.17	39.62	1.02
BA27	0.86	4.36E-03	-3.86E-05	5.30E-03	7.56E-18	1.22E-11	1.75E-01	0.24	56.75	2.54
BA36	0.86	4.82E-03	-4.77E-05	-4.21E-03	4.19E-39	5.62E-31	1.20E-01	0.33	50.58	1.01
BA35	0.94	4.20E-03	-4.54E-05	4.90E-03	1.17E-31	5.86E-29	6.75E-02	0.23	46.31	0.83
BA28	0.91	4.92E-03	-4.99E-05	-3.18E-03	1.70E-37	6.10E-31	2.62E-01	0.30	49.31	0.97
BA29	0.88	5.88E-03	-6.36E-05	-4.46E-04	6.91E-32	3.21E-29	9.05E-01	0.23	46.25	0.76
BA26	0.89	6.15E-03	-6.54E-05	-3.39E-03	2.53E-31	6.33E-28	3.91E-01	0.23	47.02	0.86
BA33	0.94	4.91E-03	-5.62E-05	5.34E-03	1.53E-26	8.17E-27	1.25E-01	0.20	43.64	0.81
WM Bundles										
ARCR	1.16	1.83E-03	-2.97E-05	3.57E-03	1.09E-06	7.52E-12	2.25E-01	0.22	30.52	2.28
ATRL	1.12	3.84E-03	-5.35E-05	3.63E-03	4.62E-20	5.38E-28	2.54E-01	0.30	35.87	0.97
ATRR	1.13	3.41E-03	-4.78E-05	4.58E-03	3.02E-16	5.92E-23	1.53E-01	0.26	35.62	1.10
CGCL	1.10	3.11E-03	-4.31E-05	7.14E-03	2.11E-14	5.90E-20	2.31E-02	0.22	36.01	1.19
CGCR	1.10	3.16E-03	-4.37E-05	9.31E-03	2.64E-15	3.53E-21	2.57E-03	0.24	36.06	1.15
CSTL	1.13	1.20E-03	-2.40E-05	3.27E-03	3.67E-04	7.26E-10	2.18E-01	0.28	24.44	3.64
CSTR	1.15	1.03E-03	-2.28E-05	3.07E-03	2.23E-03	5.59E-09	2.51E-01	0.30	21.99	4.36
FA	1.17	2.71E-03	-4.25E-05	2.78E-03	1.60E-10	6.43E-18	3.98E-01	0.28	31.74	1.62
FP	1.18	6.33E-04	-2.24E-05	-4.17E-03	1.04E-01	6.62E-07	1.78E-01	0.37	12.87	7.43
IFOL	1.13	2.78E-03	-4.48E-05	6.08E-04	1.78E-12	3.04E-22	8.42E-01	0.36	30.99	1.46
IFOR	1.14	2.57E-03	-4.16E-05	-1.16E-03	9.87E-11	2.39E-19	7.06E-01	0.33	30.72	1.59

	B-spline									
Region	α	β (age)	β (age2)	β (Sex)	*p* (age)	*p* (age2)	*p* (Sex)	R2 (Adjusted)	Peak Age	SE (Peak Age)
R1										
cGM										
BA6	0.64	2.89E-03	-2.47E-05	1.07E-03	7.91E-82	6.85E-53	2.84E-01	0.71	58.66	1.13
BA4	0.68	2.82E-03	-2.41E-05	-8.51E-05	8.04E-52	2.83E-32	9.49E-01	0.57	58.67	1.71
BA9	0.62	2.80E-03	-2.48E-05	9.62E-04	3.94E-98	1.43E-68	2.56E-01	0.73	56.45	0.83
BA3	0.67	2.80E-03	-2.53E-05	6.46E-04	8.42E-61	5.03E-42	5.86E-01	0.56	55.37	1.19
BA1	0.66	3.41E-03	-3.09E-05	-1.01E-03	7.08E-75	6.85E-53	4.22E-01	0.63	55.13	1.07
BA5	0.64	2.50E-03	-2.32E-05	2.66E-03	2.02E-63	5.40E-46	9.69E-03	0.56	53.79	0.97
BA7	0.64	3.04E-03	-2.82E-05	1.17E-03	4.41E-97	1.38E-72	2.08E-01	0.69	53.85	0.73
BA2	0.63	3.59E-03	-3.34E-05	1.46E-03	1.18E-112	9.80E-86	1.33E-01	0.74	53.73	0.62
BA31	0.63	2.09E-03	-1.83E-05	6.74E-04	7.20E-73	1.33E-48	3.89E-01	0.65	57.11	1.18
BA40	0.64	2.22E-03	-1.93E-05	1.46E-03	6.54E-76	4.83E-50	7.19E-02	0.67	57.65	1.12
BA44	0.64	2.24E-03	-1.84E-05	4.87E-04	1.52E-71	6.10E-43	5.65E-01	0.70	60.94	1.59
BA45	0.64	2.43E-03	-2.03E-05	2.83E-04	5.13E-74	1.16E-45	7.53E-01	0.70	60.04	1.42
BA23	0.63	1.99E-03	-1.70E-05	-6.89E-04	9.09E-65	3.91E-41	3.93E-01	0.63	58.52	1.42
BA39	0.64	2.23E-03	-1.95E-05	7.55E-04	2.17E-74	1.87E-49	3.60E-01	0.66	57.28	1.20
BA43	0.63	2.58E-03	-2.25E-05	-2.79E-04	2.58E-71	4.72E-47	7.76E-01	0.65	57.43	1.20
BA19	0.66	2.18E-03	-1.99E-05	-1.42E-04	7.34E-68	4.85E-48	8.68E-01	0.59	54.76	0.98
BA47	0.64	2.24E-03	-1.83E-05	1.51E-03	4.85E-60	2.53E-35	1.12E-01	0.65	61.11	1.62
BA41	0.65	1.76E-03	-1.43E-05	-5.32E-04	5.19E-41	2.87E-23	5.78E-01	0.54	61.64	2.32
BA30	0.63	1.96E-03	-1.68E-05	8.52E-04	1.55E-43	4.80E-27	4.05E-01	0.51	58.41	1.57
BA22	0.63	2.30E-03	-1.92E-05	3.93E-04	2.56E-81	1.77E-50	6.23E-01	0.73	60.20	1.31
BA42	0.66	2.39E-03	-2.06E-05	1.26E-03	2.32E-46	4.67E-29	2.94E-01	0.53	58.22	1.56
BA21	0.62	1.74E-03	-1.45E-05	4.61E-04	3.44E-57	1.71E-34	5.47E-01	0.62	60.03	1.63
BA38	0.62	1.17E-03	-8.79E-06	4.63E-04	1.26E-31	1.87E-15	5.34E-01	0.52	67.10	3.36
BA37	0.64	1.77E-03	-1.51E-05	3.90E-04	2.00E-57	4.89E-36	6.15E-01	0.60	58.62	1.51
BA20	0.62	1.49E-03	-1.21E-05	1.03E-03	1.75E-49	1.66E-28	1.51E-01	0.59	61.36	1.88
BA32	0.63	1.85E-03	-1.54E-05	5.96E-04	1.53E-56	4.15E-34	4.67E-01	0.62	59.99	1.53
BA24	0.62	1.57E-03	-1.30E-05	6.07E-04	4.09E-49	3.12E-29	4.25E-01	0.57	60.15	1.76
BA10	0.64	2.21E-03	-1.96E-05	1.37E-03	1.39E-56	8.90E-38	1.64E-01	0.56	56.39	1.20
BA25	0.61	1.40E-03	-1.16E-05	1.64E-03	4.50E-38	3.63E-22	3.96E-02	0.50	60.57	1.95
BA11	0.63	2.36E-03	-2.10E-05	2.20E-03	5.94E-71	3.02E-48	1.48E-02	0.63	56.31	1.05
BA46	0.64	2.47E-03	-2.11E-05	1.16E-03	1.73E-76	3.44E-49	1.95E-01	0.69	58.59	1.18
BA17	0.69	1.55E-03	-1.41E-05	-1.86E-03	2.14E-24	1.58E-16	1.07E-01	0.29	55.18	2.00
BA18	0.68	2.05E-03	-1.89E-05	-5.36E-04	6.80E-49	1.78E-34	5.90E-01	0.47	54.28	1.21
BA27	0.65	1.50E-03	-1.16E-05	2.01E-03	3.25E-26	1.47E-13	5.94E-02	0.45	64.87	3.33
BA36	0.64	1.69E-03	-1.58E-05	2.84E-03	1.99E-44	5.94E-32	1.16E-03	0.44	53.53	1.18
BA35	0.64	1.04E-03	-7.27E-06	6.14E-04	2.46E-15	9.11E-07	5.46E-01	0.36	73.52	8.89
BA28	0.62	2.62E-03	-2.47E-05	4.80E-03	1.22E-47	9.25E-35	2.35E-04	0.46	53.15	1.02
BA29	0.64	1.39E-03	-1.12E-05	-2.61E-04	9.67E-22	6.91E-12	8.14E-01	0.36	62.87	3.53
BA26	0.64	1.68E-03	-1.42E-05	-1.55E-04	1.32E-14	8.13E-09	9.27E-01	0.23	59.71	3.74
BA33	0.61	1.44E-03	-1.21E-05	6.70E-04	1.22E-39	1.27E-23	4.01E-01	0.50	59.78	2.05
sWM										
BA6	1.06	1.90E-03	-3.08E-05	2.02E-03	6.86E-08	5.67E-14	4.65E-01	0.24	30.76	2.15
BA4	1.08	1.04E-03	-2.18E-05	1.31E-03	1.63E-03	1.24E-08	6.17E-01	0.27	23.26	4.40
BA9	1.06	1.41E-03	-2.59E-05	4.72E-03	1.70E-04	2.02E-09	1.10E-01	0.23	26.60	3.65
BA3	1.03	1.17E-03	-1.52E-05	9.34E-04	2.82E-04	3.75E-05	7.13E-01	0.03	37.68	8.07
BA1	1.02	1.49E-03	-1.95E-05	1.18E-03	3.46E-05	2.09E-06	6.78E-01	0.05	37.62	3.54
BA5	0.99	3.17E-03	-4.19E-05	4.58E-03	1.85E-15	8.70E-20	1.37E-01	0.18	37.73	1.18
BA7	1.04	1.55E-03	-2.44E-05	2.61E-03	6.92E-05	5.50E-08	3.96E-01	0.13	31.28	3.46
BA2	1.06	1.97E-03	-2.96E-05	-1.39E-03	1.57E-07	9.01E-12	6.37E-01	0.17	33.02	2.41
BA31	1.04	2.78E-03	-3.92E-05	1.79E-03	9.05E-13	3.04E-18	5.52E-01	0.21	35.28	1.46
BA40	1.01	2.73E-03	-3.59E-05	8.48E-03	5.56E-13	1.72E-16	3.97E-03	0.16	37.86	1.29
BA44	1.08	1.95E-03	-3.13E-05	-2.53E-04	2.84E-08	1.74E-14	9.27E-01	0.25	30.96	2.11
BA45	1.02	2.36E-03	-3.23E-05	2.44E-03	2.75E-10	7.28E-14	4.05E-01	0.14	36.46	1.56
BA23	1.03	2.85E-03	-3.98E-05	-1.27E-03	2.57E-12	3.20E-17	6.87E-01	0.19	35.72	1.57
BA39	1.06	1.91E-03	-2.95E-05	6.91E-03	3.85E-07	1.03E-11	1.95E-02	0.19	32.03	2.38
BA43	1.04	1.98E-03	-2.95E-05	2.76E-03	3.25E-06	1.99E-09	4.11E-01	0.13	33.36	2.63
BA19	1.06	2.15E-03	-3.16E-05	2.67E-03	1.27E-10	3.23E-16	3.07E-01	0.21	33.87	1.52
BA47	0.98	4.32E-03	-5.12E-05	2.93E-03	3.78E-29	7.52E-31	3.11E-01	0.22	42.24	0.73
BA41	1.07	2.19E-03	-3.31E-05	-4.31E-03	5.04E-09	2.39E-14	1.41E-01	0.21	32.93	1.96
BA30	1.03	2.75E-03	-3.82E-05	-6.04E-04	3.82E-10	4.60E-14	8.60E-01	0.15	35.76	1.87
BA22	1.04	2.89E-03	-3.53E-05	8.05E-05	4.40E-13	1.33E-14	9.79E-01	0.11	40.93	1.25
BA42	1.10	7.57E-04	-1.91E-05	-2.44E-03	3.64E-02	4.98E-06	3.95E-01	0.25	18.20	8.57
BA21	0.97	4.87E-03	-5.61E-05	6.63E-03	1.49E-30	6.85E-31	3.66E-02	0.23	43.41	0.77
BA38	0.88	4.81E-03	-4.94E-05	5.51E-03	6.77E-28	2.90E-23	9.48E-02	0.23	48.73	1.11
BA37	1.01	3.85E-03	-4.78E-05	6.41E-03	2.77E-22	1.46E-25	3.36E-02	0.20	40.28	0.85
BA20	0.91	5.70E-03	-6.21E-05	3.28E-03	1.61E-38	1.43E-35	3.07E-01	0.28	45.87	0.72
BA32	1.05	2.91E-03	-4.03E-05	2.21E-03	1.70E-14	4.64E-20	4.50E-01	0.21	36.03	1.23
BA24	1.03	2.99E-03	-3.99E-05	8.76E-03	7.35E-14	4.87E-18	4.78E-03	0.18	37.31	1.40
BA10	1.04	2.27E-03	-3.37E-05	4.24E-03	2.58E-09	2.18E-14	1.55E-01	0.20	33.51	2.06
BA25	0.92	3.37E-03	-4.32E-05	6.16E-03	3.22E-10	2.36E-12	1.41E-01	0.10	38.90	1.58
BA11	0.99	3.61E-03	-4.50E-05	7.07E-03	2.10E-19	1.85E-22	2.13E-02	0.18	40.06	1.04
BA46	1.07	1.44E-03	-2.49E-05	9.15E-04	1.10E-04	6.32E-09	7.55E-01	0.19	28.37	3.25
BA17	1.00	8.77E-04	-1.32E-05	2.19E-03	1.22E-02	9.71E-04	4.29E-01	0.04	32.37	4.13
BA18	1.02	1.74E-03	-2.36E-05	2.54E-03	3.33E-08	7.61E-11	3.03E-01	0.11	36.70	1.84
BA27	0.87	3.67E-03	-3.09E-05	5.37E-03	5.70E-12	3.09E-07	1.95E-01	0.19	60.10	4.35
BA36	0.86	4.35E-03	-4.25E-05	-3.91E-03	1.72E-31	3.90E-24	1.58E-01	0.29	51.27	1.24
BA35	0.93	4.87E-03	-5.19E-05	4.55E-03	3.05E-39	7.59E-35	9.46E-02	0.29	46.99	0.77
BA28	0.93	4.31E-03	-4.35E-05	-3.72E-03	7.13E-30	3.02E-24	1.89E-01	0.25	49.52	1.17
BA29	0.89	5.59E-03	-6.10E-05	-2.97E-04	2.12E-25	1.82E-23	9.41E-01	0.18	45.79	0.85
BA26	0.93	4.24E-03	-4.50E-05	-3.28E-03	4.22E-14	2.15E-12	4.51E-01	0.10	47.23	1.37
BA33	0.90	6.48E-03	-7.08E-05	4.91E-03	1.10E-40	8.83E-38	1.65E-01	0.29	45.73	0.70
WM Bundles										
ARCR	1.14	2.68E-03	-3.74E-05	2.49E-03	4.58E-12	6.95E-17	4.07E-01	0.19	35.75	1.45
ATRL	1.13	3.09E-03	-4.51E-05	3.19E-03	2.90E-13	4.27E-20	3.29E-01	0.26	34.14	1.41
ATRR	1.13	3.14E-03	-4.45E-05	3.58E-03	1.74E-13	2.07E-19	2.77E-01	0.23	35.22	1.31
CGCL	1.10	2.94E-03	-4.06E-05	6.10E-03	4.23E-13	5.04E-18	5.23E-02	0.20	36.16	1.25
CGCR	1.08	3.93E-03	-5.08E-05	7.73E-03	1.41E-22	8.72E-28	1.12E-02	0.25	38.64	0.89
CSTL	1.11	2.15E-03	-3.27E-05	2.26E-03	4.99E-10	3.18E-16	4.00E-01	0.24	32.70	1.78
CSTR	1.15	1.00E-03	-2.15E-05	2.06E-03	5.45E-03	2.57E-07	4.71E-01	0.24	22.28	5.91
FA	1.19	1.89E-03	-3.37E-05	1.86E-03	7.84E-06	6.96E-12	5.76E-01	0.27	27.80	2.81
FP	1.20	-3.34E-04	-1.31E-05	-4.51E-03	4.00E-01	3.87E-03	1.51E-01	0.41	-13.80	9.47
IFOL	1.16	1.39E-03	-3.05E-05	-2.31E-04	8.07E-04	2.28E-10	9.44E-01	0.34	22.16	4.22
IFOR	1.15	2.27E-03	-3.77E-05	-1.63E-03	2.27E-08	1.33E-15	6.08E-01	0.29	29.87	2.14

ILFL	1.12	2.86E-03	-4.41E-05	1.53E-03	3.55E-13	6.66E-22	6.16E-01	0.32	32.30	1.43	1.16	1.39E-03	-3.07E-05	-3.87E-04	4.33E-04	2.65E-11	8.03E-01	0.37	22.11	3.82	1.12	2.92E-03	-4.41E-05	1.60E-03	9.92E-14	4.69E-22	5.97E-01	0.31	32.96	1.36	1.13	2.36E-03	-3.76E-05	1.80E-03	1.07E-09	5.93E-17	5.51E-01	0.28	31.25	1.74
ILFR	1.08	4.96E-03	-6.23E-05	-3.64E-04	3.57E-33	1.15E-38	9.05E-01	0.30	39.83	0.64	1.16	1.47E-03	-3.04E-05	1.18E-03	1.62E-04	2.21E-11	4.42E-01	0.34	23.72	3.34	1.13	3.04E-03	-4.42E-05	-1.28E-03	3.64E-15	8.55E-23	6.67E-01	0.28	34.31	1.18	1.12	3.54E-03	-4.84E-05	-1.73E-03	3.39E-19	4.75E-26	5.64E-01	0.27	36.45	1.02
pARCL	1.10	3.33E-03	-4.31E-05	7.57E-03	7.77E-17	8.34E-21	1.36E-02	0.19	38.58	1.02	1.14	1.71E-03	-2.79E-05	-3.49E-03	1.69E-05	1.24E-09	2.59E-02	0.18	30.25	2.81	1.10	3.19E-03	-4.13E-05	7.53E-03	1.09E-15	2.24E-19	1.42E-02	0.18	38.59	1.09	1.09	3.42E-03	-4.32E-05	6.55E-03	8.86E-18	4.46E-21	3.18E-02	0.18	39.55	1.00
pARCR	1.06	5.43E-03	-6.37E-05	6.61E-03	6.53E-37	1.48E-38	3.52E-02	0.28	42.59	0.65	1.15	1.61E-03	-2.83E-05	-2.39E-03	4.51E-05	5.77E-10	1.22E-01	0.22	27.83	3.24	1.12	3.18E-03	-4.26E-05	5.51E-03	1.13E-15	1.63E-20	7.15E-02	0.20	37.28	1.12	1.09	4.09E-03	-5.10E-05	4.12E-03	3.24E-25	3.29E-29	1.65E-01	0.23	40.08	0.76
SLFL	1.14	2.18E-03	-3.33E-05	5.47E-03	7.11E-09	2.37E-14	6.33E-02	0.22	32.62	1.80	1.16	1.06E-03	-2.28E-05	-2.40E-03	5.38E-03	1.95E-07	1.08E-01	0.25	22.19	4.85	1.14	2.08E-03	-3.19E-05	5.46E-03	3.03E-08	2.12E-13	6.27E-02	0.21	32.46	1.89	1.12	2.60E-03	-3.62E-05	4.18E-03	6.30E-11	2.89E-15	1.76E-01	0.17	35.74	1.63
SLFR	1.11	3.94E-03	-5.06E-05	5.83E-03	6.08E-23	5.64E-28	5.38E-02	0.24	38.87	0.85	1.18	1.01E-03	-2.38E-05	-2.22E-03	8.18E-03	8.12E-08	1.41E-01	0.29	20.27	5.49	1.15	2.19E-03	-3.42E-05	5.06E-03	5.73E-09	4.05E-15	8.43E-02	0.24	31.85	1.83	1.14	2.86E-03	-4.01E-05	3.01E-03	5.08E-13	1.99E-18	3.24E-01	0.21	35.54	1.47
*R2**																																								
cGM																																								
BA6	-4.36	1.67E-01	-1.42E-03	1.08E-01	1.77E-24	4.01E-18	1.78E-01	0.38	58.89	1.51	-4.25	1.64E-01	-1.39E-03	-4.91E-02	8.82E-24	1.45E-17	2.21E-01	0.38	59.07	1.53	-4.20	1.61E-01	-1.37E-03	1.00E-01	2.41E-22	1.13E-16	2.20E-01	0.35	58.79	1.52	-4.35	1.65E-01	-1.39E-03	1.07E-01	2.11E-24	9.23E-18	1.76E-01	0.40	59.50	1.61
BA4	-4.16	1.61E-01	-1.37E-03	-4.24E-02	1.74E-22	1.43E-16	6.03E-01	0.36	59.06	1.74	-4.16	1.61E-01	-1.36E-03	2.42E-02	2.18E-22	1.27E-16	5.52E-01	0.35	58.98	1.71	-4.05	1.58E-01	-1.35E-03	-4.07E-02	3.62E-21	8.00E-16	6.23E-01	0.33	58.76	1.74	-4.12	1.59E-01	-1.33E-03	-3.61E-02	5.48E-22	5.81E-16	6.57E-01	0.37	59.61	1.83
BA9	-4.26	1.72E-01	-1.56E-03	1.30E-01	7.04E-23	8.99E-19	1.31E-01	0.29	55.29	1.15	-4.21	1.73E-01	-1.57E-03	-6.55E-02	4.34E-23	4.63E-19	1.28E-01	0.29	55.13	1.12	-4.17	1.69E-01	-1.53E-03	1.22E-01	3.92E-22	2.27E-18	1.59E-01	0.27	55.08	1.12	-4.45	1.79E-01	-1.61E-03	1.26E-01	2.17E-25	1.25E-20	1.32E-01	0.32	55.65	1.13
BA3	-2.29	9.58E-02	-9.00E-04	6.79E-02	4.88E-07	3.47E-06	4.89E-01	0.07	53.46	2.19	-2.33	9.98E-02	-9.47E-04	-3.71E-02	1.82E-07	1.15E-06	4.50E-01	0.07	52.89	1.95	-2.35	9.88E-02	-9.34E-04	7.47E-02	1.61E-07	1.05E-06	4.43E-01	0.07	53.14	2.00	-2.62	1.08E-01	-9.98E-04	7.10E-02	1.03E-08	1.92E-07	4.63E-01	0.10	54.28	1.98
BA1	-3.39	1.44E-01	-1.33E-03	-1.24E-01	3.95E-15	7.59E-13	1.80E-01	0.18	54.18	1.42	-3.47	1.45E-01	-1.35E-03	5.80E-02	1.85E-15	3.16E-13	2.08E-01	0.18	54.02	1.34	-3.40	1.45E-01	-1.34E-03	-1.20E-01	1.74E-15	2.84E-13	1.91E-01	0.18	54.04	1.33	-3.56	1.50E-01	-1.37E-03	-1.24E-01	1.44E-16	6.96E-14	1.72E-01	0.20	54.61	1.37
BA5	-3.24	1.27E-01	-1.13E-03	2.15E-01	2.99E-12	7.59E-10	2.06E-02	0.18	56.14	1.82	-3.12	1.26E-01	-1.13E-03	-1.07E-01	2.59E-12	6.07E-10	2.06E-02	0.17	56.21	1.76	-3.21	1.26E-01	-1.13E-03	2.13E-01	3.21E-12	6.73E-10	2.08E-02	0.17	56.06	1.74	-3.55	1.40E-01	-1.25E-03	1.91E-01	6.58E-15	6.01E-12	3.49E-02	0.21	56.14	1.61
BA7	-3.73	1.49E-01	-1.32E-03	1.00E-01	6.93E-17	1.77E-13	2.62E-01	0.23	56.27	1.73	-3.71	1.50E-01	-1.34E-03	-5.06E-02	2.13E-17	5.11E-14	2.55E-01	0.23	56.13	1.63	-3.71	1.48E-01	-1.33E-03	9.98E-02	6.25E-17	1.09E-13	2.62E-01	0.22	56.01	1.65	-3.89	1.55E-01	-1.38E-03	9.29E-02	1.46E-18	8.42E-15	2.91E-01	0.26	56.26	1.63
BA2	-3.85	1.58E-01	-1.44E-03	7.49E-03	1.07E-18	1.75E-15	9.33E-01	0.23	54.97	1.29	-3.82	1.57E-01	-1.43E-03	-5.83E-03	1.32E-18	1.77E-15	8.96E-01	0.23	54.90	1.24	-3.66	1.51E-01	-1.38E-03	1.31E-02	4.52E-17	2.56E-14	8.85E-01	0.20	54.76	1.30	-3.91	1.59E-01	-1.43E-03	1.96E-02	3.73E-19	1.27E-15	8.24E-01	0.25	55.47	1.31
BA31	-3.11	1.23E-01	-1.08E-03	1.04E-01	1.94E-11	4.91E-09	2.65E-01	0.17	56.83	1.94	-3.00	1.19E-01	-1.04E-03	-5.09E-02	5.10E-11	1.22E-08	2.73E-01	0.16	57.36	2.06	-2.96	1.16E-01	-1.02E-03	1.01E-01	1.79E-10	2.74E-08	2.80E-01	0.15	57.06	2.14	-3.11	1.22E-01	-1.07E-03	1.05E-01	2.37E-11	7.10E-09	2.59E-01	0.17	57.22	2.04
BA40	-3.99	1.56E-01	-1.37E-03	1.19E-01	1.84E-19	4.05E-15	1.67E-01	0.29	57.15	1.70	-3.96	1.58E-01	-1.38E-03	-6.19E-02	6.58E-20	1.31E-15	1.50E-01	0.29	57.07	1.58	-3.85	1.51E-01	-1.33E-03	1.22E-01	3.71E-18	2.73E-14	1.60E-01	0.26	56.95	1.65	-3.76	1.46E-01	-1.27E-03	1.28E-01	8.22E-17	7.20E-13	1.45E-01	0.26	57.70	1.88
BA44	-3.90	1.50E-01	-1.27E-03	6.21E-02	1.37E-18	1.09E-13	4.63E-01	0.31	59.10	1.95	-3.87	1.50E-01	-1.27E-03	-3.65E-02	1.04E-18	7.09E-14	3.89E-01	0.31	58.91	1.91	-3.80	1.46E-01	-1.24E-03	6.57E-02	1.10E-17	3.45E-13	4.42E-01	0.28	58.84	1.93	-4.02	1.52E-01	-1.28E-03	8.97E-02	6.07E-20	1.90E-14	2.79E-01	0.34	59.70	2.02
BA45	-3.10	1.25E-01	-1.11E-03	8.13E-03	1.04E-11	2.51E-09	9.30E-01	0.16	56.47	2.05	-3.27	1.34E-01	-1.22E-03	-1.02E-02	2.16E-13	3.60E-11	8.27E-01	0.16	55.03	1.60	-3.44	1.39E-01	-1.25E-03	1.64E-02	9.23E-15	5.36E-12	8.56E-01	0.19	55.85	1.65	-3.60	1.42E-01	-1.24E-03	2.17E-02	1.10E-15	3.94E-12	8.07E-01	0.23	57.35	1.83
BA23	-2.78	1.09E-01	-9.51E-04	-2.86E-03	2.80E-09	3.60E-07	9.76E-01	0.14	57.97	2.64	-2.77	1.09E-01	-9.46E-04	7.37E-03	2.96E-09	3.51E-07	8.76E-01	0.14	58.00	2.55	-2.71	1.07E-01	-9.29E-04	-1.34E-02	5.23E-09	5.12E-07	8.86E-01	0.13	57.91	2.60	-2.80	1.10E-01	-9.51E-04	2.67E-02	2.49E-09	3.46E-07	7.77E-01	0.14	58.07	2.60
BA39	-3.81	1.51E-01	-1.33E-03	6.93E-02	1.03E-17	6.75E-14	4.31E-01	0.25	56.84	1.73	-3.76	1.50E-01	-1.32E-03	-3.30E-02	9.29E-18	5.52E-14	4.52E-01	0.25	56.91	1.64	-3.72	1.47E-01	-1.30E-03	6.33E-02	5.36E-17	1.80E-13	4.73E-01	0.24	56.70	1.66	-3.76	1.47E-01	-1.28E-03	7.80E-02	4.64E-17	4.29E-13	3.74E-01	0.26	57.61	1.83
BA43	-3.61	1.40E-01	-1.21E-03	1.24E-01	2.06E-15	9.65E-12	1.62E-01	0.24	57.88	1.87	-3.57	1.41E-01	-1.23E-03	-6.59E-02	1.13E-15	4.43E-12	1.37E-01	0.24	57.61	1.81	-3.58	1.39E-01	-1.21E-03	1.34E-01	2.71E-15	7.58E-12	1.32E-01	0.23	57.53	1.81	-3.92	1.52E-01	-1.31E-03	1.05E-01	1.15E-18	3.61E-14	2.21E-01	0.29	57.99	1.71
BA19	-3.83	1.52E-01	-1.34E-03	2.99E-02	4.75E-18	4.08E-14	7.33E-01	0.26	56.93	1.64	-3.82	1.52E-01	-1.34E-03	-1.21E-02	3.59E-18	2.96E-14	7.83E-01	0.26	56.94	1.61	-3.75	1.49E-01	-1.31E-03	2.24E-02	1.59E-17	8.19E-14	7.98E-01	0.25	56.86	1.59	-3.73	1.47E-01	-1.29E-03	3.47E-02	6.04E-17	3.54E-13	6.94E-01	0.25	57.15	1.75
BA47	-1.60	5.65E-02	-4.43E-04	9.36E-02	2.78E-03	2.15E-02	3.42E-01	0.06	65.59	7.28	-1.63	6.22E-02	-5.23E-04	-3.58E-02	1.07E-03	6.60E-03	4.70E-01	0.05	60.36	4.30	-1.82	6.52E-02	-5.21E-04	1.12E-01	4.64E-04	5.77E-03	2.50E-01	0.08	63.58	4.99	-2.47	9.22E-02	-7.58E-04	3.44E-02	5.03E-07	4.73E-05	7.17E-01	0.14	61.57	4.69
BA41	-3.25	1.31E-01	-1.16E-03	-9.60E-02	3.82E-13	2.50E-10	2.95E-01	0.19	56.94	2.07	-3.22	1.28E-01	-1.12E-03	4.16E-02	1.21E-12	6.32E-10	3.64E-01	0.18	57.19	2.14	-3.04	1.23E-01	-1.08E-03	-7.71E-02	1.28E-11	3.34E-09	4.04E-01	0.16	56.95	2.22	-3.18	1.27E-01	-1.11E-03	-9.06E-02	1.81E-12	1.19E-09	3.23E-01	0.19	57.51	2.23
BA30	-2.66	1.06E-01	-9.36E-04	4.32E-02	1.32E-08	7.35E-07	6.52E-01	0.12	56.88	2.56	-2.70	1.09E-01	-9.77E-04	-1.86E-02	4.50E-09	2.22E-07	6.98E-01	0.12	56.19	2.36	-2.68	1.08E-01	-9.64E-04	3.33E-02	5.85E-09	2.62E-07	7.27E-01	0.11	56.15	2.31	-2.71	1.06E-01	-9.35E-04	7.67E-02	9.36E-09	6.76E-07	4.20E-01	0.13	57.30	2.67
BA22	-3.95	1.53E-01	-1.31E-03	5.71E-02	3.62E-19	2.26E-14	5.02E-01	0.30	58.40	1.80	-3.97	1.55E-01	-1.34E-03	-3.33E-02	4.83E-20	3.24E-15	4.30E-01	0.30	58.19	1.68	-3.98	1.54E-01	-1.33E-03	5.87E-02	1.05E-19	6.11E-15	4.87E-01	0.30	58.25	1.71	-4.02	1.53E-01	-1.29E-03	8.80E-02	6.20E-20	1.73E-14	2.89E-01	0.34	59.57	1.98
BA42	-3.68	1.43E-01	-1.25E-03	1.59E-01	5.11E-16	2.00E-12	7.41E-02	0.24	57.28	1.83	-3.64	1.45E-01	-1.28E-03	-8.79E-02	1.59E-16	5.34E-13	4.75E-02	0.24	56.90	1.71	-3.70	1.44E-01	-1.27E-03	1.71E-01	1.99E-16	5.75E-13	5.29E-02	0.24	56.87	1.72	-3.80	1.47E-01	-1.28E-03	1.57E-01	3.78E-17	3.89E-13	7.27E-02	0.27	57.70	1.80
BA21	-2.22	8.02E-02	-6.49E-04	1.45E-01	1.47E-05	5.54E-04	1.30E-01	0.11	62.32	3.85	-2.27	8.82E-02	-7.54E-04	-6.62E-02	1.84E-06	5.64E-05	1.67E-01	0.10	58.83	2.62	-2.63	9.69E-02	-8.04E-04	1.48E-01	8.30E-08	1.09E-05	1.13E-01	0.15	60.94	4.45	-2.92	1.05E-01	-8.36E-04	1.80E-01	3.11E-09	2.81E-06	4.76E-02	0.21	63.24	4.24
BA38	-1.69	5.66E-02	-4.30E-04	2.04E-01	2.49E-03	2.39E-02	3.72E-02	0.08	67.82	8.58	-1.75	6.70E-02	-5.63E-04	-7.26E-02	3.61E-04	3.14E-03	1.39E-01	0.06	60.47	4.40	-2.04	7.08E-02	-5.56E-04	2.01E-01	1.13E-04	2.74E-03	3.61E-02	0.11	64.93	5.54	-2.37	8.41E-02	-6.70E-04	1.78E-01	4.10E-06	3.04E-04	6.05E-02	0.14	64.13	8.28
BA37	-3.64	1.42E-01	-1.24E-03	8.57E-02	7.64E-16	3.38E-12	3.34E-01	0.24	57.46	1.59	-3.74	1.49E-01	-1.31E-03	-4.69E-02	2.39E-17	1.28E-13	2.87E-01	0.25	56.94	1.42	-3.77	1.48E-01	-1.29E-03	7.82E-02	1.96E-17	1.57E-13	3.70E-01	0.26	57.35	1.44	-3.68	1.42E-01	-1.23E-03	1.01E-01	4.70E-16	3.73E-12	2.51E-01	0.26	58.08	1.66
BA20	-2.28	7.89E-02	-5.84E-04	3.36E-02	1.18E-05	1.38E-03	7.18E-01	0.16	68.73	5.86	-2.52	9.10E-02	-7.10E-04	-2.87E-02	3.41E-07	8.21E-05	5.34E-01	0.17	65.21	7.25	-2.49	8.75E-02	-6.66E-04	4.83E-02	7.75E-07	1.96E-04	5.98E-01	0.18	66.46	4.33	-2.85	1.02E-01	-7.89E-04	6.45E-02	6.73E-09	8.41E-06	4.73E-01	0.22	65.18	4.86
BA32	-3.94	1.57E-01	-1.42E-03	1.95E-01	7.04E-19	2.22E-15	2.69E-02	0.25	55.53	1.38	-3.84	1.56E-01	-1.41E-03	-9.10E-02	7.79E-19	2.22E-15	3.93E-02	0.25	55.59	1.32	-3.82	1.52E-01	-1.38E-03	1.81E-01	6.31E-18	1.06E-14	4.14E-02	0.23	55.49	1.35	-3.89	1.54E-01	-1.38E-03	2.06E-01	2.88E-18	1.02E-14	1.95E-02	0.25	55.93	1.44
BA24	-2.98	1.12E-01	-9.49E-04	1.98E-01	6.12E-10	2.12E-07	3.25E-02	0.18	59.26	2.67	-2.80	1.07E-01	-8.95E-04	-9.32E-02	2.41E-09	7.71E-07	4.43E-02	0.18	60.26	2.90	-2.76	1.03E-01	-8.64E-04	1.69E-01	1.22E-08	2.12E-06	6.92E-02	0.16	59.99	3.01	-2.87	1.07E-01	-9.12E-04	2.01E-01	3.50E-09	7.26E-07	3.11E-02	0.16	59.20	2.75
BA10	-3.37	1.34E-01	-1.22E-03	2.71E-01	1.88E-13	3.49E-11	3.40E-03	0.18	54.93	1.60	-3.29	1.37E-01	-1.26E-03	-1.31E-01	3.60E-14	5.47E-12	4.38E-03	0.18	54.53	1.47	-3.41	1.37E-01	-1.26E-03	2.60E-01	3.88E-14	5.59E-12	4.67E-03	0.18	54.45	1.47	-3.72	1.48E-01	-1.34E-03	2.51E-01	1.54E-16	1.29E-13	5.42E-03	0.22	55.20	1.47
BA25	-1.24	4.91E-02	-4.48E-04	1.15E-01	1.10E-02	2.29E-02	2.53E-01	0.02	55.27	3.53	-1.26	5.38E-02	-5.06E-04	-5.92E-02	4.92E-03	9.27E-03	2.37E-01	0.02	53.48	2.66	-1.40	5.65E-02	-5.27E-04	1.30E-01	3.08E-03	6.63E-03	1.93E-01	0.02	53.90	2.59	-1.35	5.26E-02	-4.74E-04	1.19E-01	6.36E-03	1.58E-02	2.38E-01	0.02	56.01	3.43
BA11	-1.99	7.47E-02	-6.34E-04	1.14E-01	7.59E-05	9.60E-04	2.46E-01	0.07	59.49	3.44	-2.12	8.39E-02	-7.32E-04	-7.20E-02	7.00E-06	1.10E-04	1.38E-01	0.08	58.02	4.52	-2.17	8.30E-02	-7.18E-04	1.24E-01	8.55E-06	1.45E-04	2.00E-01	0.08	58.62	5.04	-2.66	1.00E-01	-8.55E-04	1.45E-01	4.60E-08	4.54E-06	1.27E-01	0.14	59.27	3.31
BA46	-3.26	1.26E-01	-1.10E-03	1.26E-01	2.44E-12	1.64E-09	1.68E-01	0.19	57.64	2.31	-3.25	1.29E-01	-1.13E-03	-6.29E-02	6.37E-13	3.79E-10	1.68E-01	0.19	57.18	2.08	-3.26	1.27E-01	-1.12E-03	1.24E-01	1.30E-12	6.45E-10	1.74E-01	0.18	57.16	2.11	-3.68	1.42E-01	-1.23E-03	1.16E-01	4.77E-16	3.29E-12	1.88E-01	0.25	58.00	2.00
BA17	-2.90	1.19E-01	-1.09E-03	3.89E-02	1.68E-10	9.12E-09	6.83E-01	0.13	54.92	1.98	-2.90	1.20E-01	-1.09E-03	-2.00E-02	1.03E-10	5.34E-09	6.74E-01	0.13	54.93	1.91	-2.79	1.14E-01	-1.05E-03	4.53E-02	6.87E-10	2.27E-08	6.34E-01	0.11	54.69	1.94	-2.57	1.05E-01	-9.59E-04	4.89E-02	2.36E-08	5.34E-07	6.13E-01	0.10	55.06	2.35
BA18	-3.66	1.46E-01	-1.31E-03	1.02E-01	3.36E-16	5.19E-13	2.55E-01	0.22	56.05	1.57	-3.65	1.48E-01	-1.33E-03	-5.31E-02	1.11E-16	1.51E-13	2.37E-01	0.22	55.77	1.49	-3.60	1.44E-01	-1.30E-03	1.04E-01	6.24E-16	5.22E-13	2.49E-01	0.21	55.68	1.52	-3.63	1.44E-01	-1.28E-03	1.06E-01	7.43E-16	1.31E-12	2.40E-01	0.22	56.40	1.65
BA27	-2.26	9.11E-02	-8.07E-04	-2.80E-02	1.37E-06	2.60E-05	7.74E-01	0.08	56.97	3.32	-2.28	9.04E-02	-7.95E-04	1.28E-02	1.28E-06	2.67E-05	7.93E-01	0.09	57.52	3.78	-2.19	8.77E-02	-7.71E-04	-3.87E-02	2.74E-06	4.69E-05	6.90E-01	0.08	57.41	3.56	-2.47	9.93E-02	-8.80E-04	-1.27E-02	1.16E-07	3.80E-06	8.95E-01	0.10	56.88	2.88
BA36	-2.33	9.15E-02	-7.98E-04	2.78E-02	1.07E-06	2.85E-05	7.74E-01	0.09	57.83	3.07	-2.39	9.73E-02	-8.79E-04	-1.26E-02	1.92E-07	3.47E-06	7.94E-01	0.09	55.67	2.34	-2.70	1.07E-01	-9.39E-04	1.04E-02	5.83E-09	4.36E-07	9.13E-01	0.13	57.23	2.27	-2.57	1.00E-01	-8.69E-04	3.45E-02	6.69E-08	4.09E-06	7.18E-01	0.12	58.06	2.77
BA35	-2.13	8.01E-02	-6.53E-04	-2.50E-02	1.66E-05	5.52E-04	7.95E-01	0.10	62.16	4.12	-2.07	7.59E-02	-6.05E-04	1.25E-02	4.05E-05	1.27E-03	7.94E-01	0.11	63.72	4.80	-1.98	7.36E-02	-5.90E-04	-2.91E-02	6.82E-05	1.63E-03	7.62E-01	0.09	63.43	4.81	-2.20	8.29E-02	-6.78E-04	-2.56E-02	7.96E-06	3.22E-04	7.90E-01	0.11	61.82	3.83
BA28	-1.89	7.23E-02	-6.43E-04	1.90E-01	1.41E-04	9.02E-04	5.48E-02	0.06	57.22	8.17	-1.96	8.03E-02	-7.24E-04	-9.14E-02	2.06E-05	1.52E-04	6.30E-02	0.07	56.10	5.42	-2.04	7.76E-02	-6.85E-04	2.01E-01	3.42E-05	3.09E-04	3.96E-02	0.07	56.98	2.64	-2.29	8.76E-02	-7.73E-04	1.99E-01	3.09E-06	5.21E-05	4.12E-02	0.09	57.37	3.92
BA29	-1.48	5.05E-02	-3.82E-04	1.16E-01	7.53E-03	4.72E-02	2.40E-01	0.06	67.41	7.85	-1.49	5.44E-02	-4.32E-04	-5.54E-02	3.82E-03	2.38E-02	2.61E-01	0.05	65.02	8.19	-1.53	5.41E-02	-4.29E-04	1.08E-01	3.96E-03	2.45E-02	2.70E-01	0.05	65.01	7.98	-1.35	4.44E-02	-3.20E-04	1.18E-01	1.90E-02	9.69E-02	2.32E-01	0.06	69.98	8.66
BA26	-0.94	2.34E-02	-8.85E-05	1.07E-01	2.13E-01	6.45E-01	2.77E-01	0.06	73.26	9.51	-0.99	2.98E-02	-1.67E-04	-5.49E-02	1.13E-01	3.83E-01	2.67E-01	0.05	73.70	9.12	-1.07	3.03E-02	-1.67E-04	1.08E-01	1.05E-01	3.80E-01	2.71E-01	0.06	74.62	9.30	-1.00	2.46E-02	-8.94E-05	1.24E-01	1.88E-01	6.40E-01	2.05E-01	0.07	76.85	9.35
BA33	-1.69	5.82E-02	-4.77E-04	2.90E-01	1.98E-03	1.28E-02	3.30E-03	0.07	62.31	5.38	-1.56	5.90E-02	-4.83E-04	-1.52E-01	1.52E-03	1.05E-02	1.99E-03	0.08	62.22	5.15	-1.71	5.84E-02	-4.74E-04	2.93E-01	1.67E-03	1.20E-02	2.74E-03	0.08	62.87	5.46	-1.80	6.26E-02	-5.18E-04	3.00E-01	8.47E-04	6.72E-03	2.27E-03	0.08	61.42	4.54
sWM																																								
BA6	-3.96	1.68E-01	-1.62E-03	1.70E-01	2.20E-20	2.04E-18	5.99E-02	0.22	52.13	1.02	-3.89	1.70E-01	-1.63E-03	-8.24E-02	5.93E-21	3.74E-19	6.70E-02	0.22	51.96	0.97	-3.96	1.69E-01	-1.63E-03	1.61E-01	7.61E-21	4.32E-19	7.39E-02	0.21	51.88	0.97	-4.38	1.85E-01	-1.76E-03	1.48E-01	3.55E-25	2.06E-22	8.99E-02	0.27	52.64	0.95
BA4	-4.12	1.69E-01	-1.58E-03	2.26E-01	2.75E-21	2.23E-18	1.06E-02	0.25	53.60	1.05	-3.98	1.68E-01	-1.57E-03	-1.09E-01	3.29E-21	2.10E-18	1.33E-02	0.24	53.57	1.02	-4.04	1.67E-01	-1.56E-03	2.17E-01	8.19E-21	3.76E-18	1.44E-02	0.24	53.44	1.01	-4.39	1.79E-01	-1.66E-03	2.11E-01	1.54E-24	6.21E-21	1.44E-02	0.29	54.00	1.01
BA9	-3.74	1.71E-01	-1.75E-03	1.51E-01	1.14E-20	9.24E-21	9.75E-02	0.20	48.87	0.76	-3.68	1.73E-01	-1.78E-03	-6.84E-02	2.00E-21	9.23E-22	1.29E-01	0.21	48.65	0.72	-3.63	1.68E-01	-1.73E-03	1.32E-01	3.05E-20	1.20E-20	1.45E-01	0.20	48.59	0.75	-3.91	1.78E-01	-1.80E-03	1.27E-01	3.16E-22	5.32E-22	1.59E-01	0.22	49.30	0.74
BA3	-3.53	1.42E-01	-1.29E-03	1.60E-01	5.17E-15	2.44E-12	8.09E-02	0.20	55.29	1.59	-3.47	1.43E-01	-1.30E-03	-7.82E-02	2.73E-15	1.05E-12	8.71E-02	0.19	54.98	1.48	-3.52	1.42E-01	-1.30E-03	1.53E-01	3.33E-15	1.06E-12	9.31E-02	0.19	54.90	1.47	-3.88	1.56E-01	-1.42E-03	1.44E-01	1.97E-18	3.64E-15	1.07E-01	0.24	55.19	1.39
BA1	-3.74	1.54E-01	-1.42E-03	4.73E-02	1.49E-17	9.96E-15	6.01E-01	0.21	54.53	1.22	-3.74	1.56E-01	-1.43E-03	-2.29E-02	5.86E-18	3.33E-15	6.12E-01	0.21	54.34	1.18	-3.77	1.56E-01	-1.44E-03	4.09E-02	3.59E-18	1.99E-15	6.49E-01	0.21	54.33	1.15	-3.93	1.62E-01	-1.48E-03	3.17E-02	1.63E-19	2.77E-16	7.21E-01	0.24	54.72	1.16
BA5	-3.17	1.34E-01	-1.30E-03	3.07E-01	6.26E-13	5.90E-12	1.20E-03	0.15	51.44	1.21	-3.01	1.33E-01	-1.30E-03	-1.48E-01	4.53E-13	3.54E-12	1.74E-03	0.14	51.38	1.18	-3.15	1.33E-01	-1.30E-03	2.92E-01	5.05E-13	3.72E-12	1.89E-03	0.14	51.33	1.19	-3.45	1.45E-01	-1.40E-03	2.82E-01	3.69E-15	7.27E-14	2.49E-03	0.17	51.75	1.15
BA7	-3.69	1.54E-01	-1.48E-03	2.83E-01	3.09E-17	1.51E-15	2.06E-03	0.19	52.17	1.12	-3.58	1.56E-01	-1.50E-03	-1.38E-01	9.65E-18	3.82E-16	2.51E-03	0.19	52.02	1.07	-3.65	1.53E-01	-1.48E-03	2.68E-01	2.90E-17	9.20E-16	3.41E-03	0.19	51.97	1.07	-3.76	1.56E-01	-1.49E-03	2.66E-01	8.81E-18	6.95E-16	3.64E-03	0.20	52.49	1.14
BA2	-3.53	1.47E-01	-1.39E-03	1.56E-01	1.25E-15	1.08E-13	9.19E-02	0.18	53.14	1.29	-3.44	1.47E-01	-1.39E-03	-7.01E-02	8.99E-16	6.22E-14	1.29E-01	0.17	53.08	1.26	-3.37	1.42E-01	-1.34E-03	1.38E-01	1.26E-14	5.08E-13	1.39E-01	0.16	52.84	1.27	-3.77	1.57E-01	-1.47E-03	1.50E-01	6.99E-18	1.43E-15	9.90E-02	0.20	53.33	1.20
BA31	-2.99	1.26E-01	-1.23E-03	2.71E-01	1.39E-11	9.83E-11	4.55E-03	0.13	51.37	1.28	-2.83	1.25E-01	-1.22E-03	-1.28E-01	1.40E-11	8.47E-11	7.16E-03	0.12	51.37	1.23	-2.92	1.24E-01	-1.21E-03	2.50E-01	2.64E-11	1.41E-10	8.55E-03	0.12	51.28	1.24	-3.12	1.32E-01	-1.28E-03	2.59E-01	1.40E-12	1.34E-11	6.48E-03	0.14	51.53	1.23
BA40	-3.73	1.58E-01	-1.54E-03	2.90E-01	4.84E-18	1.17E-16	1.58E-03	0.20	51.43	1.02	-3.62	1.61E-01	-1.57E-03	-1.42E-01	9.60E-19	1.71E-17	1.86E-03	0.20	51.27	0.97	-3.63	1.55E-01	-1.52E-03	2.68E-01	1.49E-17	1.89E-16	3.52E-03	0.18	51.17	0.99	-3.75	1.58E-01	-1.53E-03	2.68E-01	5.14E-18	1.93E-16	3.43E-03	0.20	51.80	1.04
BA44	-3.05	1.32E-01	-1.29E-03	1.60E-01	2.20E-12	1.55E-11	9.33E-02	0.12	51.20	1.36	-3.00	1.34E-01	-1.32E-03	-8.10E-02	5.69E-13	2.76E-12	8.73E-02	0.13	50.88	1.27	-3.01	1.31E-01	-1.29E-03	1.58E-01	1.85E-12	7.82E-12	9.62E-02	0.12	50.80	1.31	-3.24	1.39E-01	-1.35E-03	1.52E-01	9.44E-14	1.27E-12	1.07E-01	0.14	51.69	1.33
BA45	-2.87	1.29E-01	-1.30E-03	9.78E-02	8.63E-12	1.77E-11	3.09E-01	0.11	49.81	1.20	-2.88	1.33E-01	-1.34E-03	-4.70E-02	1.46E-12	1.90E-12	3.26E-01	0.12	49.51	1.11	-3.01	1.36E-01	-1.37E-03	9.40E-02	2.78E-13	4.39E-13	3.20E-01	0.12	49.65	1.10	-2.88	1.27E-01	-1.26E-03	1.01E-01	1.58E-11	5.69E-11	2.96E-01	0.11	50.55	1.28
BA23	-2.75	1.25E-01	-1.26E-03	6.49E-02	4.29E-11	6.72E-11	5.02E-01	0.10	49.52	1.11	-2.70	1.25E-01	-1.26E-03	-2.57E-02	3.54E-11	4.20E-11	5.94E-01	0.10	49.37	1.07	-2.63	1.21E-01	-1.22E-03	5.05E-02	1.35E-10	1.43E-10	6.00E-01	0.09	49.26	1.09	-2.65	1.20E-01	-1.21E-03	6.56E-02	2.75E-10	4.56E-10	4.99E-01	0.09	49.68	1.18
BA39	-3.55	1.51E-01	-1.48E-03	3.00E-01	1.97E-16	2.58E-15	1.25E-03	0.18	51.17	1.08	-3.41	1.52E-01	-1.49E-03	-1.37E-01	8.73E-17	8.19E-16	3.07E-03	0.18	51.00	1.03	-3.46	1.49E-01	-1.46E-03	2.66E-01	4.88E-16	3.57E-15	4.10E-03	0.17	50.90	1.05	-3.81	1.60E-01	-1.54E-03	2.86E-01	1.38E-18	6.77E-17	1.75E-03	0.21	51.95	1.09
BA43	-3.37	1.47E-01	-1.44E-03	1.51E-01	3.56E-15	3.50E-14	1.07E-01	0.15	51.07	1.12	-3.32	1.48E-01	-1.46E-03	-8.06E-02	9.89E-16	7.45E-15	8.39E-02	0.16	50.93	1.05	-3.36	1.46E-01	-1.44E-03	1.53E-01	2.15E-15	1.44E-14	1.01E-01	0.15	50.86	1.06	-3.54	1.54E-01	-1.50E-03	1.38E-01	1.17E-16	1.72E-15	1.39E-01	0.17	51.25	1.08
BA19	-3.61	1.49E-01	-1.41E-03	2.65E-01	3.99E-16	3.33E-14	4.09E-03	0.19	52.96	1.24	-3.48	1.49E-01	-1.41E-03	-1.25E-01	2.50E-16	1.75E-14	6.42E-03	0.19	52.85	1.22	-3.53	1.46E-01	-1.39E-03	2.43E-01	8.44E-16	4.41E-14	8.29E-03	0.18	52.76	1.21	-3.66	1.51E-01	-1.42E-03	2.50E-01	1.29E-16	1.60E-14	6.54E-03	0.20	53.21	1.25
BA47	-1.73	8.01E-02	-8.39E-04	1.32E-01	3.02E-05	1.88E-05	1.85E-01	0.04	47.66	1.94	-1.67	8.04E-02	-8.41E-04	-5.02E-02	2.57E-05	1.52E-05	3.13E-01	0.04	47.79	1.92	-1.73	8.05E-02	-8.48E-04	1.41E-01	2.22E-05	1.15E-05	1.53E-01	0.05	47.45	1.89	-2.42	1.09E-01	-1.10E-03	9.01E-02	1.14E-08	1.74E-08	3.58E-01	0.08	49.70	1.40
BA41	-2.62	1.22E-01	-1.23E-03	-7.19E-02	1.30E-10	1.93E-10	4.58E-01	0.10	49.52	1.21	-2.62	1.21E-01	-1.22E-03	2.76E-02	1.37E-10	1.68E-10	5.67E-01	0.09	49.44	1.20	-2.52	1.18E-01	-1.19E-03	-5.71E-02	4.10E-10	4.58E-10	5.54E-01	0.09	49.37	1.21	-2.63	1.20E-01	-1.21E-03	-5.77E-02	2.18E-10	4.62E-10	5.52E-01	0.09	50.00	1.28
BA30	-2.44	1.09E-01	-1.10E-03	1.72E-01	9.99E-09	1.28E-08	7.82E-02	0.08	49.45	1.43	-2.40	1.12E-01	-1.15E-03	-8.04E-02	2.72E-09	2.28E-09	9.81E-02	0.09	48.99	1.30	-2.43	1.10E-01	-1.13E-03	1.53E-01	4.62E-09	3.74E-09	1.14E-01	0.08	48.96	1.33	-2.64	1.17E-01	-1.17E-03	1.70E-01	7.46E-10	1.50E-09	8.08E-02	0.09	50.02	1.39
BA22	-3.88	1.68E-01	-1.63E-03	1.43E-01	5.53E-20	2.14E-18	1.17E-01	0.20	51.52	0.93	-3.83	1.69E-01	-1.64E-03	-6.79E-02	1.46E-20	4.07E-19	1.33E-01	0.20	51.43	0.89	-3.86	1.68E-01	-1.63E-03	1.33E-01	3.08E-20	7.33E-19	1.43E-01	0.20	51.34	0.89	-4.02	1.72E-01	-1.65E-03	1.41E-01	3.89E-21	4.28E-19	1.16E-01	0.22	52.12	0.96
BA42	-3.53	1.43E-01	-1.33E-03	2.53E-01	4.29E-15	6.13E-13	6.03E-03	0.19	53.86	1.42	-3.41	1.44E-01	-1.35E-03	-1.29E-01	1.64E-15	1.68E-13	4.96E-03	0.18	53.57	1.32	-3.47	1.42E-01	-1.33E-03	2.51E-01	5.22E-15	4.00E-13	6.35E-03	0.18	53.43	1.32	-3.72	1.51E-01	-1.40E-03	1.91E-01	6.66E-17	2.57E-14	3.55E-02	0.21	54.24	1.36
BA21	-2.84	1.24E-01	-1.23E-03	1.91E-01	5.57E-11	1.78E-10	4.77E-02	0.11	50.46	1.28	-2.81	1.28E-01	-1.28E-03	-8.11E-02	7.42E-12	1.64E-11	8.92E-02	0.11	50.06	1.16	-3.12	1.36E-01	-1.35E-03	1.85E-01	2.13E-13	7.94E-13	5.01E-02	0.13	50.52	1.11	-3.33	1.42E-01	-1.38E-03	1.83E-01	1.86E-14	2.80E-13	5.22E-02	0.15	51.62	1.17
BA38	-2.38	1.01E-01	-9.78E-04	1.06E-01	1.00E-07	4.71E-07	2.82E-01	0.07	52.06	1.81	-2.51	1.11E-01	-1.09E-03	-5.14E-02	3.84E-09	1.43E-08	2.90E-01	0.08	51.28	1.51	-2.78	1.19E-01	-1.15E-03	8.57E-02	1.97E-10	1.57E-09	3.71E-01	0.10	52.07	1.47	-3.08	1.29E-01	-1.22E-03	1.07E-01	5.08E-12	1.43E-10	2.61E-01	0.13	53.05	1.49
BA37	-3.92	1.70E-01	-1.67E-03	1.78E-01	1.48E-20	3.44E-19	4.97E-02	0.21	51.09	0.85	-3.85	1.72E-01	-1.69E-03	-8.25E-02	4.05E-21	6.21E-20	6.82E-02	0.21	50.87	0.80	-4.02	1.75E-01	-1.71E-03	1.61E-01	3.93E-22	8.65E-21	7.27E-02	0.22	51.08	0.80	-3.78	1.63E-01	-1.59E-03	1.89E-01	7.30E-19	1.71E-17	3.94E-02	0.19	51.30	0.90
BA20	-3.14	1.32E-01	-1.23E-03	8.94E-04	1.36E-12	7.72E-11	9.92E-01	0.14	53.81	1.65	-3.17	1.34E-01	-1.26E-03	-6.90E-03	3.21E-13	1.51E-11	8.83E-01	0.14	53.48	1.55	-3.21	1.35E-01	-1.26E-03	2.40E-03	1.95E-13	1.12E-11	9.80E-01	0.15	53.66	1.51	-3.31	1.38E-01	-1.27E-03	4.41E-02	9.38E-14	1.01E-11	6.37E-01	0.16	54.12	1.62
BA32	-3.58	1.67E-01	-1.74E-03	2.00E-01	7.69E-20	1.30E-20	2.78E-02	0.21	47.84	0.74	-3.47	1.67E-01	-1.75E-03	-9.38E-02	3.47E-20	3.98E-21	3.78E-02	0.21	47.78	0.72	-3.44	1.62E-01	-1.69E-03	1.80E-01	5.15E-19	5.34E-20	4.77E-02	0.20	47.68	0.74	-3.67	1.69E-01	-1.74E-03	2.04E-01	3.28E-20	1.25E-20	2.52E-02	0.21	48.37	0.73
BA24	-2.83	1.16E-01	-1.14E-03	4.23E-01	3.47E-10	1.77E-09	1.00E-05	0.14	51.28	1.37	-2.62	1.17E-01	-1.14E-03	-1.99E-01	2.94E-10	1.25E-09	3.48E-05	0.13	51.21	1.30	-2.70	1.12E-01	-1.10E-03	3.81E-01	1.29E-09	4.61E-09	6.85E-05	0.12	51.09	1.35	-2.83	1.17E-01	-1.14E-03	4.11E-01	2.99E-10	1.52E-09	1.85E-05	0.13	51.25	1.36
BA10	-3.18	1.47E-01	-1.54E-03	2.73E-01	1.73E-15	3.55E-16	3.37E-03	0.17	47.72	0.88	-3.09	1.50E-01	-1.58E-03	-1.30E-01	2.37E-16	2.82E-17	4.72E-03	0.18	47.52	0.83	-3.12	1.46E-01	-1.53E-03	2.54E-01	1.41E-15	1.63E-16	6.04E-03	0.17	47.47	0.85	-3.21	1.48E-01	-1.54E-03	2.61E-01	1.24E-15	3.39E-16	5.15E-03	0.17	47.94	0.89
BA25	-1.75	8.32E-02	-8.98E-04	1.91E-01	1.28E-05	4.10E-06	5.43E-02	0.06	46.22	1.82	-1.73	8.73E-02	-9.45E-04	-8.42E-02	3.60E-06	8.43E-07	8.52E-02	0.07	46.10	1.68	-1.96	9.36E-02	-1.01E-03	1.94E-01	6.10E-07	1.26E-07	4.62E-02	0.08	46.24	1.53	-1.35	6.54E-02	-7.27E-04	2.13E-01	6.19E-04	1.95E-04	3.28E-02	0.05	44.66	2.82
BA11	-2.43	1.12E-01	-1.16E-03	1.49E-01	3.86E-09	2.47E-09	1.25E-01	0.09	48.36	1.36	-2.44	1.16E-01	-1.21E-03	-7.86E-02	5.84E-10	2.68E-10	1.04E-01	0.10	48.16	1.27	-2.51	1.16E-01	-1.21E-03	1.45E-01	5.45E-10	2.53E-10	1.32E-01	0.10	48.18	1.26	-2.86	1.28E-01	-1.30E-03	1.58E-01	1.03E-11	1.59E-11	1.01E-01	0.11	49.49	1.19
BA46	-2.80	1.26E-01	-1.29E-03	1.84E-01	2.27E-11	2.36E-11	5.59E-02	0.11	49.08	1.17	-2.74	1.28E-01	-1.32E-03	-9.06E-02	7.01E-12	4.77E-12	5.78E-02	0.12	48.81	1.08	-2.75	1.25E-01	-1.28E-03	1.72E-01	2.06E-11	1.35E-11	7.10E-02	0.11	48.74	1.13	-2.81	1.27E-01	-1.29E-03	1.47E-01	1.67E-11	1.90E-11	1.25E-01	0.11	49.20	1.18
BA17	-3.00	1.20E-01	-1.08E-03	1.24E-01	9.51E-11	9.20E-09	1.88E-01	0.14	55.77	2.00	-2.96	1.21E-01	-1.09E-03	-5.79E-02	4.73E-11	4.33E-09	2.19E-01	0.14	55.62	1.88	-2.91	1.17E-01	-1.06E-03	1.14E-01	2.10E-10	1.23E-08	2.27E-01	0.13	55.34	1.91	-2.82	1.12E-01	-1.01E-03	1.17E-01	1.51E-09	9.34E-08	2.18E-01	0.13	56.03	2.24
BA18	-3.81	1.52E-01	-1.38E-03	2.36E-01	1.63E-17	1.97E-14	8.55E-03	0.23	55.15	1.36	-3.74	1.54E-01	-1.41E-03	-1.18E-01	4.34E-18	4.56E-15	8.24E-03	0.23	54.89	1.26	-3.77	1.51E-01	-1.38E-03	2.26E-01	2.04E-17	1.36E-14	1.17E-02	0.22	54.78	1.28	-3.85	1.53E-01	-1.38E-03	2.26E-01	7.30E-18	1.43E-14	1.13E-02	0.24	55.48	1.37
BA27	-2.41	9.36E-02	-8.16E-04	8.85E-02	5.57E-07	1.78E-05	3.59E-01	0.10	57.92	3.44	-2.32	9.10E-02	-7.88E-04	-4.02E-02	9.51E-07	2.81E-05	4.04E-01	0.10	58.42	3.82	-2.39	9.21E-02	-7.95E-04	8.28E-02	6.50E-07	2.21E-05	3.87E-01	0.10	58.55	3.73	-2.64	1.03E-01	-9.06E-04	9.55E-02	2.89E-08	1.60E-06	3.18E-01	0.12	57.29	2.85
BA36	-2.14	8.84E-02	-8.01E-04	-4.48E-02	3.23E-06	3.39E-05	6.48E-01	0.07	55.63	3.44	-2.35	9.86E-02	-9.16E-04	2.19E-02	1.63E-07	1.62E-06	6.53E-01	0.08	54.08	2.26	-2.46	1.03E-01	-9.36E-04	-6.49E-02	4.01E-08	7.60E-07	5.00E-01	0.09	55.08	2.30	-2.65	1.08E-01	-9.73E-04	-2.99E-02	7.46E-09	3.05E-07	7.55E-01	0.11	55.86	2.37
BA35	-2.26	9.23E-02	-8.46E-04	6.78E-02	1.17E-06	1.19E-05	4.89E-01	0.07	54.86	2.87	-2.13	8.73E-02	-7.93E-04	-2.33E-02	4.17E-06	3.86E-05	6.36E-01	0.07	55.53	3.30	-2.11	8.59E-02	-7.83E-04	5.07E-02	5.29E-06	4.27E-05	6.04E-01	0.06	55.34	3.23	-2.55	1.04E-01	-9.50E-04	7.03E-02	3.55E-08	7.06E-07	4.68E-01	0.10	54.94	2.46
BA28	-2.77	1.13E-01	-1.03E-03	1.17E-01	1.83E-09	5.52E-08	2.22E-01	0.11	54.68	2.06	-2.73	1.14E-01	-1.06E-03	-6.00E-02	8.05E-10	2.05E-08	2.09E-01	0.11	54.28	1.90	-2.92	1.18E-01	-1.08E-03	1.14E-01	1.42E-10	6.40E-09	2.28E-01	0.13	54.76	1.85	-2.93	1.19E-01	-1.10E-03	1.21E-01	1.52E-10	6.74E-09	2.04E-01	0.13	54.57	1.92
BA29	-1.88	7.65E-02	-7.20E-04	1.78E-01	6.44E-05	2.22E-04	7.42E-02	0.05	53.32	1.98	-1.82	7.87E-02	-7.50E-04	-8.85E-02	3.83E-05	1.15E-04	7.42E-02	0.05	52.82	2.92	-1.92	7.89E-02	-7.50E-04	1.73E-01	3.18E-05	9.80E-05	7.93E-02	0.05	52.97	6.58	-1.89	7.68E-02	-7.20E-04	1.55E-01	5.97E-05	2.24E-04	1.20E-01	0.05	53.84	3.16
BA26	-1.72	6.75E-02	-6.17E-04	1.81E-01	4.17E-04	1.55E-03	7.04E-02	0.04	55.26	3.18	-1.65	6.93E-02	-6.42E-04	-8.90E-02	2.81E-04	9.21E-04	7.45E-02	0.04	55.06	9.08	-1.73	6.87E-02	-6.36E-04	1.74E-01	2.92E-04	9.64E-04	7.85E-02	0.04	54.50	2.85	-1.71	6.81E-02	-6.26E-04	1.55E-01	3.79E-04	1.34E-03	1.20E-01	0.04	54.89	3.08
BA33	-2.51	1.13E-01	-1.18E-03	3.76E-01	1.42E-09	5.03E-10	9.56E-05	0.12	47.58	1.19	-2.34	1.14E-01	-1.19E-03	-1.88E-01	7.62E-10	2.38E-10	7.98E-05	0.12	47.60	1.17	-2.51	1.13E-01	-1.18E-03	3.72E-01	1.03E-09	3.29E-10	9.83E-05	0.12	47.68	1.17	-2.36	1.07E-01	-1.12E-03	3.48E-01	1.21E-08	4.12E-09	3.26E-04	0.11	47.36	1.25
WM Bundles																																								
ARCL	-1.97	9.54E-02	-1.05E-03	2.40E-01	4.30E-07	5.92E-08	1.40E-02	0.09	45.55	1.45	-1.16	6.14E-02	-6.87E-04	-1.25E-01	1.45E-03	5.06E-04	1.33E-02	0.05	44.57	1.87	-1.22	5.86E-02	-6.56E-04	2.35E-01	2.21E-03	8.00E-04	1.90E-02	0.04	44.52	9.47	-2.12	1.01E-01	-1.10E-03	2.19E-01	8.44E-08	1.44E-08	2.51E-02	0.09	46.18	1.35
ARCR	-1.26	6.01E-02	-6.70E-04	2.47E-01	1.70E-03	6.11E-04	1.36E-02	0.04	44.69	1.89	-1.72	1.02E-01	-1.22E-03	-7.67E-02	2.53E-08	8.43E-11	1.01E-01	0.17	41.63	1.51	-1.74	9.85E-02	-1.18E-03	1.52E-01	5.40E-08	2.22E-10	1.02E-01	0.17	41.57	1.53	-1.44	6.62E-02	-7.17E-04	2.57E-01	5.40E-04	2.44E-04	1.02E-02	0.05	46.05	2.39
ATRL	-1.74	9.89E-02	-1.19E-03	1.56E-01	4.61E-08	1.78E-10	9.44E-02	0.17	41.53	1.56	-1.76	1.02E-01	-1.21E-03	-1.26E-01	1.93E-08	9.91E-11	6.99E-03	0.17	42.14	1.44	-1.84	9.98E-02	-1.18E-03	2.44E-01	3.83E-08	2.31E-10	9.27E-03	0.16	42.11	1.47	-2.03	1.10E-01	-1.28E-03	1.58E-01	1.76E-09	9.14E-12	9.03E-02	0.16	42.87	1.27
ATRR	-1.84	9.99E-02	-1.18E-03	2.48E-01	3.61E-08	2.13E-10	8.09E-03	0.16	42.09	1.47	-2.01	9.90E-02	-1.05E-03	-1.53E-01	1.75E-07	5.34E-08	1.78E-03	0.09	47.00	1.39	-2.02	9.32E-02	-9.97E-04	2.96E-01	8.24E-07	2.45E-07	2.56E-03	0.08	46.72	1.52	-1.50	8.35E-02	-1.01E-03	2.59E-01	4.93E-06	6.48E-08	6.38E-03	0.14	40.99	1.92
CGCR	-2.12	9.65E-02	-1.02E-03	3.13E-01	3.25E-07	1.16E-07	1.39E-03	0.09	47.17	1.45	-2.00	9.84E-02	-1.04E-03	-1.54E-01	2.12E-07	7.27E-08	1.76E-03	0.09	47.07	1.43	-2.02	9.26E-02	-9.87E-04	2.94E-01	9.92E-07	3.32E-07	2.77E-03	0.08	46.90	1.50	-1.97	9.07E-02	-9.68E-04	2.89E-01	1.70E-06	5.67E-07	3.35E-03	0.08	46.82	1.55
CSTL	-2.04	9.61E-02	-1.03E-03	2.37E-01	4.00E-07	9.90E-08	1.56E-02	0.08	46.49	1.47	-1.98	9.89E-02	-1.06E-03	-1.22E-01	2.42E-07	5.90E-08	1.36E-02	0.09	46.41	1.43	-2.00	9.42E-02	-1.01E-03	2.33E-01	6.76E-07	1.65E-07	1.79E-02	0.08	46.34	1.50	-2.50	1.16E-01	-1.22E-03	2.08E-01	8.05E-10	2.75E-10	3.15E-02	0.10	47.61	1.18
CSTR	-1.56	6.72E-02	-7.05E-04	3.62E-01	4.12E-04	2.84E-04	2.88E-04	0.06	47.61	5.24	-1.37	6.73E-02	-7.08E-04	-1.79E-01	4.34E-04	2.88E-04	3.70E-04	0.06	47.56	2.36	-1.49	6.47E-02	-6.82E-04	3.42E-01	6.87E-04	4.58E-04	6.36E-04	0.05	47.50	2.69	-1.74	7.47E-02	-7.72E-04	3.41E-01	8.75E-05	7.07E-05	6.22E-04	0.06	48.51	2.16
FA	-0.63	4.56E-02	-6.28E-04	1.51E-01	1.43E-02	9.77E-04	1.20E-01	0.09	35.66	3.31	-0.62	4.88E-02	-6.65E-04	-7.80E-02	8.90E-03	5.09E-04	1.10E-01	0.10	35.72	6.02	-0.66	4.67E-02	-6.38E-04	1.47E-01	1.21E-02	8.14E-04	1.32E-01	0.09	36.14	3.11	-0.88	5.55E-02	-7.14E-04	1.41E-01	3.01E-03	1.91E-04	1.50E-01	0.09	38.16	4.11
FP	-0.08	2.14E-02	-3.64E-04	-1.20E-02	2.58E-01	5.93E-02	9.04E-01	0.06	27.78	8.22	-0.13	2.35E-02	-3.88E-04	6.24E-03	2.14E-01	4.52E-02	9.00E-01	0.06	29.02	7.35	-0.10	2.22E-02	-3.71E-04	-1.01E-02	2.40E-01	5.51E-02	9.19E-01	0.06	28.60	7.87	-0.26	2.99E-02	-4.48E-04	-2.45E-02	1.13E-01	2.02E-02	8.04E-01	0.06	31.65	6.96
IFOL	-1.61	8.71E-02	-1.03E-03	2.03E-01	2.56E-06	5.97E-08	3.49E-02	0.12	42.10	1.87	-1.55	8.98E-02	-1.06E-03	-1.02E-01	1.31E-06	2.63E-08	3.35E-02	0.13	42.14	1.82	-1.60	8.68E-02	-1.02E-03	1.98E-01	2.84E-06	6.83E-08	3.95E-02	0.12	42.11	1.91	-1.70	8.96E-02	-1.04E-03	2.00E-01	1.51E-06	5.14E-08	3.86E-02	0.11	42.95	1.73
IFOR	-1.24	7.08E-02	-8.75E-04	2.28E-01	1.28E-04	3.82E-06	1.78E-02	0.11	40.11	2.62	-1.21	7.54E-02	-9.27E-04	-1.12E-01	4.92E-05	1.20E-06	1.99E-02	0.12	40.40	2.34	-1.24	7.10E-02	-8.76E-04	2.20E-01	1.25E-04	3.89E-06	2.28E-02	0.11	40.20	2.59	-1.27	7.16E-02	-8.76E-04	2.13E-01	1.16E-04	4.19E-06	2.74E-02	0.11	40.57	2.44

ILFL	-1.51	7.80E-02	-8.89E-04	1.44E-01	3.93E-05	4.64E-06	1.43E-01	0.07	43.68	1.98	-1.46	7.98E-02	-9.10E-04	-7.40E-02	2.73E-05	2.99E-06	1.34E-01	0.07	43.62	1.99	-1.49	7.74E-02	-8.83E-04	1.42E-01	4.53E-05	5.42E-06	1.51E-01	0.07	43.64	2.02	-1.66	8.38E-02	-9.35E-04	1.36E-01	1.05E-05	1.53E-06	1.69E-01	0.07	44.71	1.71
ILFR	-1.11	6.07E-02	-7.40E-04	2.35E-01	1.24E-03	1.20E-04	1.70E-02	0.08	40.48	3.52	-1.04	6.37E-02	-7.74E-04	-1.17E-01	7.60E-04	6.68E-05	1.82E-02	0.08	40.70	3.55	-1.10	6.08E-02	-7.40E-04	2.28E-01	1.24E-03	1.22E-04	2.04E-02	0.08	40.52	3.64	-1.47	7.61E-02	-8.77E-04	2.08E-01	5.63E-05	5.67E-06	3.42E-02	0.08	43.11	2.26
pARCL	-2.76	1.21E-01	-1.22E-03	2.56E-01	1.35E-10	2.29E-10	8.16E-03	0.11	49.73	1.25	-2.62	1.20E-01	-1.21E-03	-1.27E-01	1.67E-10	2.86E-10	8.70E-03	0.11	49.74	1.26	-2.65	1.16E-01	-1.17E-03	2.46E-01	7.54E-10	1.09E-09	1.13E-02	0.10	49.55	1.32	-2.89	1.26E-01	-1.26E-03	2.29E-01	2.02E-11	4.83E-11	1.75E-02	0.12	50.08	1.22
pARCR	-2.24	1.01E-01	-1.05E-03	2.83E-01	1.11E-07	6.13E-08	3.88E-03	0.09	47.99	1.39	-2.13	1.02E-01	-1.07E-03	-1.41E-01	8.22E-08	4.52E-08	4.26E-03	0.09	47.92	1.37	-2.14	9.66E-02	-1.01E-03	2.66E-01	3.63E-07	1.87E-07	6.92E-03	0.08	47.76	1.44	-2.48	1.10E-01	-1.12E-03	2.75E-01	6.25E-09	5.83E-09	4.73E-03	0.10	48.94	1.26
SLFL	-2.31	1.08E-01	-1.14E-03	2.22E-01	1.18E-08	3.16E-09	2.28E-02	0.10	47.13	1.28	-2.20	1.08E-01	-1.15E-03	-1.12E-01	1.12E-08	2.95E-09	2.21E-02	0.10	47.10	1.28	-2.27	1.06E-01	-1.13E-03	2.18E-01	1.93E-08	5.10E-09	2.56E-02	0.09	47.03	1.30	-2.55	1.18E-01	-1.24E-03	2.03E-01	3.97E-10	1.36E-10	3.60E-02	0.11	47.71	1.17
SLFR	-1.57	7.17E-02	-7.84E-04	3.46E-01	1.54E-04	5.15E-05	4.92E-04	0.07	45.62	2.85	-1.42	7.31E-02	-8.00E-04	-1.71E-01	1.24E-04	4.07E-05	6.19E-04	0.07	45.56	2.31	-1.53	7.03E-02	-7.70E-04	3.34E-01	2.13E-04	7.08E-05	7.79E-04	0.07	45.41	2.43	-1.99	8.90E-02	-9.38E-04	3.39E-01	2.55E-06	1.21E-06	5.76E-04	0.08	47.49	1.73

GAM: generalized additive models; cGM: cortical grey matter; sWM: superficial white matter; BA: Brodmann Area; WM: white matter; FA: Callosum Forceps Minor; FP: Callosum Forceps Major; ARC: Arcuate; pARC: Posterior Arcuate Fasciculus; ATR: Thalamic Radiation; CGC: Cingulum Cingulate; CST: Corticospinal; IFO: Inferior Fronto-Occipital Fasciculus; ILF: Inferior Longitudinal Fasciculus; SLF: Superior Longitudinal Fasciculus.L: leftl; R: right.

S. 3.4 Comparison of Peak Age

Structure	Comparision	ρ	p	Slope	Intercept
R1					
cGM	B-spline vs GAM	0.881	<0.001	0.51	27.24
cGM	Raw vs GAM	0.870	<0.001	1.09	-3.70
cGM	Raw vs B-spline	0.709	<0.001	1.32	-15.11
sWM	B-spline vs GAM	0.875	<0.001	0.65	15.78
sWM	Raw vs GAM	0.850	<0.001	0.91	3.87
sWM	Raw vs B-spline	0.850	<0.001	1.25	-11.92
WMbundles	B-spline vs GAM	0.804	<0.001	0.45	18.26
WMbundles	Raw vs GAM	0.671	0.001	0.78	4.39
WMbundles	Raw vs B-spline	0.829	<0.001	1.41	-18.63
R2*					
cGM	B-spline vs GAM	0.964	<0.001	1.15	-8.86
cGM	Raw vs GAM	0.978	<0.001	0.90	5.38
cGM	Raw vs B-spline	0.989	<0.001	0.75	14.83
sWM	B-spline vs GAM	0.981	<0.001	1.00	-0.52
sWM	Raw vs GAM	0.991	<0.001	1.00	-0.37
sWM	Raw vs B-spline	0.987	<0.001	0.96	2.15
WMbundles	B-spline vs GAM	0.970	<0.001	1.01	-1.60
WMbundles	Raw vs GAM	0.996	<0.001	0.98	0.88
WMbundles	Raw vs B-spline	0.967	<0.001	0.92	4.51

Pairwise comparisons include Raw versus HBR, Raw versus GAM, and HBR versus GAM. Spearman correlation analyses were conducted for ROI wise peak age estimates across tissue types and pairwise comparisons between raw and harmonized data. Spearman's ρ denotes the rank correlation coefficient. In addition, ordinary least squares linear regression was fitted separately within each tissue type for each pairwise comparison, and the resulting slope and intercept are reported. Notably, due to heterogeneous site-specific age ranges in the raw R1 data, differences in regression slopes derived from quadratic model fitting (e.g., Raw–HBR in R1) may reflect trajectory irregularities and should not be interpreted as standalone measures of harmonization performance. *GAM: generalized additive models; cGM: cortical grey matter; sWM: superficial white matter;*

S. 3.4 Comparison of Peak Age

Structure	Comparision	ρ	p	Slope	Intercept
R1					
cGM	B-spline vs GAM	0.881	<0.001	0.51	27.24
cGM	Raw vs GAM	0.870	<0.001	1.09	-3.70
cGM	Raw vs B-spline	0.709	<0.001	1.32	-15.11
sWM	B-spline vs GAM	0.875	<0.001	0.65	15.78
sWM	Raw vs GAM	0.850	<0.001	0.91	3.87
sWM	Raw vs B-spline	0.850	<0.001	1.25	-11.92
WMbundles	B-spline vs GAM	0.804	<0.001	0.45	18.26
WMbundles	Raw vs GAM	0.671	0.001	0.78	4.39
WMbundles	Raw vs B-spline	0.829	<0.001	1.41	-18.63
R2*					
cGM	B-spline vs GAM	0.964	<0.001	1.15	-8.86
cGM	Raw vs GAM	0.978	<0.001	0.90	5.38
cGM	Raw vs B-spline	0.989	<0.001	0.75	14.83
sWM	B-spline vs GAM	0.981	<0.001	1.00	-0.52
sWM	Raw vs GAM	0.991	<0.001	1.00	-0.37
sWM	Raw vs B-spline	0.987	<0.001	0.96	2.15
WMbundles	B-spline vs GAM	0.970	<0.001	1.01	-1.60
WMbundles	Raw vs GAM	0.996	<0.001	0.98	0.88
WMbundles	Raw vs B-spline	0.967	<0.001	0.92	4.51

Pairwise comparisons include Raw versus HBR, Raw versus GAM, and HBR versus GAM. Spearman correlation analyses were conducted for ROI wise peak age estimates across tissue types and pairwise comparisons between raw and harmonized data. Spearman's ρ denotes the rank correlation coefficient. In addition, ordinary least squares linear regression was fitted separately within each tissue type for each pairwise comparison, and the resulting slope and intercept are reported.*Notably, due to heterogeneous site-specific age ranges in the raw R1 data, differences in regression slopes derived from quadratic model fitting (e.g., Raw–HBR in R1) may reflect trajectory irregularities and should not be interpreted as standalone measures of harmonization performance. GAM: generalized additive models; cGM: cortical grey matter; sWM: superficial white matter;*

S. 3.5 RMSE Comparison of Polynomial Age-Fitting Model Before and After Harmonization

	Raw	Combat	(Combat-Raw)/Raw	GAM	(GAM-Raw)/Raw	B-spline	(B-spline-Raw)/Raw
R1							
cGM							
BA6	0.013	0.013	-0.5%	0.011	-13.6%	0.011	-15.0%
BA4	0.017	0.017	0.9%	0.015	-12.5%	0.015	-13.1%
BA9	0.011	0.012	5.0%	0.010	-7.9%	0.010	-13.0%
BA3	0.017	0.015	-13.3%	0.014	-20.1%	0.013	-22.6%
BA1	0.017	0.017	-4.2%	0.015	-12.7%	0.014	-18.2%
BA5	0.014	0.013	-6.8%	0.012	-17.9%	0.012	-17.8%
BA7	0.012	0.012	-1.0%	0.011	-13.7%	0.010	-14.1%
BA2	0.013	0.013	-0.5%	0.011	-14.2%	0.011	-13.9%
BA31	0.012	0.011	-11.1%	0.010	-20.4%	0.009	-26.3%
BA40	0.011	0.011	0.4%	0.009	-12.7%	0.009	-13.6%
BA44	0.011	0.011	2.1%	0.010	-10.3%	0.010	-12.9%
BA45	0.011	0.012	5.1%	0.011	-5.9%	0.010	-9.4%
BA23	0.012	0.011	-10.2%	0.010	-17.6%	0.009	-23.0%
BA39	0.011	0.010	-2.1%	0.010	-10.4%	0.009	-13.1%
BA43	0.012	0.012	2.9%	0.011	-5.1%	0.011	-7.0%
BA19	0.012	0.011	-4.7%	0.010	-14.1%	0.010	-16.0%
BA47	0.011	0.012	4.2%	0.011	-5.0%	0.011	-5.2%
BA41	0.013	0.012	-6.2%	0.011	-14.7%	0.011	-17.4%
BA30	0.013	0.012	-10.4%	0.011	-13.9%	0.012	-10.7%
BA22	0.010	0.010	4.1%	0.009	-2.9%	0.009	-6.3%
BA42	0.015	0.014	-4.6%	0.013	-10.6%	0.014	-8.7%
BA21	0.010	0.010	-0.9%	0.009	-8.4%	0.009	-11.1%
BA38	0.009	0.009	-0.1%	0.008	-8.6%	0.008	-6.4%
BA37	0.010	0.010	2.0%	0.009	-7.9%	0.009	-12.9%
BA20	0.010	0.009	-9.8%	0.008	-13.1%	0.008	-16.1%
BA32	0.010	0.010	1.7%	0.010	-5.6%	0.009	-9.6%
BA24	0.010	0.010	-4.9%	0.009	-13.9%	0.009	-16.8%
BA10	0.012	0.012	1.9%	0.011	-4.7%	0.011	-5.5%
BA25	0.016	0.012	-22.6%	0.010	-38.0%	0.009	-43.4%
BA11	0.010	0.011	5.3%	0.010	1.0%	0.010	-0.7%
BA46	0.011	0.012	5.0%	0.010	-6.7%	0.010	-9.3%
BA17	0.016	0.014	-14.8%	0.013	-19.3%	0.013	-19.7%
BA18	0.014	0.013	-9.7%	0.012	-17.6%	0.011	-20.4%
BA27	0.012	0.012	-4.0%	0.012	-5.4%	0.012	-1.6%
BA36	0.011	0.011	-0.4%	0.010	-14.0%	0.010	-12.2%
BA35	0.013	0.012	-10.3%	0.011	-13.4%	0.012	-11.0%
BA28	0.015	0.016	1.4%	0.015	-3.2%	0.015	-4.9%
BA29	0.014	0.013	-6.8%	0.012	-12.7%	0.013	-12.2%
BA26	0.021	0.021	-2.4%	0.020	-8.2%	0.019	-10.2%
BA33	0.010	0.010	0.4%	0.010	-1.7%	0.009	-7.4%
sWM							
BA6	0.033	0.032	-3.2%	0.030	-8.4%	0.031	-5.0%
BA4	0.033	0.032	-5.5%	0.029	-12.0%	0.030	-11.4%
BA9	0.034	0.033	-1.2%	0.031	-7.0%	0.033	-0.5%
BA3	0.035	0.029	-17.1%	0.028	-21.1%	0.029	-18.3%
BA1	0.036	0.032	-11.7%	0.030	-18.1%	0.032	-11.2%
BA5	0.038	0.036	-6.5%	0.033	-12.9%	0.035	-9.4%
BA7	0.036	0.033	-7.6%	0.032	-11.5%	0.035	-2.5%
BA2	0.036	0.032	-10.2%	0.031	-12.9%	0.033	-7.7%
BA31	0.034	0.033	-2.3%	0.032	-5.2%	0.034	0.3%
BA40	0.036	0.034	-4.3%	0.032	-9.6%	0.033	-6.7%
BA44	0.032	0.031	-4.6%	0.030	-7.7%	0.031	-3.6%
BA45	0.034	0.033	-1.4%	0.032	-5.7%	0.033	-2.1%
BA23	0.034	0.034	1.6%	0.033	-0.6%	0.036	6.5%
BA39	0.034	0.032	-5.3%	0.031	-7.9%	0.033	-2.0%
BA43	0.036	0.036	-0.9%	0.035	-3.8%	0.038	5.0%
BA19	0.032	0.030	-5.9%	0.029	-9.0%	0.030	-7.7%
BA47	0.035	0.035	1.8%	0.033	-5.3%	0.033	-5.4%
BA41	0.033	0.032	-1.3%	0.032	-3.6%	0.033	0.6%
BA30	0.035	0.036	1.2%	0.035	-1.0%	0.039	9.9%

BA22	0.034	0.034	1.6%	0.033	-1.9%	0.035	3.8%
BA42	0.033	0.031	-8.2%	0.030	-9.2%	0.032	-2.8%
BA21	0.034	0.036	4.1%	0.034	0.2%	0.036	3.9%
BA38	0.036	0.037	4.9%	0.036	0.1%	0.037	4.3%
BA37	0.034	0.035	2.9%	0.034	-0.9%	0.034	0.1%
BA20	0.035	0.036	4.3%	0.035	0.5%	0.036	5.1%
BA32	0.034	0.034	-0.6%	0.032	-4.3%	0.033	-1.8%
BA24	0.033	0.033	0.2%	0.032	-2.5%	0.035	5.5%
BA10	0.033	0.034	1.7%	0.032	-3.2%	0.034	1.9%
BA25	0.045	0.046	0.9%	0.043	-4.5%	0.047	4.3%
BA11	0.032	0.033	5.2%	0.032	1.2%	0.035	9.2%
BA46	0.034	0.033	-5.0%	0.031	-8.8%	0.033	-3.5%
BA17	0.034	0.030	-12.0%	0.029	-14.7%	0.031	-6.9%
BA18	0.033	0.029	-11.5%	0.027	-17.3%	0.028	-14.6%
BA27	0.044	0.044	-0.9%	0.044	0.8%	0.047	6.3%
BA36	0.031	0.032	1.8%	0.031	-1.7%	0.031	0.1%
BA35	0.032	0.032	-0.9%	0.030	-4.7%	0.031	-3.8%
BA28	0.034	0.034	1.1%	0.032	-4.1%	0.032	-4.6%
BA29	0.043	0.044	2.8%	0.042	-0.1%	0.046	7.7%
BA26	0.045	0.046	3.0%	0.045	-0.3%	0.049	9.2%
BA33	0.040	0.041	1.4%	0.040	-1.6%	0.040	-0.6%
WM Bundles							
ARCR	0.034	0.034	0.1%	0.033	-1.5%	0.034	0.6%
ATRL	0.036	0.037	1.7%	0.036	-0.3%	0.037	2.3%
ATRR	0.036	0.037	2.4%	0.036	1.1%	0.037	3.9%
CGCL	0.036	0.036	0.9%	0.035	-2.1%	0.035	-2.1%
CGCR	0.035	0.036	0.7%	0.035	-2.4%	0.034	-3.5%
CSTL	0.031	0.031	0.8%	0.030	-2.7%	0.030	-1.5%
CSTR	0.031	0.031	-0.3%	0.030	-3.1%	0.032	3.6%
FA	0.037	0.038	0.9%	0.037	-0.9%	0.037	0.2%
FP	0.035	0.035	0.3%	0.035	0.4%	0.035	2.0%
IFOL	0.034	0.035	2.4%	0.034	1.0%	0.037	8.3%
IFOR	0.035	0.035	1.3%	0.035	0.1%	0.036	3.0%
ILFL	0.034	0.035	2.3%	0.034	0.1%	0.034	-0.6%
ILFR	0.034	0.034	-0.1%	0.033	-2.2%	0.034	-1.0%
pARCL	0.034	0.035	1.9%	0.034	0.1%	0.034	-0.5%
pARCR	0.035	0.035	-1.4%	0.034	-2.4%	0.033	-5.4%
SLFL	0.033	0.033	1.3%	0.033	-0.2%	0.035	5.0%
SLFR	0.034	0.034	-0.3%	0.033	-2.8%	0.034	1.4%
*R2**							
cGM							
BA6	0.665	0.642	-3.5%	0.673	1.1%	0.669	0.5%
BA4	0.809	0.795	-1.7%	0.829	2.5%	0.801	-1.0%
BA9	0.640	0.608	-5.0%	0.625	-2.4%	0.603	-5.8%
BA3	0.797	0.790	-1.0%	0.781	-2.1%	0.767	-3.8%
BA1	0.831	0.786	-5.4%	0.788	-5.2%	0.760	-8.5%
BA5	0.707	0.691	-2.3%	0.698	-1.3%	0.674	-4.8%
BA7	0.723	0.698	-3.5%	0.712	-1.6%	0.681	-5.9%
BA2	0.743	0.716	-3.6%	0.754	1.5%	0.729	-1.9%
BA31	1.001	0.950	-5.0%	0.986	-1.5%	0.965	-3.5%
BA40	0.751	0.688	-8.4%	0.723	-3.6%	0.713	-5.1%
BA44	0.776	0.722	-7.0%	0.749	-3.6%	0.700	-9.8%
BA45	1.116	0.986	-11.7%	0.869	-22.2%	0.810	-27.4%
BA23	1.157	1.107	-4.3%	1.128	-2.5%	1.081	-6.6%
BA39	0.848	0.786	-7.3%	0.814	-4.0%	0.787	-7.2%
BA43	0.780	0.739	-5.3%	0.752	-3.6%	0.720	-7.7%
BA19	0.781	0.739	-5.5%	0.758	-2.9%	0.742	-5.1%
BA47	2.216	1.501	-32.3%	1.066	-51.9%	1.017	-54.1%
BA41	0.857	0.826	-3.7%	0.866	1.1%	0.840	-2.0%
BA30	1.010	0.982	-2.8%	0.993	-1.7%	0.998	-1.1%
BA22	1.002	0.745	-25.7%	0.750	-25.2%	0.725	-27.7%
BA42	0.961	0.896	-6.7%	0.904	-6.0%	0.876	-8.9%
BA21	2.985	1.327	-55.5%	1.041	-65.1%	0.995	-66.7%
BA38	3.534	1.514	-57.1%	1.039	-70.6%	1.001	-71.7%

BA37	1.071	0.887	-17.2%	0.853	-20.3%	0.841	-21.5%
BA20	1.876	1.088	-42.0%	1.045	-44.3%	1.006	-46.4%
BA32	0.781	0.736	-5.7%	0.758	-2.9%	0.738	-5.5%
BA24	0.987	0.855	-13.3%	0.894	-9.4%	0.878	-11.0%
BA10	0.829	0.745	-10.1%	0.748	-9.7%	0.718	-13.4%
BA25	3.163	1.807	-42.9%	1.634	-48.3%	1.676	-47.0%
BA11	1.786	1.077	-39.7%	1.034	-42.1%	1.018	-43.0%
BA46	0.851	0.786	-7.6%	0.797	-6.3%	0.750	-11.8%
BA17	0.924	0.910	-1.5%	0.954	3.2%	0.943	2.0%
BA18	0.830	0.804	-3.2%	0.828	-0.3%	0.814	-2.0%
BA27	1.413	1.280	-9.4%	1.306	-7.5%	1.288	-8.8%
BA36	3.205	1.523	-52.5%	1.199	-62.6%	1.185	-63.0%
BA35	1.356	1.311	-3.3%	1.362	0.4%	1.356	0.0%
BA28	2.227	1.430	-35.8%	1.248	-43.9%	1.234	-44.6%
BA29	1.642	1.532	-6.7%	1.534	-6.6%	1.484	-9.6%
BA26	1.953	1.854	-5.1%	1.776	-9.1%	1.694	-13.3%
BA33	1.262	1.023	-18.9%	1.007	-20.2%	1.010	-19.9%
sWM							
BA6	0.723	0.711	-1.7%	0.715	-1.1%	0.678	-6.2%
BA4	0.787	0.772	-1.9%	0.787	0.0%	0.766	-2.7%
BA9	0.759	0.742	-2.3%	0.765	0.7%	0.745	-1.9%
BA3	0.728	0.715	-1.8%	0.723	-0.8%	0.694	-4.6%
BA1	0.796	0.782	-1.8%	0.779	-2.1%	0.780	-2.0%
BA5	0.794	0.790	-0.5%	0.795	0.1%	0.785	-1.1%
BA7	0.783	0.772	-1.4%	0.786	0.4%	0.787	0.5%
BA2	0.775	0.754	-2.7%	0.788	1.7%	0.762	-1.7%
BA31	0.997	0.967	-3.0%	0.985	-1.2%	0.970	-2.7%
BA40	0.803	0.773	-3.7%	0.801	-0.3%	0.773	-3.8%
BA44	0.855	0.824	-3.6%	0.845	-1.1%	0.821	-4.0%
BA45	1.095	1.091	-0.3%	1.026	-6.3%	0.971	-11.3%
BA23	1.127	1.107	-1.8%	1.150	2.0%	1.126	-0.1%
BA39	0.862	0.811	-5.9%	0.835	-3.2%	0.806	-6.6%
BA43	0.950	0.871	-8.3%	0.881	-7.2%	0.865	-9.0%
BA19	0.818	0.793	-3.1%	0.811	-0.8%	0.806	-1.4%
BA47	1.779	1.525	-14.3%	1.197	-32.7%	1.153	-35.2%
BA41	0.904	0.887	-1.9%	0.909	0.6%	0.877	-3.0%
BA30	1.034	1.010	-2.3%	1.031	-0.2%	0.990	-4.3%
BA22	0.838	0.820	-2.2%	0.831	-0.8%	0.822	-1.9%
BA42	0.864	0.854	-1.1%	0.878	1.6%	0.852	-1.4%
BA21	1.912	1.224	-36.0%	1.063	-44.4%	1.036	-45.8%
BA38	2.764	1.525	-44.8%	1.114	-59.7%	1.073	-61.2%
BA37	0.915	0.911	-0.5%	0.868	-5.1%	0.881	-3.7%
BA20	1.209	1.022	-15.5%	1.001	-17.3%	0.980	-19.0%
BA32	0.845	0.802	-5.0%	0.828	-2.0%	0.825	-2.3%
BA24	0.901	0.863	-4.3%	0.890	-1.2%	0.876	-2.9%
BA10	0.846	0.806	-4.8%	0.827	-2.2%	0.804	-5.0%
BA25	2.270	1.805	-20.5%	1.483	-34.7%	1.472	-35.1%
BA11	1.181	0.986	-16.5%	0.966	-18.1%	0.959	-18.8%
BA46	0.892	0.838	-6.0%	0.855	-4.2%	0.826	-7.4%
BA17	0.882	0.873	-1.1%	0.910	3.1%	0.903	2.4%
BA18	0.799	0.789	-1.2%	0.810	1.4%	0.796	-0.3%
BA27	1.370	1.366	-0.3%	1.339	-2.3%	1.302	-5.0%
BA36	2.447	1.425	-41.8%	1.205	-50.7%	1.182	-51.7%
BA35	1.299	1.258	-3.1%	1.292	-0.5%	1.227	-5.5%
BA28	1.476	1.335	-9.5%	1.230	-16.6%	1.252	-15.1%
BA29	1.336	1.326	-0.7%	1.306	-2.2%	1.251	-6.3%
BA26	1.425	1.421	-0.3%	1.413	-0.8%	1.357	-4.8%
BA33	1.052	1.047	-0.5%	1.052	-0.1%	1.052	-0.1%
WM Bundles							
ARCL	0.988	0.948	-4.1%	0.985	-0.4%	0.969	-2.0%
ARCR	1.032	1.002	-2.9%	1.028	-0.3%	1.003	-2.8%
ATRL	1.070	1.047	-2.1%	1.070	0.0%	1.082	1.1%
ATRR	0.997	0.962	-3.5%	1.008	1.1%	0.991	-0.7%
CGCR	1.009	0.979	-3.0%	1.032	2.2%	1.045	3.6%

CSTL	0.989	0.933	-5.7%	0.953	-3.7%	0.927	-6.3%
CSTR	0.982	0.923	-6.1%	0.961	-2.1%	0.939	-4.4%
FA	1.098	1.080	-1.7%	1.111	1.1%	1.098	-0.1%
FP	1.360	1.285	-5.5%	1.323	-2.7%	1.306	-4.0%
IFOL	1.023	0.992	-3.1%	1.019	-0.4%	0.998	-2.4%
IFOR	1.038	0.994	-4.2%	1.035	-0.2%	1.036	-0.2%
ILFL	1.067	1.063	-0.3%	1.090	2.2%	1.059	-0.7%
ILFR	1.038	1.024	-1.3%	1.046	0.8%	1.041	0.3%
pARCL	0.933	0.922	-1.2%	0.959	2.8%	0.952	2.0%
pARCR	0.988	0.968	-2.0%	1.019	3.2%	1.001	1.4%
SLFL	0.954	0.924	-3.2%	0.943	-1.1%	0.928	-2.7%
SLFR	0.937	0.893	-4.7%	0.918	-2.0%	0.899	-4.1%

RMSE: Root-mean-square error; GAM: generalized additive models; cGM: cortical grey matter; sWM: superficial white matter; Brodmann Area: BA; WM: white matter; FA: Callosum Forceps Minor; FP: Callosum Forceps Major; ARC: Arcuate; pARC: Posterior Arcuate Fasciculus; ATR: Thalamic Radiation; CGC: Cingulum Cingulate; CST: Corticospinal; IFO: Inferior Fronto-Occipital Fasciculus; ILF: Inferior Longitudinal Fasciculus; SLF: Superior Longitudinal Fasciculus.L: leftl; R: right.

S. 3.6 Cohen’s d Effect Sizes for Regional Group Differences in qMRI Measures

Raw Data, Cross-site (pwMS from site 2, HC from site 1)

	R1						R2*					
Region	Mean (Controls)	SD (Controls)	Mean (Patients)	SD (Patients)	Cohen's D	*p* (FDR)	Mean (Controls)	SD (Controls)	Mean (Patients)	SD (Patients)	Cohen's D	*p* (FDR)
cGM												
BA1	0.75	0.02	0.76	0.02	-0.68	1.65E-08	18.38	0.88	18.35	0.98	-0.12	3.44E-01
BA2	0.71	0.01	0.71	0.01	-0.05	6.68E-01	18.07	0.86	17.86	0.72	0.02	8.77E-01
BA3	0.74	0.02	0.76	0.01	-1.17	2.29E-21	18.16	0.81	17.79	0.95	0.32	5.95E-03
BA4	0.75	0.02	0.76	0.02	-0.65	7.41E-08	19.25	1.04	18.88	0.88	0.02	9.13E-01
BA5	0.71	0.01	0.70	0.01	0.25	4.06E-02	17.19	0.78	17.07	0.84	-0.05	7.22E-01
BA6	0.71	0.02	0.71	0.01	0.00	9.76E-01	17.58	0.88	17.37	0.72	-0.11	3.66E-01
BA7	0.71	0.01	0.70	0.01	0.21	9.05E-02	18.08	0.82	17.88	0.73	0.02	8.77E-01
BA8	0.70	0.02	0.69	0.01	0.20	1.05E-01	16.82	0.81	16.79	0.65	-0.28	1.73E-02
BA9	0.69	0.01	0.68	0.01	0.47	8.02E-05	16.57	0.75	16.59	0.60	-0.29	1.31E-02
BA10	0.70	0.01	0.68	0.01	0.84	3.59E-12	17.14	0.84	17.61	0.90	-0.73	6.88E-10
BA11	0.69	0.01	0.68	0.01	0.27	2.74E-02	17.75	1.13	20.71	1.11	-2.50	3.07E-75
BA17	0.73	0.01	0.74	0.02	-0.85	3.59E-12	19.83	1.02	19.51	0.79	0.18	1.44E-01
BA18	0.73	0.01	0.73	0.01	-0.53	8.22E-06	19.97	0.95	19.75	0.80	0.01	9.54E-01
BA19	0.71	0.01	0.71	0.01	-0.13	3.28E-01	19.09	0.88	18.97	0.75	-0.12	3.44E-01
BA20	0.66	0.01	0.66	0.01	-0.56	3.03E-06	16.76	1.19	20.14	1.44	-2.75	4.28E-86
BA21	0.67	0.01	0.67	0.01	0.09	4.82E-01	17.04	1.16	22.71	1.95	-3.57	1.93E-122
BA22	0.69	0.01	0.69	0.01	0.05	6.68E-01	17.69	0.92	18.62	0.83	-1.35	1.37E-28
BA23	0.68	0.01	0.69	0.01	-0.61	4.06E-07	17.64	1.21	17.85	0.95	-0.36	2.43E-03
BA24	0.67	0.01	0.67	0.01	-0.56	2.39E-06	16.03	0.99	16.82	0.72	-1.08	1.65E-19
BA25	0.64	0.01	0.65	0.01	-0.85	3.59E-12	16.27	1.67	21.99	2.22	-2.76	1.86E-86
BA26	0.69	0.02	0.70	0.02	-0.59	7.63E-07	19.01	1.85	20.14	2.08	-0.72	1.06E-09
BA27	0.69	0.01	0.70	0.01	-0.62	2.03E-07	17.71	1.38	18.53	1.20	-0.70	2.62E-09
BA28	0.69	0.02	0.69	0.01	-0.19	1.23E-01	16.63	1.32	19.99	2.47	-1.85	7.38E-48
BA29	0.68	0.01	0.68	0.01	-0.37	1.91E-03	17.82	1.58	18.82	1.60	-0.75	2.85E-10
BA30	0.68	0.01	0.69	0.01	-0.41	6.96E-04	17.50	1.06	17.71	0.92	-0.37	1.84E-03
BA31	0.69	0.01	0.69	0.01	-0.48	5.47E-05	17.75	1.08	17.93	0.79	-0.36	2.28E-03
BA32	0.68	0.01	0.67	0.01	0.07	5.71E-01	16.41	0.87	16.50	0.66	-0.36	2.24E-03
BA33	0.65	0.01	0.65	0.01	-0.57	1.58E-06	15.04	1.07	16.65	1.15	-1.59	2.60E-37
BA35	0.67	0.01	0.68	0.01	-0.83	7.53E-12	18.07	1.46	18.30	1.09	-0.36	2.27E-03
BA36	0.68	0.01	0.68	0.01	-0.34	5.37E-03	16.86	1.28	22.89	2.38	-3.23	1.21E-107
BA37	0.69	0.01	0.68	0.01	-0.03	7.87E-01	18.33	1.00	19.18	1.05	-1.13	4.47E-21
BA38	0.66	0.01	0.65	0.01	-0.19	1.23E-01	15.50	1.10	22.26	2.99	-3.37	1.84E-113
BA39	0.70	0.01	0.69	0.01	0.20	1.01E-01	18.11	0.95	18.22	0.91	-0.36	2.27E-03
BA40	0.70	0.01	0.69	0.01	0.27	2.67E-02	17.51	0.85	17.64	0.77	-0.44	2.57E-04
BA41	0.70	0.01	0.70	0.01	-0.71	3.72E-09	17.95	0.97	17.85	0.74	-0.12	3.62E-01
BA42	0.72	0.02	0.73	0.02	-0.64	1.17E-07	18.57	1.06	18.39	0.97	-0.09	5.03E-01
BA43	0.70	0.01	0.70	0.01	-0.14	2.85E-01	17.59	0.89	17.71	0.80	-0.41	6.16E-04
BA44	0.70	0.01	0.70	0.01	-0.09	4.80E-01	17.75	0.91	17.96	0.71	-0.59	6.06E-07
BA45	0.71	0.01	0.70	0.01	0.10	4.79E-01	17.72	0.96	18.94	1.39	-1.26	2.65E-25
BA46	0.70	0.01	0.69	0.01	0.35	3.65E-03	17.51	0.91	17.82	0.79	-0.59	5.21E-07
BA47	0.70	0.01	0.69	0.01	0.27	2.93E-02	17.67	1.14	21.54	3.07	-1.97	1.07E-52
sWM												
BA1	1.04	0.03	1.08	0.03	-0.84	1.09E-11	20.34	0.90	19.93	0.91	0.24	3.83E-02
BA2	1.07	0.04	1.11	0.03	-0.72	3.73E-09	20.25	0.88	19.17	0.69	1.07	2.56E-19
BA3	1.05	0.03	1.08	0.03	-1.11	2.58E-19	20.40	0.82	19.34	0.78	1.06	6.98E-19
BA4	1.07	0.04	1.10	0.03	-0.50	3.08E-05	20.31	0.93	19.21	0.76	0.99	5.43E-17
BA5	1.03	0.04	1.06	0.04	-0.50	2.85E-05	19.48	0.88	18.73	0.80	0.72	5.64E-10
BA6	1.07	0.04	1.09	0.03	-0.22	7.58E-02	19.71	0.82	18.81	0.72	0.93	2.39E-15
BA7	1.05	0.04	1.08	0.04	-0.58	1.49E-06	20.27	0.89	19.31	0.72	0.95	7.94E-16
BA8	1.06	0.04	1.07	0.04	-0.10	4.29E-01	19.33	0.90	18.67	0.73	0.65	1.52E-08
BA9	1.06	0.04	1.07	0.04	-0.10	4.29E-01	19.46	0.87	18.58	0.72	0.98	1.49E-16
BA10	1.06	0.04	1.06	0.04	0.22	7.53E-02	20.10	0.92	19.21	0.92	0.91	6.63E-15
BA11	1.05	0.04	1.04	0.04	0.47	9.13E-05	20.14	1.04	21.35	1.10	-1.04	2.24E-18
BA17	1.00	0.03	1.03	0.04	-0.65	1.02E-07	20.64	0.99	19.75	0.74	0.74	1.67E-10
BA18	1.04	0.03	1.08	0.04	-0.80	6.44E-11	21.13	0.94	20.12	0.75	0.88	3.76E-14
BA19	1.08	0.04	1.10	0.04	-0.38	2.12E-03	20.93	0.91	19.70	0.71	1.20	2.47E-23
BA20	1.02	0.04	1.00	0.04	0.62	4.19E-07	19.50	1.08	20.43	1.25	-0.89	2.77E-14
BA21	1.05	0.04	1.05	0.04	0.23	6.89E-02	20.35	1.15	23.25	1.83	-1.99	3.28E-53
BA22	1.09	0.04	1.09	0.04	0.04	7.70E-01	20.99	0.94	19.93	0.84	0.97	2.66E-16
BA23	1.06	0.04	1.07	0.04	0.08	5.22E-01	20.23	1.21	19.16	0.97	0.86	2.22E-13
BA24	1.07	0.04	1.08	0.04	0.16	1.99E-01	19.70	0.97	18.58	0.73	1.07	2.56E-19
BA25	0.97	0.05	0.95	0.05	0.56	3.62E-06	18.76	1.53	21.30	2.12	-1.32	4.96E-27
BA26	1.01	0.05	1.00	0.05	0.33	7.04E-03	19.35	1.44	19.05	1.46	0.13	2.71E-01
BA27	0.97	0.05	0.96	0.05	0.11	3.83E-01	19.30	1.42	18.62	1.25	0.31	5.90E-03
BA28	1.02	0.03	0.99	0.05	0.61	5.73E-07	19.25	1.31	20.01	2.18	-0.53	3.60E-06
BA29	1.00	0.05	0.99	0.05	0.29	1.67E-02	19.12	1.33	18.35	1.18	0.48	2.63E-05
BA30	1.06	0.04	1.07	0.04	0.15	2.34E-01	19.72	1.08	18.63	0.96	0.95	8.51E-16
BA31	1.07	0.04	1.09	0.04	-0.11	3.84E-01	20.44	1.06	19.19	0.86	1.07	2.60E-19
BA32	1.08	0.04	1.09	0.04	0.02	8.89E-01	19.82	0.95	18.61	0.71	1.29	4.73E-26
BA33	1.03	0.04	1.02	0.05	0.30	1.57E-02	18.70	1.15	18.53	1.16	0.13	2.53E-01
BA35	1.03	0.03	1.02	0.04	0.29	1.76E-02	19.80	1.34	18.36	1.16	0.92	5.81E-15
BA36	0.96	0.03	0.94	0.04	0.60	7.94E-07	18.73	1.27	22.68	2.42	-2.21	2.44E-62
BA37	1.07	0.04	1.07	0.04	0.25	4.69E-02	20.81	0.97	20.13	0.98	0.56	1.34E-06
BA38	0.98	0.04	0.95	0.04	0.62	3.30E-07	18.89	1.17	23.62	2.98	-2.38	1.80E-69
BA39	1.07	0.04	1.10	0.04	-0.32	8.23E-03	20.72	0.92	19.39	0.86	1.27	1.49E-25
BA40	1.04	0.04	1.07	0.04	-0.33	7.34E-03	20.17	0.89	19.03	0.81	1.12	9.45E-21
BA41	1.09	0.04	1.10	0.04	-0.05	7.18E-01	20.28	0.96	19.20	0.78	1.07	3.51E-19
BA42	1.09	0.04	1.12	0.03	-0.54	8.61E-06	20.81	0.99	19.72	0.84	0.93	3.03E-15
BA43	1.06	0.04	1.07	0.04	-0.15	2.53E-01	20.20	0.97	18.74	0.95	1.32	5.89E-27
BA44	1.09	0.04	1.11	0.03	-0.36	3.11E-03	20.68	0.91	19.70	0.74	0.97	1.63E-16
BA45	1.05	0.04	1.07	0.04	-0.25	4.69E-02	20.50	1.06	20.57	1.58	-0.09	4.16E-01
BA46	1.07	0.04	1.09	0.04	-0.37	2.47E-03	20.53	0.91	19.45	0.80	1.12	1.35E-20
BA47	1.05	0.04	1.06	0.04	-0.04	7.53E-01	20.53	1.22	22.47	2.96	-0.96	5.49E-16
WM Bundles												
ARCL	1.16	0.04	1.14	0.04	0.60	4.32E-07	21.05	1.08	19.82	0.86	1.18	6.54E-23
ARCR	1.17	0.04	1.16	0.04	0.55	3.43E-06	20.65	1.03	19.37	0.99	1.23	1.46E-24
ATRL	1.16	0.05	1.17	0.04	0.19	9.42E-02	21.20	1.17	20.24	1.09	1.01	8.61E-18
ATRR	1.17	0.05	1.16	0.04	0.39	7.53E-04	20.96	1.21	20.08	1.10	0.90	1.08E-14
CGCL	1.14	0.04	1.13	0.04	0.34	2.77E-03	20.51	1.08	19.26	0.94	1.16	2.48E-22
CGCR	1.14	0.04	1.13	0.04	0.44	1.61E-04	20.43	1.10	19.18	0.79	1.16	2.14E-22
CSTL	1.13	0.04	1.12	0.04	0.62	1.97E-07	19.65	1.02	18.35	0.97	1.24	8.56E-25
CSTR	1.14	0.04	1.13	0.04	0.53	5.56E-06	19.75	1.01	18.46	1.00	1.16	1.87E-22
FA	1.19	0.05	1.18	0.05	0.44	1.61E-04	21.21	1.20	20.35	1.25	0.85	2.46E-13
FP	1.14	0.04	1.12	0.05	1.00	2.54E-16	21.61	1.40	19.73	1.20	1.47	1.59E-32

Raw Data, Within-site (pwMS, HC both from site 2)

	R1						R2*					
Region	Mean (Controls)	SD (Controls)	Mean (Patients)	SD (Patients)	Cohen's D	*p* (FDR)	Mean (Controls)	SD (Controls)	Mean (Patients)	SD (Patients)	Cohen's D	*p* (FDR)
cGM												
BA1	0.76	0.02	0.76	0.02	0.33	1.38E-01	18.83	0.86	18.35	0.98	0.59	2.74E-04
BA2	0.72	0.01	0.71	0.01	0.39	1.38E-01	18.31	0.70	17.86	0.72	0.72	1.53E-05
BA3	0.76	0.02	0.76	0.01	0.17	3.26E-01	18.29	0.84	17.79	0.95	0.55	6.53E-04
BA4	0.76	0.02	0.76	0.02	0.21	2.64E-01	19.15	0.93	18.88	0.88	0.46	3.42E-03
BA5	0.70	0.01	0.70	0.01	0.27	1.68E-01	17.35	0.72	17.07	0.84	0.40	1.18E-02
BA6	0.71	0.02	0.71	0.01	0.32	1.38E-01	17.62	0.71	17.37	0.72	0.49	2.03E-03
BA7	0.71	0.01	0.70	0.01	0.30	1.39E-01	18.28	0.78	17.88	0.73	0.62	1.44E-04
BA8	0.70	0.02	0.69	0.01	0.39	1.38E-01	17.04	0.71	16.79	0.65	0.49	2.08E-03
BA9	0.68	0.01	0.68	0.01	0.32	1.38E-01	16.83	0.67	16.59	0.60	0.48	2.46E-03
BA10	0.68	0.01	0.68	0.01	0.28	1.68E-01	17.83	0.82	17.61	0.90	0.30	6.59E-02
BA11	0.68	0.01	0.68	0.01	0.25	1.90E-01	20.95	1.25	20.71	1.11	0.20	1.96E-01
BA17	0.74	0.01	0.74	0.02	0.31	1.38E-01	19.98	0.86	19.51	0.79	0.65	7.98E-05
BA18	0.73	0.01	0.73	0.01	0.31	1.38E-01	20.18	0.85	19.75	0.80	0.61	1.44E-04
BA19	0.71	0.01	0.71	0.01	0.26	1.68E-01	19.40	0.86	18.97	0.75	0.67	6.90E-05
BA20	0.66	0.01	0.66	0.01	-0.13	4.23E-01	20.01	1.40	20.14	1.44	-0.05	7.72E-01
BA21	0.66	0.01	0.67	0.01	-0.02	8.98E-01	23.27	1.97	22.71	1.95	0.28	8.72E-02
BA22	0.69	0.01	0.69	0.01	0.20	2.75E-01	18.97	0.92	18.62	0.83	0.49	2.03E-03
BA23	0.69	0.01	0.69	0.01	0.01	9.69E-01	18.11	1.15	17.85	0.95	0.31	6.30E-02
BA24	0.67	0.01	0.67	0.01	0.04	8.01E-01	16.91	0.81	16.82	0.72	0.20	1.96E-01
BA25	0.65	0.01	0.65	0.01	-0.16	3.37E-01	22.48	2.29	21.99	2.22	0.21	1.77E-01
BA26	0.69	0.02	0.70	0.02	-0.22	2.48E-01	20.04	2.10	20.14	2.08	-0.03	8.39E-01
BA27	0.69	0.02	0.70	0.01	-0.28	1.68E-01	18.86	1.28	18.53	1.20	0.29	7.23E-02
BA28	0.69	0.02	0.69	0.01	0.20	2.64E-01	20.46	2.03	19.99	2.47	0.20	1.96E-01
BA29	0.68	0.01	0.68	0.01	0.07	6.95E-01	18.91	1.60	18.82	1.60	0.09	5.71E-01
BA30	0.69	0.01	0.69	0.01	0.16	3.37E-01	17.82	1.02	17.71	0.92	0.17	2.71E-01
BA31	0.69	0.01	0.69	0.01	0.16	3.37E-01	18.21	0.93	17.93	0.79	0.37	1.92E-02
BA32	0.67	0.01	0.67	0.01	0.15	3.80E-01	16.77	0.82	16.50	0.66	0.48	2.08E-03
BA33	0.65	0.01	0.65	0.01	-0.19	2.98E-01	16.61	1.13	16.65	1.15	0.00	9.75E-01
BA35	0.68	0.01	0.68	0.01	-0.05	7.98E-01	18.51	1.22	18.30	1.09	0.26	1.12E-01
BA36	0.69	0.01	0.68	0.01	0.33	1.38E-01	23.45	2.35	22.89	2.38	0.24	1.25E-01
BA37	0.68	0.01	0.68	0.01	0.11	5.09E-01	19.39	1.17	19.18	1.05	0.27	9.31E-02
BA38	0.65	0.01	0.65	0.01	-0.08	6.46E-01	22.94	2.67	22.26	2.99	0.24	1.26E-01
BA39	0.69	0.01	0.69	0.01	0.22	2.48E-01	18.57	0.86	18.22	0.91	0.49	2.03E-03
BA40	0.69	0.01	0.69	0.01	0.27	1.68E-01	17.94	0.75	17.64	0.77	0.50	2.03E-03
BA41	0.70	0.01	0.70	0.01	0.18	3.01E-01	18.24	0.76	17.85	0.74	0.61	1.44E-04
BA42	0.73	0.02	0.73	0.02	0.26	1.68E-01	19.06	0.99	18.39	0.97	0.76	8.74E-06
BA43	0.70	0.02	0.70	0.01	0.20	2.64E-01	17.90	0.78	17.71	0.80	0.31	5.87E-02
BA44	0.70	0.01	0.70	0.01	0.23	2.35E-01	18.08	0.82	17.96	0.71	0.25	1.13E-01
BA45	0.70	0.01	0.70	0.01	0.18	2.99E-01	18.69	1.40	18.94	1.39	-0.15	3.46E-01
BA46	0.70	0.01	0.69	0.01	0.32	1.38E-01	18.03	0.84	17.82	0.79	0.29	7.20E-02
BA47	0.69	0.02	0.69	0.01	0.21	2.60E-01	21.24	2.49	21.54	3.07	-0.11	4.71E-01
sWM												
BA1	1.09	0.03	1.08	0.03	0.55	1.46E-04	20.47	0.87	19.93	0.91	0.71	5.01E-06
BA2	1.13	0.03	1.11	0.03	0.65	1.39E-05	19.68	0.69	19.17	0.69	0.80	5.29E-07
BA3	1.10	0.03	1.08	0.03	0.51	4.26E-04	19.88	0.75	19.34	0.78	0.76	1.30E-06
BA4	1.12	0.03	1.10	0.03	0.66	1.23E-05	19.73	0.82	19.21	0.76	0.76	1.30E-06
BA5	1.08	0.03	1.06	0.04	0.62	2.74E-05	19.24	0.81	18.73	0.80	0.66	1.64E-05
BA6	1.11	0.03	1.09	0.03	0.76	1.42E-06	19.27	0.77	18.81	0.72	0.69	8.49E-06
BA7	1.11	0.03	1.08	0.04	0.62	2.65E-05	19.84	0.82	19.31	0.72	0.74	2.41E-06
BA8	1.09	0.03	1.07	0.04	0.70	4.41E-06	19.13	0.82	18.67	0.73	0.64	2.51E-05
BA9	1.10	0.03	1.07	0.04	0.69	4.66E-06	19.08	0.73	18.58	0.72	0.71	4.78E-06
BA10	1.09	0.03	1.06	0.04	0.62	2.74E-05	19.53	0.79	19.21	0.92	0.39	9.26E-03
BA11	1.07	0.03	1.04	0.04	0.70	4.31E-06	21.71	1.07	21.35	1.10	0.31	3.62E-02
BA17	1.05	0.03	1.03	0.04	0.61	3.13E-05	20.17	0.84	19.75	0.74	0.65	2.01E-05
BA18	1.09	0.03	1.08	0.04	0.48	8.42E-04	20.62	0.84	20.12	0.75	0.72	3.69E-06
BA19	1.12	0.03	1.10	0.04	0.68	6.34E-06	20.29	0.83	19.70	0.71	0.83	3.76E-07
BA20	1.04	0.04	1.00	0.04	0.94	6.17E-09	20.84	1.22	20.43	1.25	0.38	1.11E-02
BA21	1.08	0.04	1.05	0.04	0.65	1.25E-05	23.82	1.72	23.25	1.83	0.32	3.37E-02
BA22	1.12	0.03	1.09	0.04	0.69	4.62E-06	20.46	0.90	19.93	0.84	0.66	1.64E-05
BA23	1.09	0.03	1.07	0.04	0.62	2.74E-05	19.72	1.04	19.16	0.97	0.56	2.19E-04
BA24	1.10	0.03	1.08	0.04	0.73	2.83E-06	18.99	0.78	18.58	0.73	0.56	1.96E-04
BA25	0.99	0.03	0.95	0.05	0.76	1.42E-06	22.19	2.63	21.30	2.12	0.38	1.14E-02
BA26	1.02	0.04	1.00	0.05	0.51	4.37E-04	19.13	1.48	19.05	1.46	0.08	6.11E-01
BA27	0.98	0.04	0.96	0.05	0.56	1.33E-04	19.09	1.55	18.62	1.25	0.37	1.19E-02
BA28	1.04	0.04	0.99	0.05	0.89	1.83E-08	20.47	1.74	20.01	2.18	0.25	8.43E-02
BA29	1.01	0.04	0.99	0.05	0.57	9.61E-05	18.68	1.45	18.35	1.18	0.27	6.88E-02
BA30	1.09	0.03	1.07	0.04	0.69	4.81E-06	19.19	0.99	18.63	0.96	0.57	1.70E-04
BA31	1.11	0.03	1.09	0.04	0.74	2.37E-06	19.75	0.97	19.19	0.86	0.63	3.38E-05
BA32	1.12	0.03	1.09	0.04	0.67	8.82E-06	19.23	0.76	18.61	0.71	0.84	3.76E-07
BA33	1.04	0.04	1.02	0.05	0.46	1.53E-03	18.93	1.10	18.53	1.16	0.39	9.63E-03
BA35	1.05	0.03	1.02	0.04	0.97	4.03E-09	18.85	1.18	18.36	1.16	0.45	2.61E-03
BA36	0.97	0.03	0.94	0.04	0.91	1.27E-08	23.30	2.13	22.68	2.42	0.26	7.40E-02
BA37	1.10	0.04	1.07	0.04	0.72	3.12E-06	20.72	1.19	20.13	0.98	0.60	7.78E-05
BA38	0.98	0.04	0.95	0.04	0.75	1.57E-06	24.25	2.62	23.62	2.98	0.23	1.08E-01
BA39	1.12	0.03	1.10	0.04	0.71	3.47E-06	19.91	0.81	19.39	0.86	0.66	1.64E-05
BA40	1.09	0.03	1.07	0.04	0.72	3.14E-06	19.55	0.79	19.03	0.81	0.70	5.62E-06
BA41	1.13	0.03	1.10	0.04	0.71	3.65E-06	19.85	0.86	19.20	0.78	0.81	4.82E-07
BA42	1.14	0.03	1.12	0.03	0.77	1.42E-06	20.32	0.86	19.72	0.84	0.78	8.50E-07
BA43	1.10	0.03	1.07	0.04	0.70	4.62E-06	19.19	0.91	18.74	0.95	0.51	6.29E-04
BA44	1.13	0.03	1.11	0.03	0.65	1.31E-05	20.06	0.82	19.70	0.74	0.48	1.46E-03
BA45	1.09	0.03	1.07	0.04	0.56	1.35E-04	20.48	1.41	20.57	1.58	-0.04	7.64E-01
BA46	1.12	0.03	1.09	0.04	0.64	1.63E-05	19.80	0.84	19.45	0.80	0.44	3.39E-03
BA47	1.09	0.03	1.06	0.04	0.58	6.90E-05	22.63	2.33	22.47	2.96	0.06	7.12E-01
WM Bundles												
ARCL	1.17	0.03	1.14	0.04	0.75	4.86E-07	20.57	0.85	19.82	0.86	0.85	2.17E-08
ARCR	1.18	0.03	1.16	0.04	0.74	6.35E-07	20.08	0.81	19.37	0.99	0.76	3.48E-07
ATRL	1.20	0.04	1.17	0.04	0.67	5.56E-06	20.92	0.94	20.24	1.09	0.63	1.97E-05
ATRR	1.19	0.04	1.16	0.04	0.66	5.69E-06	20.75	1.05	20.08	1.10	0.59	6.07E-05
CGCL	1.16	0.04	1.13	0.04	0.76	4.30E-07	19.96	0.80	19.26	0.94	0.78	2.15E-07
CGCR	1.16	0.04	1.13	0.04	0.77	4.13E-07	19.91	0.80	19.18	0.79	0.90	3.91E-09
CSTL	1.14	0.03	1.12	0.04	0.75	6.06E-07	18.95	0.86	18.35	0.97	0.63	1.77E-05
CSTR	1.16	0.03	1.13	0.04	0.71	1.42E-06	18.98	0.77	18.46	1.00	0.57	8.05E-05
FA	1.22	0.04	1.18	0.05	0.77	4.13E-07	21.04	0.98	20.35	1.25	0.58	6.47E-05
FP	1.16	0.04	1.12	0.05	1.02	5.35E-11	20.85	1.16	19.73	1.20	0.93	1.80E-09

Harmonized Data (across sites)

	R1						R2*					
Region	Mean (Controls)	SD (Controls)	Mean (Patients)	SD (Patients)	Cohen's D	*p* (FDR)	Mean (Controls)	SD (Controls)	Mean (Patients)	SD (Patients)	Cohen's D	*p* (FDR)
cGM												
BA1	0.75	0.02	0.74	0.02	0.27	5.16E-02	18.32	0.87	17.69	0.90	0.59	7.68E-07
BA2	0.71	0.01	0.71	0.01	0.35	1.71E-02	17.98	0.86	17.21	0.88	0.75	1.80E-09
BA3	0.74	0.02	0.73	0.01	0.09	4.74E-01	18.13	0.82	17.59	0.93	0.53	1.03E-05
BA4	0.75	0.02	0.75	0.02	0.12	3.55E-01	19.11	1.06	18.44	1.02	0.45	1.49E-04
BA5	0.71	0.01	0.70	0.01	0.22	1.03E-01	17.13	0.78	16.71	0.91	0.36	2.64E-03
BA6	0.71	0.02	0.71	0.01	0.25	7.21E-02	17.46	0.91	16.77	1.02	0.56	4.25E-06
BA7	0.71	0.01	0.70	0.01	0.35	1.71E-02	18.02	0.81	17.46	0.71	0.55	6.65E-06
BA8	0.70	0.02	0.69	0.01	0.31	3.47E-02	16.72	0.83	16.16	0.78	0.50	3.71E-05
BA9	0.69	0.01	0.69	0.01	0.27	5.16E-02	16.51	0.76	16.12	0.70	0.34	2.47E-03
BA10	0.70	0.01	0.69	0.01	0.30	3.47E-02	17.07	0.83	16.68	0.84	0.30	2.19E-02
BA11	0.69	0.01	0.68	0.01	0.21	1.13E-01	17.67	1.12	17.22	0.97	0.27	2.41E-02
BA17	0.73	0.01	0.72	0.02	0.29	3.47E-02	19.78	1.00	19.07	0.94	0.61	6.49E-07
BA18	0.72	0.01	0.72	0.01	0.29	3.47E-02	19.87	0.95	19.17	0.91	0.59	9.81E-07
BA19	0.71	0.01	0.70	0.01	0.36	1.71E-02	19.00	0.88	18.33	0.78	0.63	3.08E-07
BA20	0.66	0.01	0.66	0.01	-0.11	3.82E-01	16.64	1.18	16.37	1.19	0.02	8.80E-01
BA21	0.67	0.01	0.67	0.01	0.01	9.73E-01	16.92	1.16	16.38	1.28	0.26	2.60E-02
BA22	0.69	0.01	0.68	0.01	0.22	1.03E-01	17.59	0.93	17.00	0.86	0.47	9.14E-05
BA23	0.68	0.01	0.68	0.01	0.02	9.64E-01	17.59	1.18	17.12	1.09	0.27	2.19E-02
BA24	0.66	0.01	0.66	0.01	0.03	9.35E-01	15.93	0.99	15.48	0.88	0.30	1.11E-02
BA25	0.64	0.01	0.64	0.01	-0.19	1.42E-01	16.25	1.71	15.81	1.84	0.20	9.70E-02
BA26	0.68	0.02	0.69	0.02	-0.16	2.00E-01	18.89	1.79	18.70	1.73	-0.02	8.85E-01
BA27	0.69	0.01	0.69	0.01	-0.26	5.29E-02	17.63	1.38	17.02	1.27	0.34	4.50E-03
BA28	0.69	0.02	0.68	0.02	0.21	1.13E-01	16.55	1.31	15.93	1.61	0.35	3.84E-03
BA29	0.68	0.01	0.68	0.01	-0.01	9.78E-01	17.72	1.55	17.37	1.53	0.11	3.57E-01
BA30	0.68	0.01	0.68	0.01	0.10	4.32E-01	17.44	1.09	17.12	1.17	0.15	1.99E-01
BA31	0.69	0.01	0.68	0.01	0.18	1.51E-01	17.68	1.08	17.14	0.95	0.36	2.64E-03
BA32	0.68	0.01	0.67	0.01	0.18	1.51E-01	16.34	0.87	15.88	0.82	0.37	2.47E-03
BA33	0.65	0.01	0.65	0.01	-0.18	1.51E-01	14.95	1.07	14.74	1.08	0.05	6.71E-01
BA35	0.67	0.01	0.67	0.02	0.00	9.97E-01	17.99	1.46	17.49	1.33	0.21	7.41E-02
BA36	0.68	0.01	0.68	0.01	0.20	1.24E-01	16.77	1.29	16.23	1.28	0.29	1.40E-02
BA37	0.69	0.01	0.68	0.01	0.18	1.59E-01	18.22	1.01	17.73	0.97	0.31	4.59E-03
BA38	0.66	0.01	0.65	0.01	-0.01	9.73E-01	15.41	1.10	14.89	1.33	0.29	1.40E-02
BA39	0.70	0.01	0.69	0.01	0.27	5.29E-02	18.03	0.94	17.41	0.97	0.49	4.45E-05
BA40	0.69	0.01	0.69	0.01	0.30	3.47E-02	17.45	0.85	16.85	1.00	0.53	1.18E-05
BA41	0.70	0.01	0.69	0.01	0.15	2.51E-01	17.88	0.95	17.28	0.93	0.49	4.45E-05
BA42	0.72	0.02	0.71	0.01	0.21	1.13E-01	18.47	1.05	17.59	1.00	0.70	1.87E-08
BA43	0.70	0.01	0.69	0.01	0.22	1.03E-01	17.49	0.89	17.03	0.89	0.33	4.50E-03
BA44	0.70	0.01	0.69	0.01	0.32	3.47E-02	17.66	0.89	17.25	0.78	0.27	2.41E-02
BA45	0.71	0.01	0.70	0.01	0.24	7.90E-02	17.62	0.95	17.53	1.06	-0.11	3.75E-01
BA46	0.70	0.01	0.69	0.01	0.38	1.71E-02	17.43	0.89	17.09	0.80	0.21	7.16E-02
BA47	0.70	0.02	0.69	0.01	0.22	1.03E-01	17.59	1.12	17.44	1.35	-0.02	8.67E-01
sWM												
BA1	1.04	0.03	1.03	0.03	0.49	1.70E-05	20.28	0.91	19.54	1.00	0.65	3.66E-08
BA2	1.07	0.04	1.06	0.04	0.57	8.03E-07	20.22	0.86	19.52	0.83	0.70	7.10E-09
BA3	1.05	0.03	1.04	0.03	0.46	4.17E-05	20.35	0.81	19.68	0.79	0.70	7.10E-09
BA4	1.07	0.03	1.07	0.03	0.46	5.55E-05	20.25	0.93	19.57	0.84	0.59	5.95E-07
BA5	1.03	0.04	1.02	0.04	0.55	1.41E-06	19.45	0.87	18.89	0.85	0.55	2.05E-06
BA6	1.07	0.04	1.06	0.03	0.61	1.66E-07	19.69	0.80	19.18	0.75	0.52	8.95E-06
BA7	1.06	0.04	1.04	0.04	0.58	4.30E-07	20.23	0.89	19.50	0.87	0.71	6.20E-09
BA8	1.06	0.04	1.04	0.04	0.59	2.70E-07	19.32	0.88	18.85	0.75	0.46	8.38E-05
BA9	1.06	0.04	1.05	0.04	0.62	1.18E-07	19.47	0.85	18.97	0.84	0.56	2.05E-06
BA10	1.06	0.04	1.05	0.04	0.51	6.35E-06	20.12	0.89	19.87	0.95	0.27	1.73E-02
BA11	1.05	0.04	1.03	0.04	0.63	7.55E-08	20.12	1.02	19.71	1.04	0.36	1.87E-03
BA17	1.00	0.03	0.99	0.04	0.63	7.55E-08	20.58	0.98	19.93	0.89	0.55	2.05E-06
BA18	1.04	0.03	1.03	0.03	0.44	8.49E-05	21.05	0.94	20.30	0.86	0.67	2.17E-08
BA19	1.08	0.03	1.06	0.04	0.62	1.13E-07	20.88	0.91	20.06	0.83	0.80	1.00E-10
BA20	1.02	0.04	0.99	0.04	0.78	5.26E-11	19.45	1.08	18.91	1.13	0.37	1.48E-03
BA21	1.06	0.04	1.04	0.04	0.51	6.93E-06	20.33	1.13	19.92	1.30	0.26	2.29E-02
BA22	1.09	0.04	1.07	0.03	0.53	3.06E-06	20.95	0.94	20.34	0.91	0.54	3.82E-06
BA23	1.07	0.04	1.06	0.04	0.45	6.02E-05	20.21	1.19	19.54	1.17	0.54	3.82E-06
BA24	1.08	0.04	1.06	0.04	0.60	2.70E-07	19.66	0.95	19.06	0.89	0.56	1.74E-06
BA25	0.97	0.05	0.93	0.07	0.90	9.58E-14	18.81	1.52	18.32	1.29	0.36	1.75E-03
BA26	1.02	0.05	0.99	0.05	0.42	2.13E-04	19.30	1.40	19.12	1.45	0.07	5.41E-01
BA27	0.97	0.05	0.93	0.06	0.63	9.30E-08	19.20	1.41	18.53	1.13	0.36	1.57E-03
BA28	1.02	0.03	0.98	0.05	0.91	6.76E-14	19.20	1.36	18.67	1.82	0.27	1.74E-02
BA29	1.00	0.05	0.97	0.05	0.52	4.34E-06	19.09	1.29	18.72	1.08	0.23	4.67E-02
BA30	1.07	0.04	1.05	0.05	0.62	1.07E-07	19.71	1.04	19.11	0.97	0.54	3.82E-06
BA31	1.07	0.04	1.06	0.04	0.63	7.55E-08	20.40	1.05	19.72	0.94	0.58	8.59E-07
BA32	1.08	0.04	1.07	0.04	0.58	5.22E-07	19.84	0.93	19.20	0.83	0.67	2.17E-08
BA33	1.03	0.04	1.01	0.05	0.41	2.67E-04	18.69	1.12	18.25	1.20	0.37	1.52E-03
BA35	1.03	0.03	1.00	0.04	0.85	1.55E-12	19.72	1.30	19.04	1.16	0.42	2.62E-04
BA36	0.96	0.03	0.93	0.04	0.84	1.82E-12	18.63	1.28	17.93	1.49	0.42	3.01E-04
BA37	1.07	0.04	1.05	0.04	0.58	4.29E-07	20.78	0.99	20.12	0.93	0.58	8.59E-07
BA38	0.98	0.04	0.95	0.04	0.63	7.55E-08	18.82	1.17	18.30	1.37	0.32	5.67E-03
BA39	1.08	0.04	1.06	0.04	0.69	6.17E-09	20.68	0.91	20.05	0.97	0.57	1.28E-06
BA40	1.05	0.04	1.03	0.04	0.67	1.67E-08	20.13	0.87	19.46	0.93	0.67	2.17E-08
BA41	1.09	0.04	1.08	0.04	0.63	7.55E-08	20.27	0.93	19.68	0.81	0.61	3.13E-07
BA42	1.09	0.04	1.07	0.04	0.69	7.26E-09	20.74	0.97	19.84	1.01	0.79	1.00E-10
BA43	1.06	0.04	1.04	0.04	0.59	3.00E-07	20.19	0.95	19.68	1.02	0.45	1.10E-04
BA44	1.09	0.04	1.08	0.04	0.57	6.42E-07	20.67	0.89	20.21	0.88	0.45	9.76E-05
BA45	1.05	0.04	1.04	0.04	0.51	7.88E-06	20.48	1.03	20.52	1.21	-0.08	4.84E-01
BA46	1.07	0.04	1.06	0.04	0.60	1.92E-07	20.53	0.88	20.11	0.96	0.44	1.25E-04
BA47	1.06	0.04	1.04	0.04	0.55	1.31E-06	20.53	1.21	20.42	1.60	0.06	6.17E-01
WM Bundles							*Mean_Con*	*SD_Contro*	*Mean_Patient*	*SD_Patient*	*Cohens_d*	
ARCL	1.16	0.04	1.14	0.04	0.64	4.26E-08	21.05	1.05	20.20	1.04	0.83	1.16E-12
ARCR	1.17	0.04	1.15	0.04	0.65	2.11E-08	20.68	1.00	19.91	1.14	0.74	1.09E-10
ATRL	1.17	0.04	1.16	0.04	0.52	4.61E-06	21.28	1.13	20.77	1.14	0.57	5.78E-07
ATRR	1.17	0.04	1.16	0.04	0.55	1.01E-06	21.04	1.19	20.50	1.19	0.57	6.13E-07
CGCL	1.14	0.04	1.12	0.04	0.62	7.23E-08	20.51	1.05	19.72	1.23	0.70	9.24E-10
CGCR	1.14	0.04	1.13	0.04	0.60	1.28E-07	20.43	1.10	19.44	1.11	0.90	1.64E-14
CSTL	1.13	0.03	1.12	0.03	0.63	4.48E-08	19.67	0.99	19.11	1.06	0.55	1.22E-06
CSTR	1.14	0.03	1.13	0.03	0.61	8.59E-08	19.74	0.98	19.10	1.21	0.58	3.22E-07
FA	1.19	0.04	1.18	0.05	0.63	4.48E-08	21.27	1.17	20.76	1.30	0.53	3.13E-06
FP	1.15	0.04	1.12	0.05	1.04	9.57E-19	21.70	1.37	20.71	1.40	0.84	4.67E-13

IFOL	1.15	0.05	1.12	0.05	0.99	3.29E-16	21.24	1.12	19.60	1.08	1.54	4.48E-35	1.17	0.04	1.12	0.05	1.12	1.83E-12	20.88	0.94	19.60	1.08	1.24	1.94E-14	1.15	0.04	1.12	0.05	1.13	5.06E-21	21.30	1.08	20.13	1.13	1.16	4.04E-22
IFOR	1.15	0.04	1.13	0.06	0.97	5.82E-16	21.20	1.14	19.72	1.12	1.42	4.79E-31	1.18	0.04	1.13	0.06	1.11	1.83E-12	20.76	0.87	19.72	1.12	1.01	9.49E-11	1.16	0.04	1.13	0.05	1.04	9.57E-19	21.27	1.11	20.19	1.36	1.02	4.65E-18
ILFL	1.14	0.04	1.12	0.05	0.95	2.27E-15	20.88	1.15	19.57	1.14	1.16	1.87E-22	1.17	0.04	1.12	0.05	1.14	1.48E-12	20.91	1.00	19.57	1.14	1.22	2.55E-14	1.15	0.04	1.11	0.04	1.08	1.03E-19	20.92	1.11	19.67	1.17	1.16	4.04E-22
ILFR	1.16	0.04	1.13	0.06	0.86	3.50E-13	21.01	1.12	19.60	1.17	1.31	4.52E-27	1.18	0.04	1.13	0.06	1.06	1.00E-11	20.82	0.93	19.60	1.17	1.13	7.35E-13	1.16	0.04	1.12	0.05	1.12	5.06E-21	21.05	1.10	19.78	1.33	1.15	5.35E-22
pARCL	1.15	0.04	1.13	0.04	0.55	2.83E-06	20.39	1.03	19.25	0.87	1.04	8.70E-19	1.16	0.03	1.13	0.04	0.76	4.86E-07	20.04	0.83	19.25	0.87	0.93	1.64E-09	1.15	0.04	1.13	0.04	0.62	5.90E-08	20.41	1.02	19.48	1.05	0.85	3.47E-13
pARCR	1.16	0.04	1.15	0.04	0.44	1.61E-04	20.35	1.08	19.16	0.96	1.06	2.52E-19	1.18	0.03	1.15	0.04	0.78	4.13E-07	19.94	0.81	19.16	0.96	0.86	1.36E-08	1.16	0.04	1.14	0.04	0.67	1.23E-08	20.37	1.06	19.43	1.20	0.82	1.68E-12
SLFL	1.15	0.04	1.15	0.04	0.48	4.08E-05	20.98	1.01	19.80	0.91	1.13	1.18E-21	1.17	0.03	1.15	0.04	0.69	2.79E-06	20.48	0.88	19.80	0.91	0.74	6.72E-07	1.16	0.04	1.14	0.04	0.54	1.56E-06	20.98	0.99	20.23	1.00	0.74	1.51E-10
SLFR	1.17	0.04	1.16	0.04	0.42	2.91E-04	21.09	0.98	19.90	0.91	1.21	7.79E-24	1.19	0.03	1.16	0.04	0.68	3.98E-06	20.49	0.79	19.90	0.91	0.67	7.05E-06	1.17	0.04	1.16	0.04	0.56	7.96E-07	21.11	0.94	20.53	1.00	0.60	1.60E-07

All P values were FDR-adjusted and rounded to two decimal places. *GAM: generalized additive models; SD: standrad deviation; BA: Brodmann Area; cGM: cortical grey matter; sWM: superficial white matter; WM: white matter; FA: Callosum Forceps Minor; FP: Callosum Forceps Major; ARC: Arcuate; pARC: Posterior Arcuate Fasciculus; ATR: Thalamic Radiation; CGC: Cingulum Cingulate; CST: Corticospinal; IFO: Inferior Fronto-Occipital Fasciculus; ILF: Inferior Longitudinal Fasciculus; SLF: Superior Longitudinal Fasciculus.L: leftl; R: right.*

S. 4. eFigures

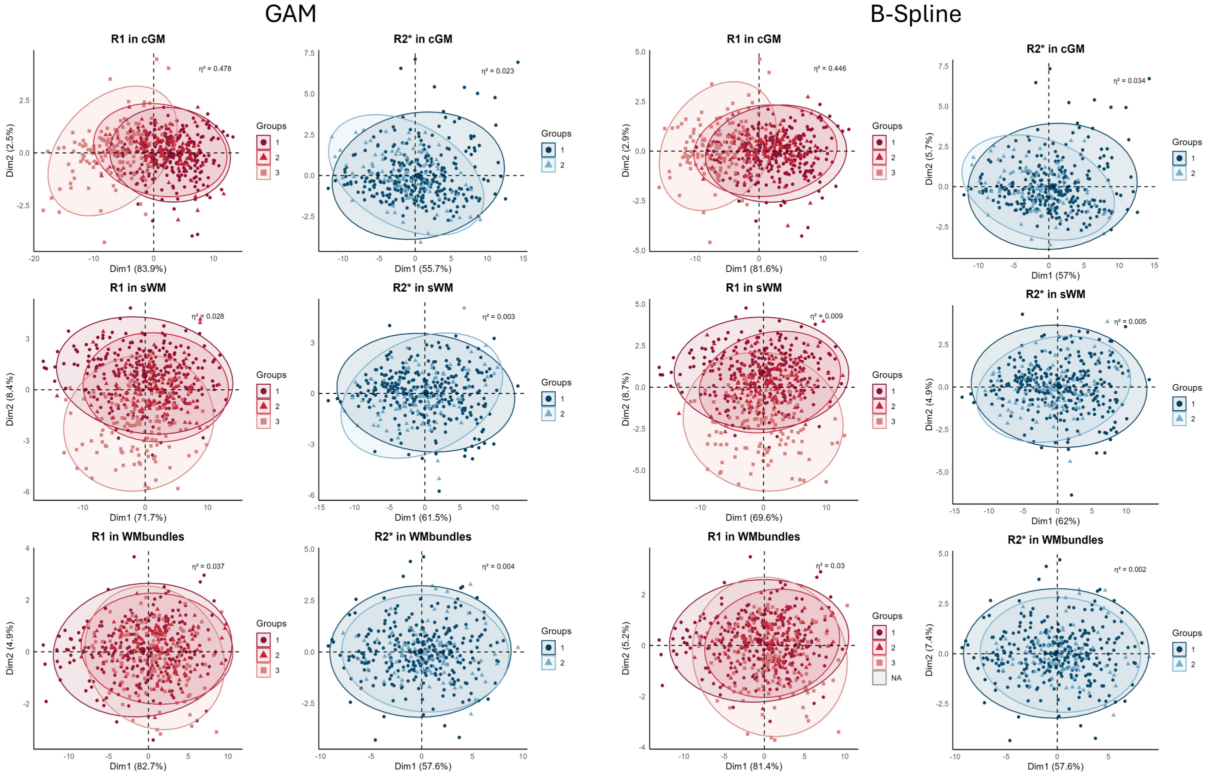

Figure 1 Principal Component Analysis of Site-Dependent Variance in R_1 and R_2* after Harmonization

The plot presents principal component analysis (PCA) illustrating site-related variance after Combat-GAM and HBR-based B-spline harmonizations, showing an apparent clustering of participants by site, especially along the first principal component. Each point represents one participant. Data from different sites are presented using different labels in the Group label. Ellipses represent the 95% confidence intervals for each site, and η^2 indicates the proportion of variance explained by the site effect. The observed site-related variance includes demographic differences across sites and should be interpreted alongside the quantitative ICC and η^2 results. cGM: cortical grey matter, sWM: superficial white matter, WM: white matter. PCA: principal component analysis. GAM: generalized additive model.

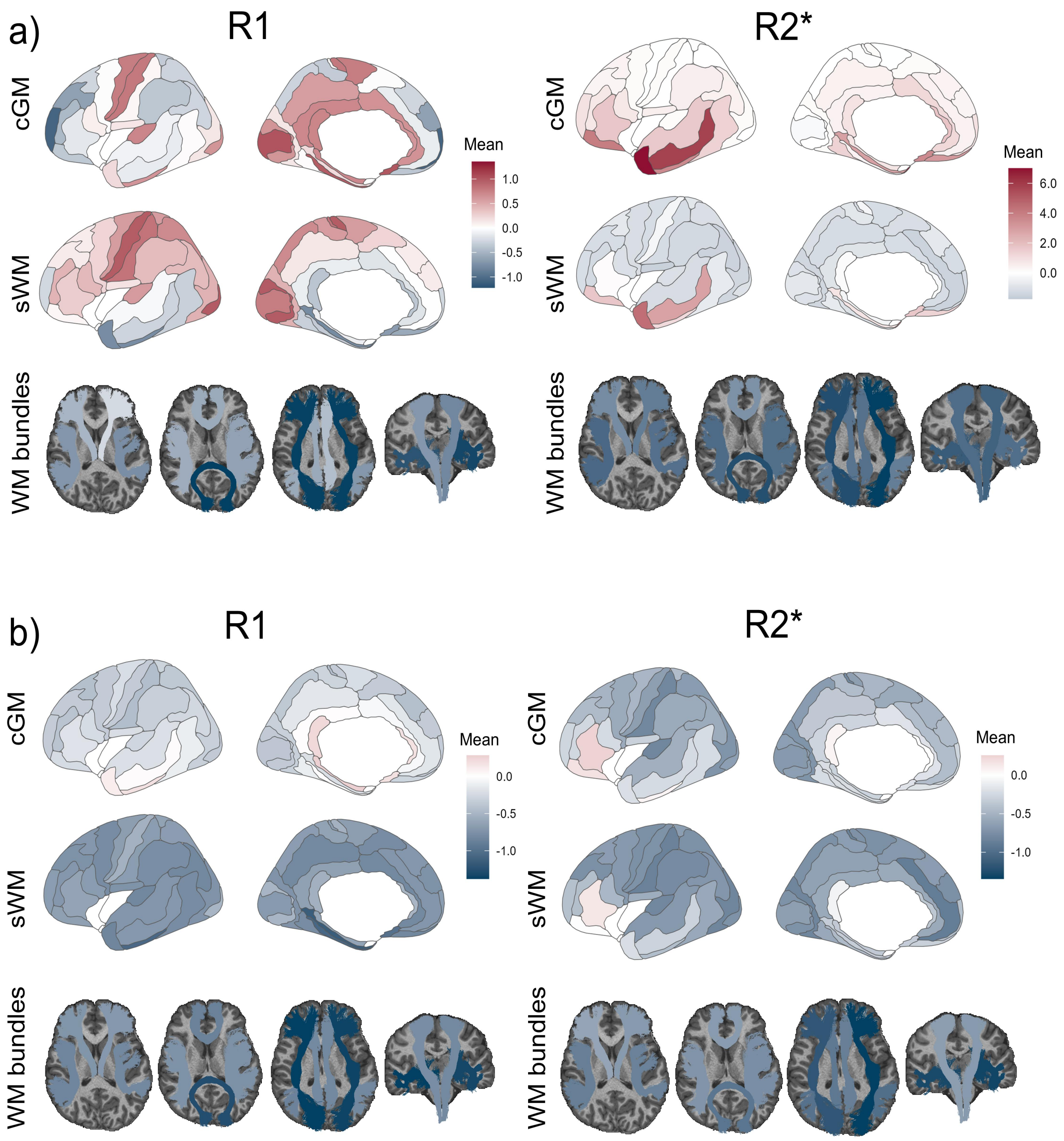

Figure 2. Regional Deviations in pwMS before Harmonization

Panel a) shows the cross-site comparison. Using raw healthy control data from Site 1 as the reference, the mean Z-scores of pwMS across regions in cGM, sWM, and WM bundles for both R_1 and R_2*represent deviations of pwMS relative to healthy controls acquired at a different site. Panel b) shows the within-site comparison: using raw healthy control data from Site 2 as the reference, regional mean Z-scores represent the deviations of pwMS relative to healthy controls from the same site. Z-scores were computed as descriptive baselines using polynomial regression models fitted in the healthy controls for each region and metric. Individual pwMS Z-scores were then calculated as the difference between the observed pwMS value and the

healthy control model prediction, divided by the residual standard deviation estimated from the reference healthy controls. Regional values shown in the maps correspond to the mean Z score across pwMS. The color bar indicates the mean Z -score, with warmer colors indicating more positive deviations and cooler colors indicating more negative deviations. cGM: cortical grey matter; sWM: superficial white matter; WM: white matter; pwMS: people with multiple sclerosis.
